# A Chemometrics-driven Strategy for the Bioactivity Evaluation of Complex Multicomponent Systems and the Effective Selection of Bioactivity-predictive Chemical Combinations

**DOI:** 10.1038/s41598-017-02499-1

**Published:** 2017-05-23

**Authors:** Yoshinori Fujimura, Chihiro Kawano, Ayaka Maeda-Murayama, Asako Nakamura, Akiko Koike-Miki, Daichi Yukihira, Eisuke Hayakawa, Takanori Ishii, Hirofumi Tachibana, Hiroyuki Wariishi, Daisuke Miura

**Affiliations:** 1 Innovation Center for Medical Redox Navigation, Kyushu University, 3-1-1 Maidashi, Higashi-ku, Fukuoka, 812-8582 Japan; 2 Okinawa Institute of Science, Technology Graduate University, 1919-1 Tancha, Onna-son, Kunigami-gun, Okinawa, 904-0495 Japan; 3 Faculty of Agriculture, Kyushu University, 6-10-1 Hakozaki, Higashi-ku, Fukuoka, 812-8581 Japan; 4 Faculty of Arts and Science, Kyushu University, 6-10-1 Hakozaki, Higashi-ku, Fukuoka, 812-8581 Japan

## Abstract

Although understanding their chemical composition is vital for accurately predicting the bioactivity of multicomponent drugs, nutraceuticals, and foods, no analytical approach exists to easily predict the bioactivity of multicomponent systems from complex behaviors of multiple coexisting factors. We herein represent a metabolic profiling (MP) strategy for evaluating bioactivity in systems containing various small molecules. Composition profiles of diverse bioactive herbal samples from 21 green tea extract (GTE) panels were obtained by a high-throughput, non-targeted analytical procedure. This employed the matrix-assisted laser desorption ionization–mass spectrometry (MALDI–MS) technique, using 1,5-diaminonaphthalene (1,5-DAN) as the optical matrix for detecting GTE-derived components. Multivariate statistical analyses revealed differences among the GTEs in their antioxidant activity, oxygen radical absorbance capacity (ORAC). A reliable bioactivity-prediction model was constructed to predict the ORAC of diverse GTEs from their compositional balance. This chemometric procedure allowed the evaluation of GTE bioactivity by multicomponent rather than single-component information. The bioactivity could be easily evaluated by calculating the summed abundance of a few selected components that contributed most to constructing the prediction model. 1,5-DAN-MALDI–MS-MP, using diverse bioactive sample panels, represents a promising strategy for screening bioactivity-predictive multicomponent factors and selecting effective bioactivity-predictive chemical combinations for crude multicomponent systems.

## Introduction

Various health-promoting physiological effects of multicomponent pharmaceuticals and nutraceuticals are generally evaluated by the activity and abundance of a single specific component (i.e. a low-molecular-weight bioactive compound); however, to accurately predict the real bioactivity of complicated multicomponent systems, the simultaneous evaluation of multiple coexisting factors is required^1^. Nevertheless, such an analytical approach remains to be established. Among the many analytical platforms, mass spectrometry (MS) is the most sensitive and selective technique for simultaneously determining a broad range of low-molecular-weight metabolites in medicinal plants, agricultural products, and foods, and thus it is the method of choice for metabolomic research. Metabolic profiling (MP) is often used to evaluate the genotype, origin, quality, and nutraceutical value of medicinal herbs and agricultural products by their compositional balance on the basis of the relative abundance of each metabolite to the total abundance of all metabolites^2–4^. Additionally, such a technique enables us to theoretically calculate the relative contribution of all multicomponent factors detected in crude samples to the total bioactivity. Considering the principle of this methodology, it is expected that MP may become an effective strategy for obtaining a comprehensive understanding of the physiological activity of multicomponent drugs and nutraceuticals. However, to date, there has been little research on the use of MP to compare or predict their bioactivity.

Conventional methods in which MS is coupled with pre-separation techniques, i.e. gas chromatography (GC)–MS and liquid chromatography (LC)–MS, have achieved great success in non-targeted applications of MP, but their major drawback lies in their limited ability to analyse large sets of samples and detect changes in their composition in a fast and simple way^5^. There is a clear need for more rapid, high-throughput MS approaches for MP. Currently, direct MS analysis is one of the most popular choices to achieve the maximum high-throughput production of information from the largest possible number of samples. Any separation step prior to MS detection is avoided, and thus direct analysis of the samples is achieved. Matrix-assisted laser desorption ionization (MALDI), a widely available ionization method used for direct MS analysis, offers several advantages for metabolite analysis, being a highly sensitive, high-throughput, and low sample-consuming (approximately 1 µL) technique compared with other ionization methods. However, the low ionization efficiency and the interference of matrix peaks from the use of conventional matrices hinder the detection of metabolite peaks. Recently, 9-aminoacridine (9-AA) was reported as a suitable matrix for metabolite analysis^6, 7^. When 9-AA was used in negative ion mode, only a few peaks derived from the matrix were observed in the low-mass range (*m*/*z* ≈ 500). In addition, the excellent ionization efficiency of 9-AA for important cellular metabolites (on the order of attomoles) was demonstrated^8^. We have developed a methodology for the rapid and direct analysis of cellular metabolites by MALDI–MS for high-throughput and non-targeted MP, and succeeded in applying this technique to evaluate the anti-cancer effect of a green tea polyphenol by visualizing metabolomic differences^6^. However, it remains unclear whether this technique can evaluate bioactivity through multicomponent information on crude herbal samples, such as green tea extract (GTE). Green tea is one of the most widely consumed beverages in the world and has shown various health-promoting effects^9, 10^. Therefore, in this study, we attempted to establish a rapid and simple MALDI–MS-MP technique for the chemometrics-driven evaluation of bioactivity based on composition profiles using diverse GTE panels with different antioxidant activity. Furthermore, this work investigated the applicability of such a technique for the selection of bioactivity-predictive or -discriminative multicomponent factors and the determination of a bioactivity-predictive chemical combination from multivariate data obtained by MALDI–MS measurement.

## Results

### Matrix screening-driven MALDI–MS-MP technique for quality evaluation of diverse GTEs with different properties

**Figure 1:**
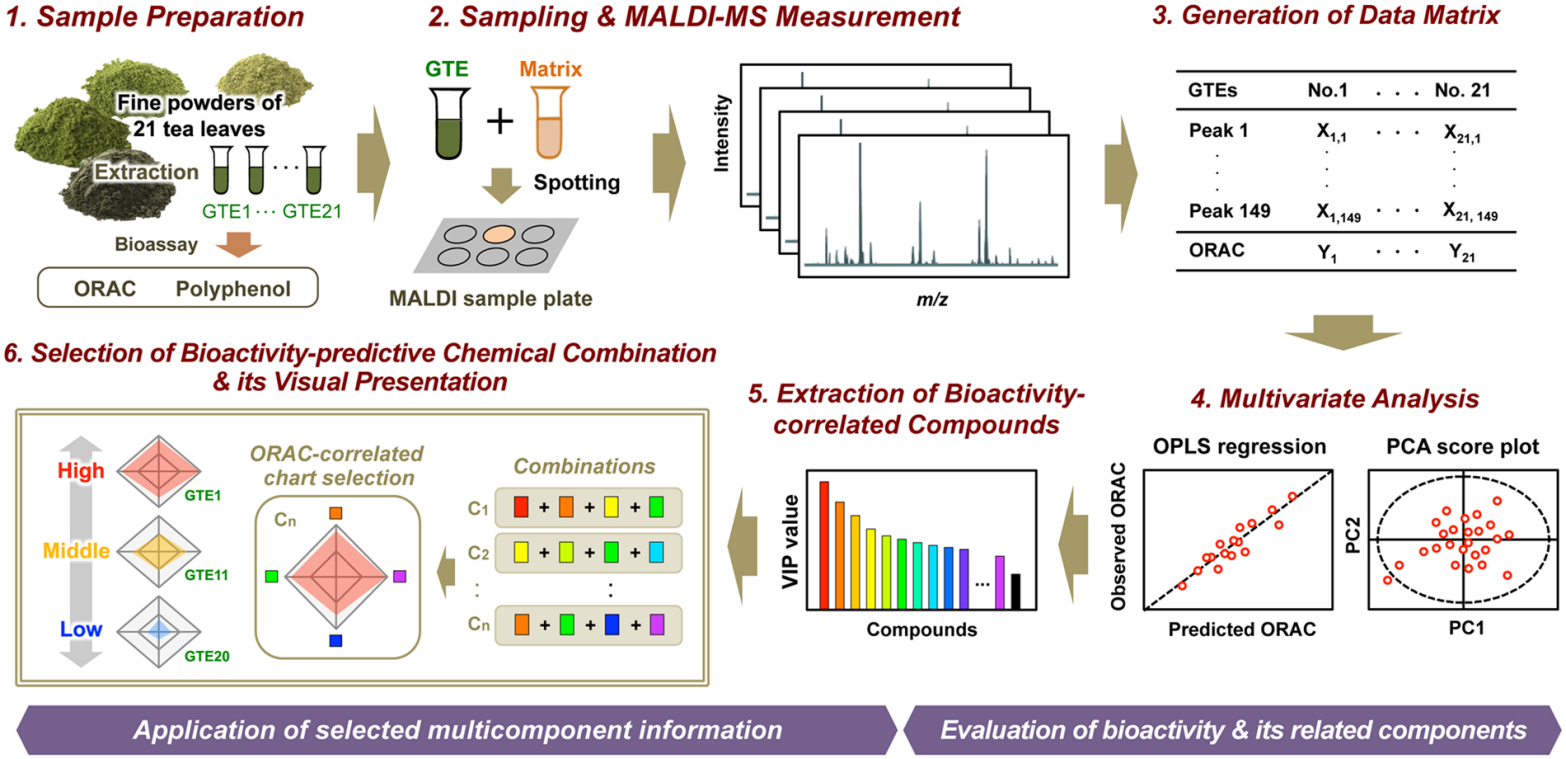
Experimental design for chemometrics-based evaluation of bioactivity of 21 GTE panels representing multicomponent systems.

**Figure 2:**
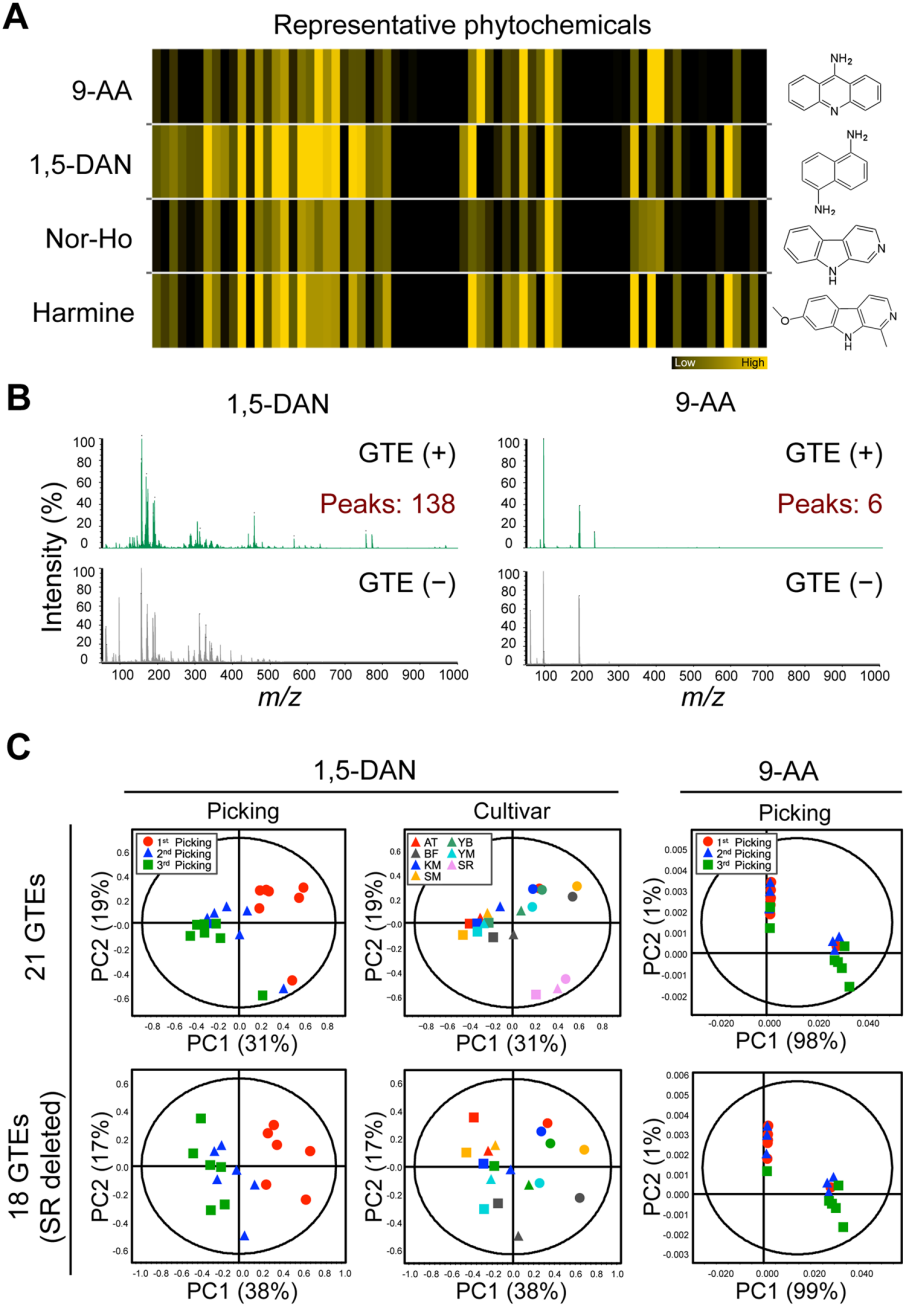
Metabolic profiling-based evaluation of GTE quality using MALDI–MS system with matrix screening. (**A**) Heatmap analysis showing the different ionization rates of the 72 phytochemicals by the 4 matrices, with the chemical structures of the matrices shown on the right. (**B**) Mass spectra for GTE–matrix mixtures (upper panels) or isolated matrices (lower panels) (peak heights represent the relative signal intensities, where the intensity of the strongest peak is 100%). The total number of peaks detected by each matrix is shown. (**C**) PCA score plot showing different clusters of MS profiles, based on the attributes of picking seasons and cultivars.

Herein, we aimed to develop a MALDI–MS system suitable for the chemometrics-driven evaluation of the bioactivity of diverse GTE panels (Fig. 1). In MALDI–MS, the matrix preparation, including the selection of the matrix and solvent, is a critical step to ensure efficient ionization of the analyte, because the detection of the analyte is completely dependent on these conditions^11^. Because little is known about which matrices can simultaneously ionize multiple compounds from herbal extracts, including various phytochemicals, we first screened the optimum matrix for detecting approximately 70 representative phytochemicals (Supplementary Table S1) among four potentially high-performing matrices previously reported^7, 12, 13^. A solution of each matrix in 100% methanol (MeOH) or acetone was mixed with an equal volume of a phytochemical solution. This mixture was spotted onto a stainless steel MALDI sample plate and analyzed by MALDI-TOF-MS in the negative ionization mode. All the resulting spectral data were processed using an in-house script (Supplementary Fig. S1) that simultaneously detects authentic phytochemical peaks in their basic, deprotonated ion form [M–H]^−^ while effectively excluding various matrix background peaks. The obtained information on the intensity of all the peaks was converted to a heatmap to visually compare the extent of each ionization (Fig. 2A and Supplementary Table S1). The detectability of the phytochemicals was differed among the four matrices, namely 9-AA, 1,5-diaminonaphthalene (1,5-DAN), norharmane (nor-Ho), and harmine. Based on the ionization rate and the number of phytochemicals successfully ionized, the best ionization performance was achieved by 1,5-DAN, and the acetone (Fig. 2A) provide a better solvent than MeOH (Supplementary Fig. S2). The ionization efficiency of the phytochemicals varied widely depending on their chemical structures (Supplementary Fig. S3). Furthermore, 1,5-DAN obtained the greatest number of peaks (138 following background subtraction) from the crude aqueous GTEs, many more than were detected by 9-AA (Fig. 2B). In fact, while 9-AA is the most commonly used matrix in non-targeted metabolomic analysis^6, 8^, it showed the worst detection performance in this study (Fig. 2A and Supplementary Fig. S2). To examine the potential of 1,5-DAN as a matrix for the acquisition of the chemical compositions and the subsequent quality evaluation of diverse GTEs, MALDI–MS measurements and the subsequent multivariate statistical analysis were performed using 21 distinct GTEs from 7 representative Japanese green tea cultivars (*Camellia sinensis* L. and *C. sinensis* x *C. taliensis*) cropped during 3 different picking seasons (Table 1 and Supplementary Table S2). The score plot of the principal component analysis (PCA), an unsupervised multivariate statistical analysis, showed clear clusters, one consisting of the Sunrouge (SR) cultivar (*C. sinensis* x *C. taliensis*), and the other consisting of the remaining cultivars (*Camellia sinensis* L.) (Fig. 2C). The cluster separation of the cultivars was observed along the PC2 axis (1,5-DAN, right panel). Regardless of whether the SR cultivar was included or excluded (leaving 21 or 18 GTEs, respectively), clusters related to the picking season could be observed along PC1 (1,5-DAN, left panel). These results strongly suggest that the compositional differences among the GTEs can account for the different cultivars and picking seasons. In contrast, no such cluster formation was observed in the MALDI–MS-MP results when using 9-AA as a matrix (Fig. 2C, right panel). These results show that phytochemical-based matrix screening is an effective strategy for selecting the optimal matrix, in this case 1,5-DAN, for the analysis of the chemical compositions of diverse GTEs and their quality evaluation.

**Table 1:** Ranking of anti-oxidant activity of 21 GTEs, consisting of 7 distinct cultivars harvested at 3 different picking seasons.

Name	Cultivar	Picking season	ORAC Rank	ORAC (μM TE/L)	Polyphenol (mg GAE/mL)
AT1	Asatsuyu	1st	19	35,647	1.93
AT2	Asatsuyu	2nd	15	38,661	2.11
AT3	Asatsuyu	3rd	3	51,482	2.54
BF1	Benifuki	1st	10	42,552	2.04
**BF2**	**Benifuki**	**2nd**	**1**	**65,085**	**2.56**
BF3	Benifuki	3rd	2	55,612	2.54
KM1	Kanayamidori	1st	7	44,400	2.00
KM2	Kanayamidori	2nd	9	43,107	2.11
KM3	Kanayamidori	3rd	4	48,544	2.41
**SM1**	**Saemidori**	**1st**	**21**	**25,445**	**1.96**
SM2	Saemidori	2nd	13	40,191	2.13
SM3	Saemidori	3rd	6	46,160	2.33
SR1	Sunrouge	1st	16	37,930	2.44
SR2	Sunrouge	2nd	11	42,436	2.33
SR3	Sunrouge	3rd	14	38,953	2.17
YB1	Yabukita	1st	20	34,246	2.12
YB2	Yabukita	2nd	18	36,545	2.13
**YB3**	**Yabukita**	**3rd**	**12**	**41,729**	**2.34**
YM1	Yutakamidori	1st	17	37,746	2.21
YM2	Yutakamidori	2nd	8	43,496	2.28
YM3	Yutakamidori	3rd	5	47,566	2.65

Bold letters indicate three representative GTEs, corresponding to the highest, the middle or the lowest rank of ORAC values.

### Applicability of 1,5-DAN-based MALDI–MS-MP for bioactivity evaluation of diverse GTEs with different antioxidant activity

**Figure 3:**
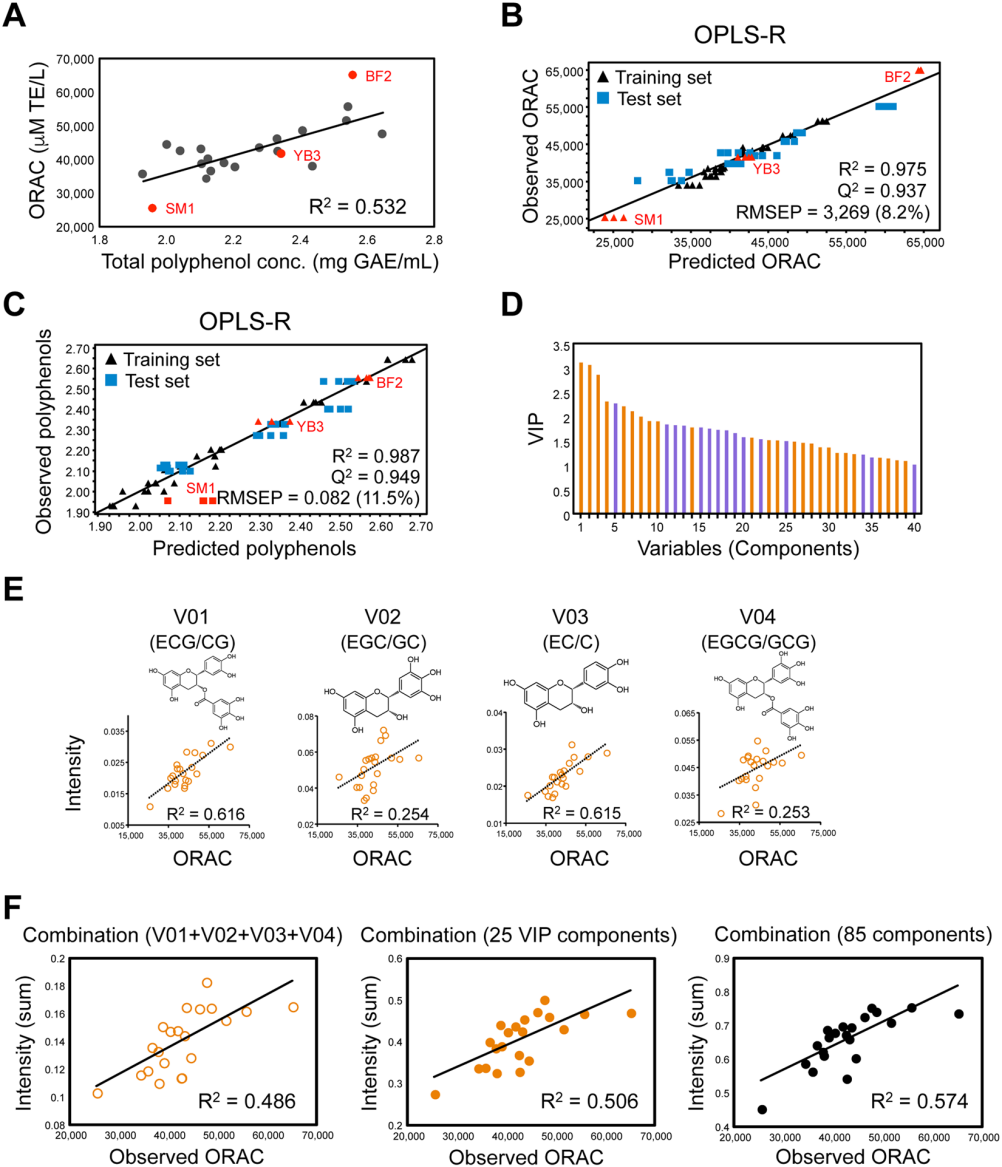
Construction of bioactivity-prediction model to predict the anti-oxidant activity of GTEs based on their composition profiles. (**A**) Correlation between ORAC and total polyphenol content. ORAC values are presented as Trolox equivalents (TE). Total polyphenol contents are presented as gallic acid equivalents (GAE). Models for predicting (**B**) ORAC or (**C**) total polyphenol content were calculated from the MALDI–MS datasets of 21 GTEs, including 13 training (black triangles) and 8 test (blue squares) sets. (**D**) Bar chart showing the influence of variables used to create the ORAC-prediction model for GTEs (Y-axis is the value of variable-importance-in-projection, VIP). Forty variables with large VIP values (>1) were extracted. Orange bars indicate positive correlations between the intensity of the component and ORAC. Purple bars indicate negative correlations. (**E**) Correlations between ORAC and the intensity of each of the top-4 components with the largest VIP values (>1). (**F**) Correlations between ORAC and the summed abundances of multiple components. Left panel: combination of the above-mentioned top-4-VIP components. Middle panel: combination of the 25 positively correlated components with the largest VIP values (>1). Right panel: combination of all 85 positively correlated components from among the 149 total components.

To determine whether the bioactivity of diverse GTE panels could be evaluated by 1,5-DAN-based MALDI–MS-MP, we measured their oxygen radical absorbance capacity (ORAC) values. This form of antioxidant activity is known as one of the major bioactivities of GTEs^9, 14, 15^. The ORAC values were found to clearly differ among the 21 GTEs (Table 1). Generally, polyphenols are known to be among the bioactive factors with the highest ORAC^15^. Herein, we analyzed the relationship between the ORAC values and the total polyphenol contents of the 21 GTEs (Fig. 3A). A positive correlation was observed, and the coefficient of determination was 0.532. This value suggests that the observed polyphenol information alone is insufficient for effectively explaining ORAC values of GTEs, and information on the other components present in GTEs is required. In contrast, using orthogonal partial least-squares (OPLS) regression analysis (a form of supervised multivariate statistical analysis), a reliable bioactivity-prediction model to predict the ORAC of diverse GTE panels was constructed on the basis of their composition profiles obtained from the 1,5-DAN-MALDI–MS measurements. For this model, the goodness-of-fit parameter R^2^ = 0.975 (corresponding to the coefficient of determination), the goodness-of-prediction parameter Q^2^ = 0.937, and the root mean squared error of the prediction (RMSEP) = 8.2% (Fig. 3B and Supplementary Table S2). Furthermore, information on the composition profiles was also found to be sufficient for a predictive evaluation of the total polyphenol contents (Fig. 3C). These results suggest that MALDI–MS can be used to obtain multivariate information on GTEs, namely their compositional balance, which may serve as a more effective indicator for explaining (either predicting or discriminating) their bioactivity (namely ORAC) and the related property, total polyphenol content.

In this model, the compounds with the highest predictive power for ORAC were also distinguished by their variable-importance-in-projection (VIP) values. The 40 component peaks with large VIP values (>1) corresponded to the compounds that were most predictive for bioactivity (Fig. 3D and Supplementary Table S3). Among these component peaks, representative relationships between the top-4 peak intensities and the observed ORAC values in all GTEs are shown in Fig. 3E. These 4 components, labelled V01 to V04, are major green tea catechins, (−)-epicatechin (EC), (−)-epigallocatechin (EGC), (−)-epicatechin-3-*O*-gallate (ECG), and (−)-epigallocatechin-3-*O*-gallate (EGCG), which were all included in the phytochemical library used in the matrix screening (Fig. 2A and Supplementary Fig. S2). All four components were detected simultaneously with their epimerized forms, (−)-catechin (C), (−)-gallocatechin (GC), (−)-catechin-3-*O*-gallate (CG), and (−)-gallocatechin-3-*O*-gallate (GCG), respectively, which were newly generated by the hot-water extraction of the tea leaves. The best correlation between intensity and ORAC was observed for the V01 component (corresponding to ECG/CG), but the coefficient of determination was nonetheless relatively low (failing to satisfy R^2^ > 0.7). The combined peak intensity of all 4 compounds, i.e. V01 to V04, proved to be less strongly correlated with ORAC than the intensity of V01 alone (Fig. 3F, left panel). This tendency was also observed in the concentration data for these compounds (Supplementary Fig. S4). Furthermore, a combination of 25 components (all having a positive correlation with ORAC and a large VIP value (>1)), as well as the combination of all 85 positively correlated components, also showed relatively low correlations (failing to satisfy R^2^ > 0.7) (Fig. 3F, middle and right panels). These results suggest that while there is a potential relationship between MALDI–MS datasets and ORAC values, the absolute abundance of each component with a large VIP value (>1) or the simple summation thereof are not always effective for explaining the ORAC of diverse GTEs. In contrast to this partial component information, the comprehensive compositional balance, i.e. the relative abundance of all GTE-derived components, can be successfully used in OPLS regression to evaluate the bioactivity of GTEs. Taken together, these findings indicate that 1,5-DAN-MALDI–MS-MP is a viable strategy for the bioactive evaluation of diverse sample panels representing crude multicomponent systems.

### Strategy for effectively selecting candidates for bioactive chemical combinations from bioactivity-predicting model data

**Figure 4:**
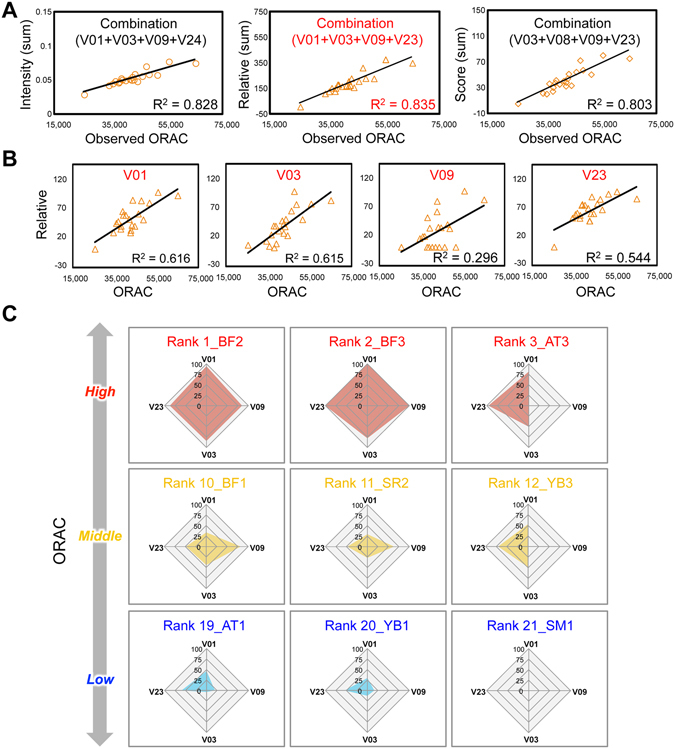
Chemometrics-driven selection of bioactivity-correlated chemical combination in GTEs and visualization of observed ORAC values using the selected combination. (**A**) The highest correlation was found between observed ORAC value and the summed abundance (Intensity) of 4 components as a bioactivity-predictive combination. Correlations based on the relative value (Relative; Maximum: 100, Minimum: 1) and ranked scored value (Score; Top: 21, Bottom: 1) of the summed abundance are also shown. (**B**) Correlation of Relative value of each individual component with ORAC. (**C**) Observed ORAC values of GTEs visualized as radar charts using information from the 4 selected components. Selected representative charts of the GTEs are shown, demonstrating that ORAC can be visually estimated from the 4 component abundances.

Compared with the absolute abundance of a single component, the relative abundance of all coexisting multicomponent factors was found to be more predictive and discriminative information for the bioactivity evaluation of GTEs. In most cases, evaluation of the attributes of crude samples in research on pharmaceuticals, nutraceuticals, and functional foods has focused on the absolute abundance of a single component as a biomarker^1^, while the importance of multicomponent information has mostly been neglected. In addition to the chemometric approach for applying such information to the evaluation and understanding of physiological activity, the development of a methodology for effectively selecting a meaningful combination of components in crude sample systems, on a sound theoretical basis, remains a challenge. To further examine the utility of MALDI–MS-derived multivariate data, herein, we attempted to use these data to select bioactivity-predictive combinations of components. Firstly, we screened various combinations of 4 components, in which each component was drawn from those with large VIP values (>1), in terms of their contribution to the construction of an ORAC-prediction model (Supplementary Table S3). Since there were 40 eligible components (i.e. V01–V40), each 4-component combination represented 10% of this group. For each combination, three correlation measures were calculated, namely the summed intensity of the 4 compounds (Intensity), the relative value of the summed intensity (Relative), and its ranked score (Score), and the correlations of each measure with the ORAC values were investigated. Among the various combinations (Supplementary Table S4), those exhibiting the highest correlations with ORAC are shown in Fig. 4A. Although the correlation of the combination of components with the top-4 VIP values (V01, V02, V03, and V04) was low (Intensity: R^2^ = 0.486, Relative: R^2^ = 0.561, Score: R^2^ = 0.510), combinations of the other four components (Intensity: V01 + V03 + V09 + V24, Relative: V01 + V03 + V09 + V23, Score: V03 + V08 + V09 + V23) showed a much higher correlation value (Intensity: R^2^ = 0.828, Relative: R^2^ = 0.835, Score: R^2^ = 0.803). Among the three measures, the Relative value thus showed the best correlation. Furthermore, this combination showed a higher correlation than any of its individual components (Fig. 4B, Relative data; Supplementary Table S4, Intensity and Score data). These results suggest that the summed abundance of 4 carefully selected components (Relative: V01 + V03 + V09 + V23) can be more predictive and discriminative information for the bioactive evaluation of diverse GTE panels than the abundance of any single component.

Next, we attempted to create an ORAC-correlated chart using this multicomponent information to help readers to intuitively and easily understand these complex chemometric data. To visually express the combinational information contained in Fig. 4A, we constructed a radar chart reflecting the abundance of each component. A representative section of this ORAC-correlative chart, using the Relative data of the combination (V01 + V03 + V09 + V23) for nine GTEs, is illustrated in Fig. 4C (the charts for all GTEs and both Intensity and Score data are shown in Supplementary Fig. S5). This radar chart visually confirms the relationship between the intensities and the observed ORAC values. In contrast, no such close relationship was observed for the combination (V01 + V02 + V03 + V04), which had a low correlation value (Supplementary Fig. S6). These results suggest that we can intuitively but rationally assess the ORAC values of diverse GTE panels through post-processing the compositional information extracted from OPLS regression analysis (Supplementary Fig. S7). In summary, we have successfully developed a chemometric methodology capable of rationally selecting, by intuitive visualisation, an ORAC-correlated chemical combination from MALDI–MS-derived multivariate data of diverse bioactive GTE panels.

## Discussion

In this study, we demonstrated for the first time that the MALDI–MS-MP technique could be used to evaluate the antioxidant activity of diverse GTE panels based on their compositional balance, and select an effective chemical combination able to predict the bioactivity. In pharmaceutical, nutraceutical, and food functionality research, the conventional evaluation method for bioactivity, targeted analysis, attempts to predict the total bioactivity of entire samples by measuring the activity and abundance of a single component. However, this approach carries a risk of overestimating or underestimating bioactivity by neglecting the potential interfering effects of multiple coexisting factors. In addition, such an approach cannot easily calculate the relative contribution of all coexisting factors to the total bioactivity of entire samples. Furthermore, the screening of bioactivity-related chemical combinations from crude samples is generally time-consuming, expensive, and labour-intensive due to multiple, repetitive processes of fractionation and bioassay. The results of this study suggested that our chemometrics-based and non-targeted MP approach, using multiple GTE panels with diverse bioactivity, was able to overcome these drawbacks (Supplementary Fig. S8).

Unlike the traditional chromatography-coupled MS platforms (LC–MS and GC–MS) used frequently in MP research, it is expected that MALDI–MS, a direct analytical system, may allow a more rapid, high-throughput MP of the largest possible number of samples^5^. There have been several reports of the application of MALDI–MS to protein and peptide profiling for the quality evaluation of crude food samples, such as beers^16^, milk, hazelnut^17^, and peanut^18^, but metabolomic applications have proven more challenging. The choice of matrix is one of the most important issues affecting the non-targeted metabolomic analysis of crude extracts. Although several matrices have been reported for the targeted analysis of a single specific component^19^, little is known about which matrices can simultaneously ionize various low-molecular-weight components from crude GTE samples. To our knowledge, the present study was the first to screen matrices to identify the optimum matrix for ionizing the 72 representative phytochemicals at a reliable standard level. As shown in Supplementary Fig. S3, the ionization efficiency of the phytochemicals varied widely depending on their chemical structures. 1,5-DAN-based MALDI–MS system enabled to preferably ionize flavonone, flavonol, flavan-3-ol, and isoflavone. Intriguingly, their ionization efficiency was strongly dependent on both the position and number of hydroxyl group. In GTE samples, flavan-3-ols, catechins, were highly detected as ORAC-correlated compounds with the highest VIP values (>1). On the other hand, zero or low detectability was observed in anthocyanidin, carotenoids, disaccharides, steroidal alkaloids, phytosterol, oxylipin, and aryl isothiocyanate. It was known that these compounds ware partially detected in the positive ionization mode using other matrices. The present 1,5-DAN-MALDI–MS system is negative ionization mode, and combination of both ionization modes may lead to further improvement of detectability. At least, this matrix screening proved an effective strategy for detecting GTE-derived compounds at the crude sample level, and the MS approach based on their composition profiles enabled us to evaluate their quality and bioactivity. Although 1,5-DAN enabled to detect more number of compounds compared to other matrices tested, there were still many non-detected compounds. Thus, we cannot exclude the involvement of such components in ORAC activity at present. The improvement of detectability of MALDI–MS is indispensable for successfully constructing more precise and accurate predictive system. The screening or development of new matrices to more effectively ionize various phytochemicals is required. We believe that basic information on the potential relationship between the ionization efficacy and chemical structures of phytochemicals (Fig. 2A, Supplementary Fig. S2, and Supplementary Fig. S3) may be useful for the MALDI–MS-MP of phytochemical-containing crude samples and for the effective screening and development of matrices.

Previously, LC–MS and GC–MS have been the techniques most commonly used for the MP of medicinal herbs, agricultural products, and foods for quality evaluation. Previously, we showed for the first time the usefulness of LC–MS-MP for evaluating the apoptosis-inducing activity of GTEs and for screening anti-cancer compounds or synergetic sensitizers^20^. In this study, we also performed conventional LC–MS-MP for the same GTE samples used in the main experiment (Supplementary Fig. S9 and Supplementary Table 5S). LC–MS detected a greater number of peaks (507) than did MALDI–MS (149), and MP based on the composition profiles from LC–MS successfully achieved various quality evaluations, such as distinguishing the similarity and dissimilarity among cultivars and picking seasons (Supplementary Fig. S9A), and the prediction of ORAC and polyphenol content (Supplementary Fig. S9B,C). Interestingly, however, MP based on the MALDI–MS datasets (Fig. 3B) showed a better performance than that based on LC–MS. As shown in Supplementary Fig. S10, LC–MS data was not able to construct appropriate OPLS models using datasets excluding components with higher VIP values (>1) (Supplementary Table S6). In contrast, MALDI–MS data was not greatly affected by the exclusion of such components (Supplementary Tables S3 and S7) although its predictive performance was slightly lowered. These results suggest that MALDI–MS datasets include more number of peaks correlated with ORAC values or polyphenol contents compared to LC–MS datasets, and such peaks may coordinately contribute to the better performance of MALDI–MS datasets. The lowered performance of LC–MS datasets may be due to the low-correlative ability (predictability) of components with VIP values (<1) to ORAC values or polyphenol contents. These findings endorse the quality of the composition profiles obtained from MALDI–MS measurements and their applicability to the rapid and simple non-targeted MP of medicinal herbs, agricultural products, and foods.

Although it is known that the polyphenol content is correlated with ORAC^15, 21^, as shown in Fig. 3A and B,information on the metabolomic composition profile proved to be a better explanatory variable for ORAC than the total polyphenol content was. Similarly, the metabolomic composition was also found to be a better explanatory variable for the polyphenol content than ORAC was (Fig. 3A and C). These results suggest that a chemometrics approach based on OPLS regression analysis can provide an evaluation index that more accurately predicts bioactivity and its related properties. In contrast to indices corresponding to the “summed abundance” of multiple coexisting factors, such as total polyphenol content, their “relative abundance”, i.e. compositional balance, may serve as a functional unit for discriminating or predicting their bioactivity. The OPLS regression analysis identified the ORAC-contributing components based on their VIP values, but the correlation between the abundance of a single component and ORAC was relatively low even for the top-4 VIP components (V01 + V02 + V03 + V04; Fig. 3E and Supplementary Table S3). In contrast, combinations of 4 components (Intensity: V01 + V03 + V09 + V24, Relative: V01 + V03 + V09 + V23, Score: V03 + V08 + V09 + V23) carefully selected from the 40 components with VIP > 1 showed much improved abundance–ORAC correlation values (Fig. 4A). Among the top-25 VIP-valued components (VIP > 1) positively correlated with ORAC, the number of combinations of 2–4 components was 15,250, and the combinations of 4 components showed a better correlative performance than those of 2 or 3 components (Supplementary Table S4). These results indicate that this combination-selecting procedure is an effective and straightforward methodology for more accurately evaluating the ORAC using a few chosen components. This finding suggests a promising strategy for efficiently selecting candidate combinations from multivariate data of multiple sample panels with diverse bioactivity, which is important but technically challenging in pharmaceutical, nutraceutical, and food functionality research, where single-sample panels are dominant. In conventional research on the evaluation of quality and bioactivity, the goal is usually to isolate a single component from crude mixtures and use its abundance as a basis for predicting the sample’s properties. In contrast, our study has shown that bioactivity can be predicted using multicomponent information, i.e. the abundance of a combination of components, the accuracy of which depends on the chosen combination of components and the choice between three abundance measurements (Intensity, Relative, or Score) (Fig. 4A and Supplementary Table S4). These efforts will contribute to an enhanced understanding of chemometrics procedure and an effective and simple means of data presentation using multicomponent information, and such information may benefit the application of multivariate statistical methodology to the bioactivity evaluation of crude multicomponent systems.

Although the chart visualization is effective strategy to easily understand ORAC values using the selected component combination, this approach includes the possibility of causing the difference between chart appearance and bioactivity. In the Relative data of the selected combination (V01 + V03 + V09 + V23) (Fig. 4C), the filled area of the SM1 chart was not observed in appearance, but it was clearly observed in the Intensity data of the combination (V01 + V03 + V09 + V24) (Supplementary Fig. S5B). Both V01 and V03 in the Intensity data were certainly present in SM1, indicating that these components contributed to the ORAC activity of SM1. In the Relative data, the maximum intensity is 100, and the minimum intensity is 1. The value of V01 intensity was converted into 1 as the Relative value. This minimum value was also applied in V09 and V23. Because V01, V09, or V23 was very small value, the area appearance became close to zero in a diamond-like radar chart, even though V03 was present as the value of 5.50. This is one of the causes of the difference between the chart appearance and ORAC activity. Except for such 4 components, 36 other components with the highest VIP values (>1) contribute to the ORAC activity. Thus, radar chart, using more than 5 components, may resolve such an issue, and will lead to the construction of much better model effectively improving the difference between chart appearance and bioactivity.

Previously, we have reported the performance of basic MALDI–MS procedures, including the repeatability, sensitivity, and linearity of detection of low-molecular-weight metabolites^6–8, 22^. Here we attempted to apply this established technique to crude herbal extracts. The appropriate linearity was obtained for the representative GTE components (EGCG, EGC, ECG, and EC) at the standard level (Supplementary Fig. S11A). These compounds were detected in a dilution series of the representative GTE (YB1) (Supplementary Fig. S11B). A 10-fold dilution of the GTE stock solution was used in all experiments under the condition ensuring the linearity of four compounds. MS data of the representative GTE (YB1) sample were acquired on a different day (Supplementary Fig. S11C), and there were no significant changes in relative spectral patterns. We were also able to stably construct preferable OPLS models using such MS data (Supplementary Fig. S11D). These results ensure the robustness of our proposed MALDI–MS-MP system. The present technique is effective for performing the high-throughput first screening of bioactive compounds and combinations from crude herbal samples at the initial step of research. However, this MS technique is still *in vitro* cell-free system, and further its technical validity would be reinforced through some sort of comparison with *in vitro* cellular systems and/or *in vivo* systems. In our research groups, some MALDI–MS-MP researches, using other bioactivities and medicinal herbal extracts, bacterial samples, and animal/human body fluids, are in progress. These attempts may also contribute to the evaluation of technical validity and further improvement of our proposed MS technique.

In summary, we have established an effective strategy for evaluating a measure of the bioactivity, namely the antioxidant activity, of diverse GTE panels by non-targeted MP-based chemometric analysis using a high-throughput analytical system, MALDI–MS. This approach also enabled us to easily extract a bioactivity-predictive chemical combination from multicomponent information. Our proposed MALDI–MS procedures (sample preparation, matrix selection, peak detection and alignment, and multivariate statistical analysis) contribute to the construction of standardization of crude herbal extracts for successfully performing MP and the screening of chemical combination. These results allowed us to overcome the drawbacks of the conventional MP technique (Supplementary Fig. S8), and may enable a rapid and simple high-throughput MP of crude samples for the evaluation of their bioactivity (Supplementary Fig. S12) as well as various applications, including the quality assessment, breeding, screening, and monitoring of low-molecular-weight chemicals. In addition, further chemometric research will open new avenues for investigating the potential relationship between the bioactivity of crude extracts and their multiple coexisting factors, and for determining effective chemical combinations for bioactivity prediction. This may contribute to the discovery of new scientific data helpful for the development of multicomponent botanical drugs and dietary supplements, herbal medicines, functional foods, and combinations of foods/beverages optimized to promote health and reduce the risk of disease.

## Methods

### Chemicals

All chemicals used were of analytical reagent grade. Matrix chemicals and solvents were purchased from Wako Pure Chemical Industries, Ltd (Osaka, Japan), Tokyo Chemical Industry Co., Ltd (Tokyo, Japan), or Sigma-Aldrich (St Louis, MO, USA) if not stated otherwise. All catechins were purchased from Nagara Science Co., Ltd (Gifu, Japan). Folin–Ciocalteu reagent, sodium carbonate, and 9-aminoacridine (9-AA) were obtained from Merck (Darmstadt, Germany). 9-AA was recrystallized prior to use.

### Preparation of GTEs

We prepared 21 distinct hot-water extracts from the leaves (Table 1) of 7 Japanese green tea cultivars (*Camellia sinensis* L. and *C. sinensis* x *C. taliensis*), which were cropped during 3 different picking seasons (7 April–7 May, 28 May–11 June, and 4–17 July). Six cultivars (*Camellia sinensis* L.) were purchased from retail tea stores, and one cultivar (*C. sinensis* x *C. taliensis*), Sunrouge (SR), was kindly donated by Nippon Paper Industries Co., Ltd (Tokyo, Japan). A fine powder of the dried leaf (30 mg) from each cultivar was added to 1.5 mL boiling water for 10 min. The extract was centrifuged at 15,000 × *g* for 10 min, and the supernatant was subjected to further analyses.

### MALDI–MS analysis

Individual phytochemical standard compounds were dissolved in water or MeOH, diluted to give graded concentrations (100 ppm), and mixed with a matrix (9-AA, 1,5-DAN, Nor-Ho, or harmine)/100% MeOH or acetone solution (10 mg/mL) at a ratio of 1:1 (v/v). The sample (0.5 μL) was spotted onto the ground-steel MALDI plate and air-dried. Four spots were deposited from each individual sample and their data averaged for the subsequent data analyses. A MALDI-TOF-MS (AXIMA Performance, Shimadzu, Japan) was used for all the analyses. Each mass spectrum was acquired with 5 laser shots. For each sample spot, 121 spectra were mean-centered. All spectrometric data were processed and analysed using the Shimadzu Biotech Launchpad software. The ionization of each phytochemical was confirmed by a deprotonated ion peak [M–H]^−^. In the GTE experiments, GTE samples (1/10 dilution in water) were subjected to MALDI–MS analysis using 1,5-DAN or 9-AA as the matrix under the same spotting and measuring conditions as for the phytochemicals. Then, peak picking and alignment, noise reduction, and deisotoping of the obtained spectral data were performed using an in-house script (Supplementary Fig. 1S), and the resultant data were subjected to multivariate statistical analysis. Phytochemical peaks were assigned by MS/MS analysis or by searching for their precise masses using the MassBank metabolite databases.

### Multivariate statistical analysis

The datasets of the 21 GTEs were subjected to multivariate statistical analysis to identify similarity/dissimilarity among the samples (149 (1,5-DAN) or 18 (9-AA) distinct *m*/*z* peaks). We conducted an unsupervised multivariate principle component analysis (PCA) and a supervised multivariate OPLS analysis using SIMCA-P+ ver.12 (Umetrics, Umea, Sweden). PCA models are depicted as score plots and consist of two synthetic variables: principal component (PC) 1 (accounting for the greatest proportion of the total variance) and PC2 (accounting for the second greatest proportion of the total variance orthogonal to PC1). These plots display intrinsic groups of samples based on their spectral variations. This analysis attempts to explain the original features of the samples as far as possible based on a ratio of the sum of the percentages of PC1 and PC2.

OPLS regression analysis, which can be described as the regression extension of PCA, was chosen to create the bioactivity-prediction model using SIMCA-P+. OPLS derives latent variables that maximize the covariation between the measured metabolite data and the response variable (ORAC). This differs from PCA, which utilizes the maximum variation in the metabolite data matrix. The quality of the OPLS model was evaluated by the goodness-of-fit parameter R^2^ and the predictive capacity parameter Q^2^, with values higher than 0.5 indicating good quality.

A heatmap was generated using the statistical package Multi-Experiment Viewer (MeV v4.9) (http://www.tm4.org/mev/). This summarises the Z-scores of the peaks of the 72 phytochemicals, showing differences in ionisability among the matrices.

### ORAC assay

The ORAC assays of GTE samples (1/1000 dilution in phosphate buffer) were performed as detailed by Ou *et al*.^14^ using an automated Microplate Reader SH-9000 (Corona Electric Co., Ltd, Hitachinaka, Japan). Analyses were conducted in pH 7.0 phosphate buffer at 37 °C. Peroxyl radicals were generated using 2,2′-azobis(2-amidinopropane) dihydrochloride, and fluorescein was used as the substrate. The fluorescence conditions were as follows: excitation, 485 nm; emission, 520 nm. Analyses were conducted in duplicate (n = 6). ORAC values were calculated as Trolox (6-hydroxy-2,5,7,8-tetramethylchroman-2-carboxylic acid) equivalents (TE).

### Total polyphenol assay

The total soluble polyphenols in the GTEs (1/100 dilution in water) were determined with Folin–Ciocalteu reagent according to the method of Slinkard and Singleton^23^ using gallic acid as a standard. Analyses were conducted in duplicates (n = 3). Results were expressed as gallic acid equivalents (GAE).

## Electronic supplementary material

Supplementary Information
