# Supplementary material for: A Chemometrics-driven Strategy for the Bioactivity Evaluation of Complex Multicomponent Systems and the Effective Selection of Bioactivity-predictive Chemical Combinations
Source: Sci Rep. 2017 May 23;7:2257. doi: 10.1038/s41598-017-02499-1 (PMC5442154; doi:10.1038/s41598-017-02499-1)
Supplement: Supplementary file 1 — Supplementary Information [file 41598_2017_2499_MOESM1_ESM.pdf]

A Chemometrics-driven Strategy for the Bioactivity Evaluation of Complex Multicomponent Systems and the Effective Selection of Bioactivity-predictive Chemical Combinations

Yoshinori Fujimura[1,#,*], Chihiro Kawano[1,#], Ayaka Maeda-Murayama[1], Asako Nakamura[1], Akiko Koike-Miki[1], Daichi Yukihira[1], Eisuke Hayakawa[2], Takanori Ishii[1], Hirofumi Tachibana[1,3], Hiroyuki Wariishi[1,3,4], and Daisuke Miura[1,*]

[1]Innovation Center for Medical Redox Navigation, Kyushu University, 3-1-1 Maidashi, Higashi-ku, Fukuoka 812-8582, Japan; [2]Okinawa Institute of Science and Technology Graduate University, 1919-1 Tancha, Onna-son, Kunigami-gun, Okinawa 904-0495, Japan; [3]Faculty of Agriculture and [4]Faculty of Arts and Science, Kyushu University, 6-10-1 Hakozaki, Higashi-ku, Fukuoka 812-8581, Japan.

#These authors equally contributed to this work.

*Correspondence and requests for materials should be addressed to Y. F. (email: fujimurayoshinori@gmail.com) or D.M. (email: daipon@agr.kyushu-u.ac.jp)

The authors declare no conflict of interest.

Contents:

Supplementary methods

LC–MS analysis

Supplementary Figures and Tables

Supplementary Figure S1. The schematic flow of the peak picking and alignment of non-targeted MALDI–MS data.

Supplementary Figure S2. Heatmap representation of the ionization of the representative phytochemicals by MALDI–MS using four matrices dissolved in MeOH solution.

Supplementary Figure S3. The representative relationship between the chemical structure and the ionization efficiency of phytochemicals.

Supplementary Figure S4. The correlation plot of the highest VIP-valued compounds at the concentration level of GTEs.

Supplementary Figure S5. The chart visualization of the observed ORAC values of GTEs using the selected combinations with the highest correlation values.

Supplementary Figure S6. The chart visualization of the observed ORAC values of GTEs using the selected combination with low correlation value.

Supplementary Figure S7. Scheme of chemometrics-driven selection of GTE bioactivity-correlated chemical combinations.

Supplementary Figure S8. Comparison of the MALDI–MS-MP system with the conventional strategy in view of 2 distinct research fields.

Supplementary Figure S9. LC–MS-MP of 21 GTEs for evaluating their quality and bioactivity.

Supplementary Figure S10. The effect of components with the highest VIP values (>1) on the construction of OPLS regression models.

Supplementary Figure S11. The evaluation of the robustness of the present MALDI–MS-MP system.

Supplementary Figure S12. Graphical representation of MALDI–MS-MP system for the bioactivity evaluation of diverse bioactive sample panels representing multicomponent systems and the effective selection of bioactivity-predictive chemical combinations.

Supplementary Table S1. The ionization rates of the representative phytochemicals for MALDI matrix screening.

Supplementary Table S2. MALDI–MS datasets of 21 GTEs for multivariate statistical analyses.

Supplementary Table S3. The top-40 VIP-valued components highly contributing to the construction of OPLS regression model and their correlations with ORAC values.

Supplementary Table S4. Combinations of multiple components with the highest VIP values (>1) and their correlation values using three abundance measures.

Supplementary Table S5. LC–MS datasets of 21 GTEs for multivariate statistical analyses.

Supplementary Table S6. Selected peaks with the highest VIP values (>1) contributing to the construction of OPLS regression models in LC–MS datasets.

Supplementary Table S7. Selected peaks with the highest VIP values (>1) contributing to the construction of the polyphenol-predictive OPLS regression model in MALDI–MS datasets.

Supplementary methods

LC–MS analysis. All GTEs were subjected to LC–MS analysis using a LCMS-IT-TOF instrument (Shimadzu, Kyoto, Japan). The instrument was fitted with a Luna C18(2) column (250 mm × 1.0 mm, 5 μm particle size, Phenomenex, Torrance, CA) maintained at 40°C. The mobile phase solvents were 0.1% aqueous formic acid (solvent A) and methanol (solvent B). Solvent B was 5% for 2.0 min, increased linearly from 5% to 60% over 6.0 min, and further increased from 60% to 100% at 13.0 min, and 100% holding for 4.5 min. Flow rate was constant (0.1 mL/min). The MS instrument was operated using an ESI source in both positive and negative ionization modes with survey scans acquired from *m/z* 100 to 700. Ionization parameters were as follows: capillary voltage, 4.5 kV and −3.5 kV; nebulizer gas flow, 1.5 L/min; CDL temperature, 250°C; heat block temperature, 250°C. Individual GTEs were diluted 1:50 in distilled water, and then 2.5 μM 4 hydroxybenzophenone was added as an internal standard. Samples were filtered through a 0.22-μm PTFE filter, and 5 μL was injected onto the column for each run.

Supplementary Figures and Tables

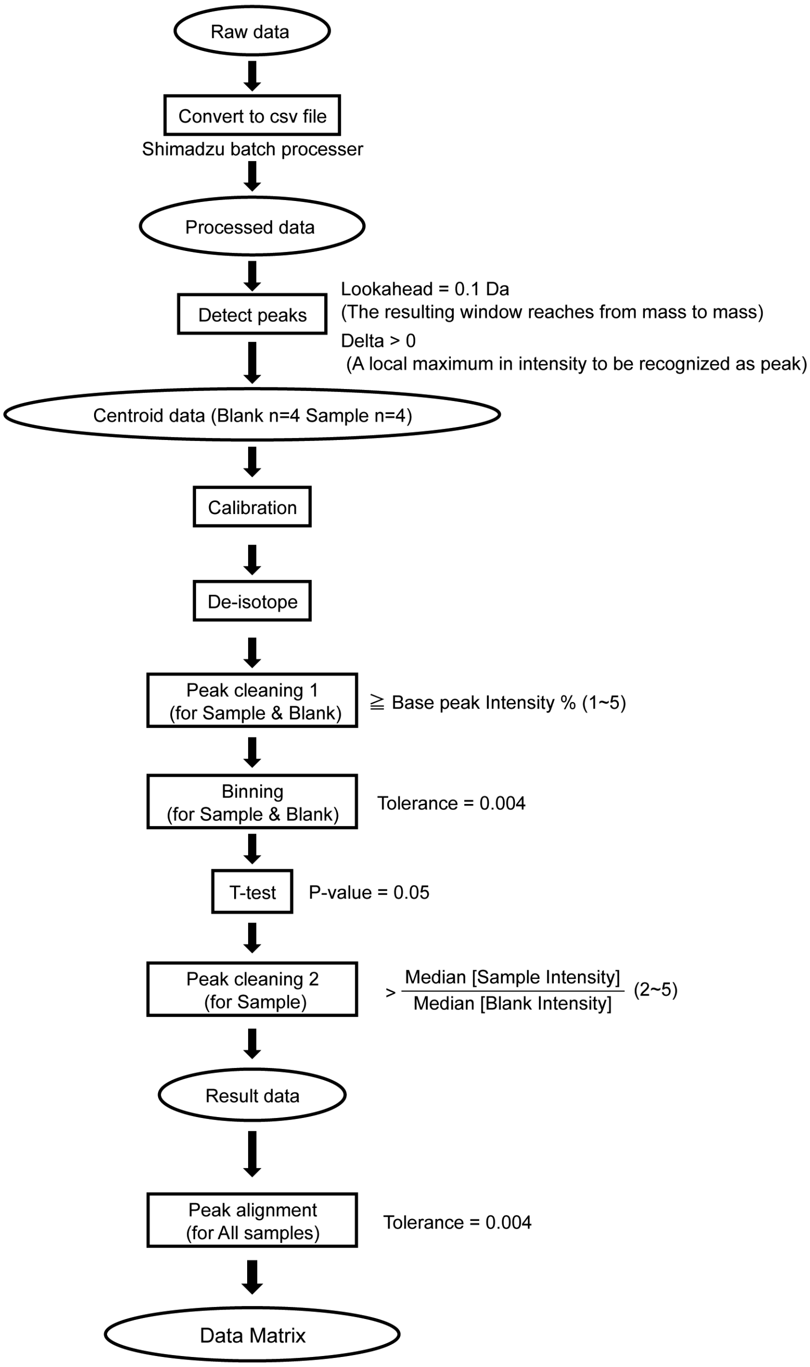

Supplementary Figure S1. The schematic flow of the peak picking and alignment of non-targeted MALDI–MS data.

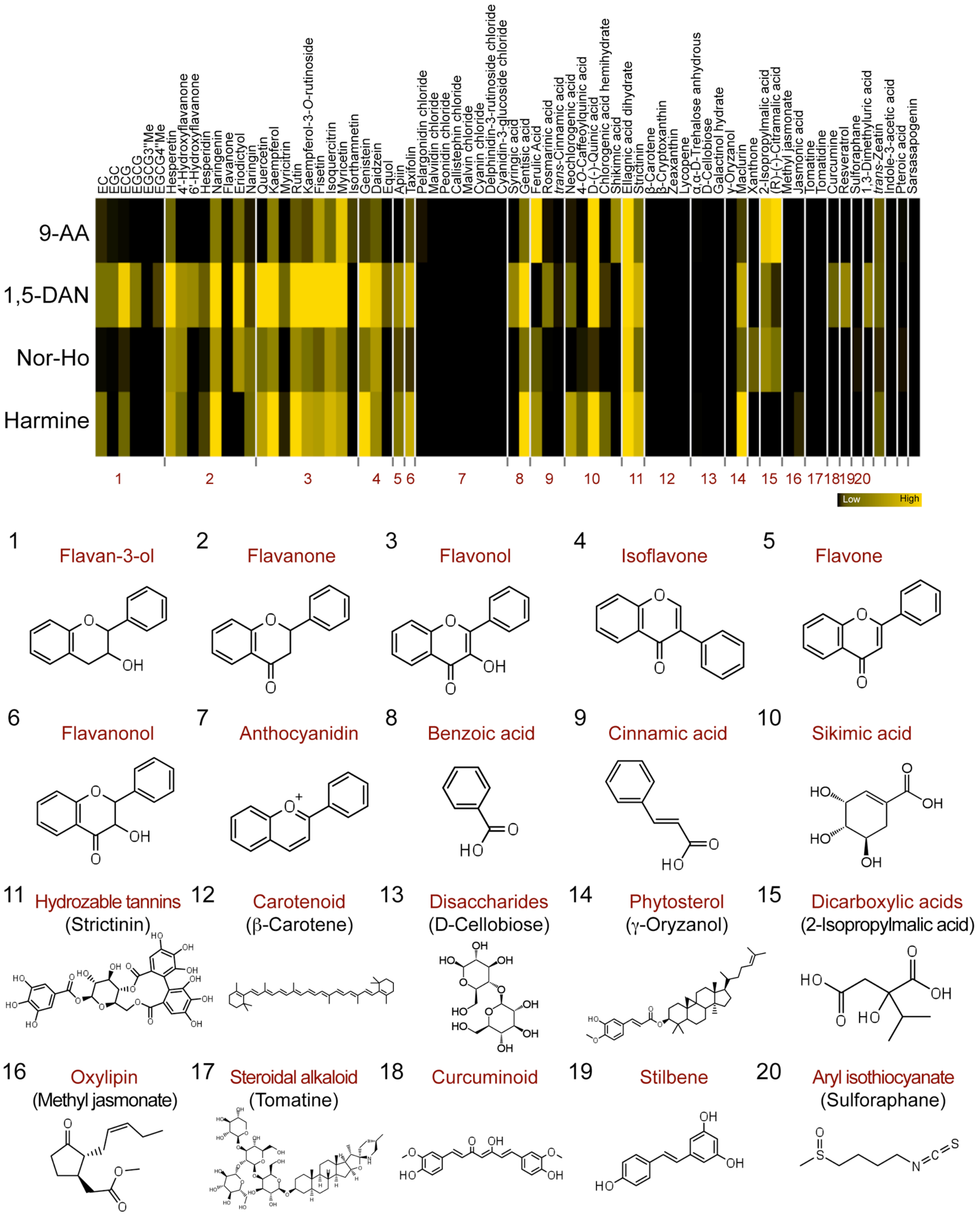

Supplementary Figure S2. Heatmap representation of the ionization of the representative phytochemicals by MALDI–MS using four matrices dissolved in MeOH solution. Heatmap analysis showing the different ionization rates of the 72 phytochemicals by the 4 matrices dissolved in 100% MeOH solution. In addition, 20 representative chemical structures of phytochemicals are illustrated.

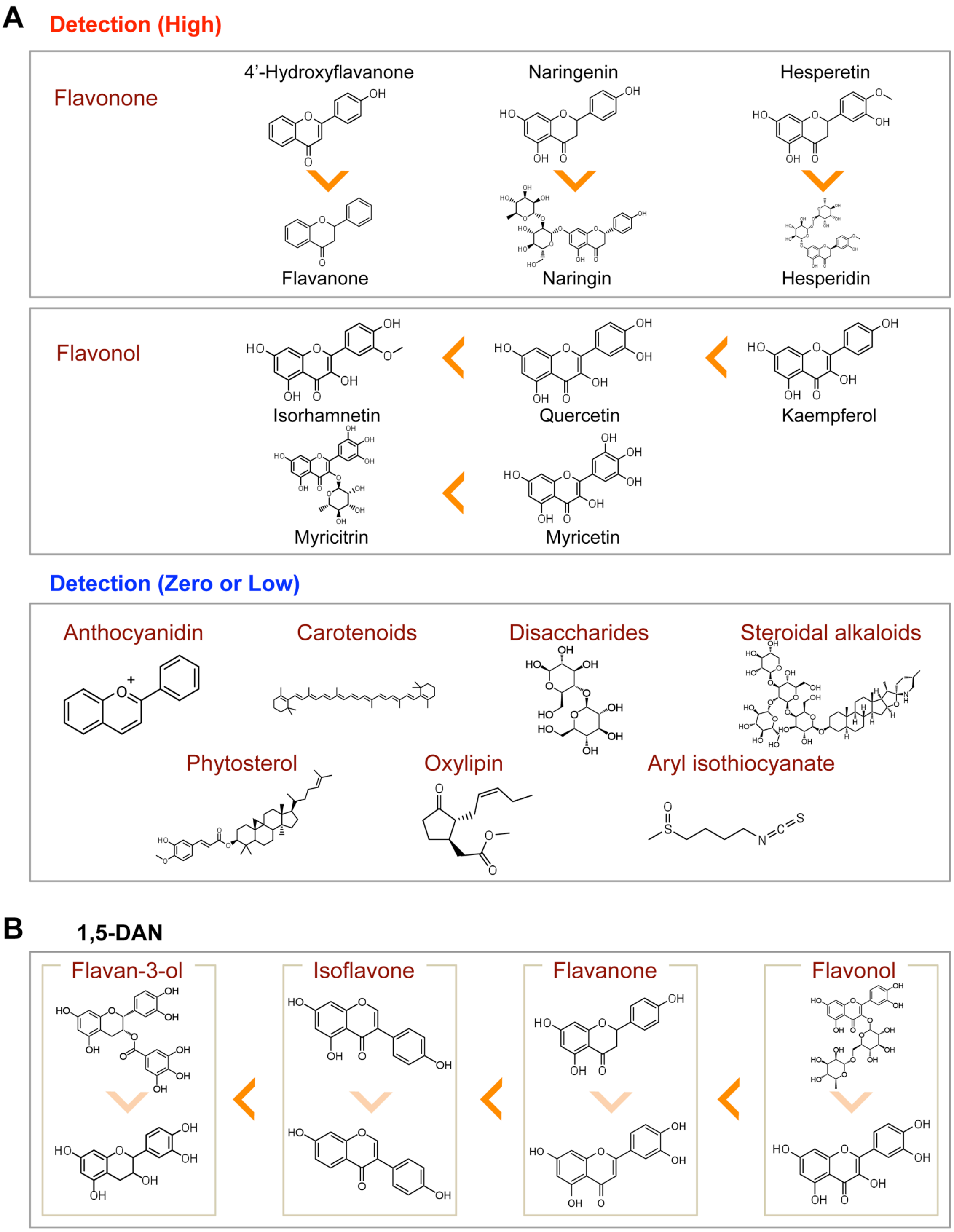

Supplementary Figure S3. The representative relationship between the chemical structure and the ionization efficiency of phytochemicals. Among the phytochemicals with different detectability, their representative relationships with the ionization efficiency are shown under conditions observed in (**A**) four matirces or (**B**) 1,5-DAN alone. The ionization efficacy of the phytochemicals is partially dependent on the position and the number of glycosylation, methylation, and hydoxylation.

A

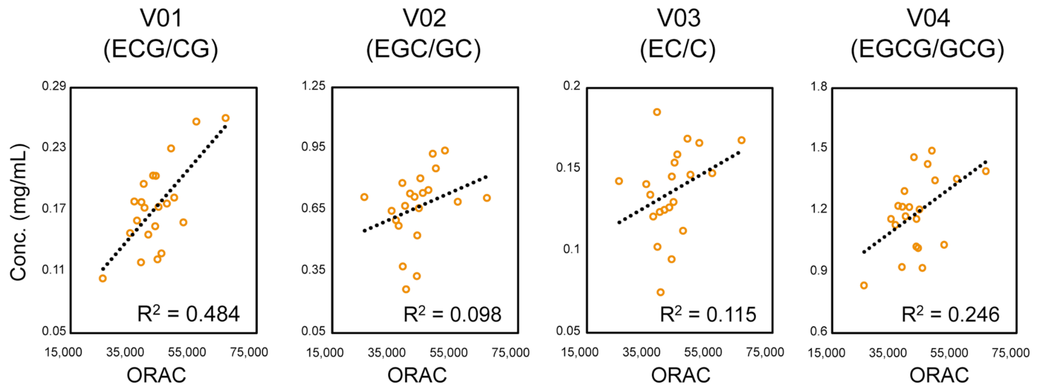

B

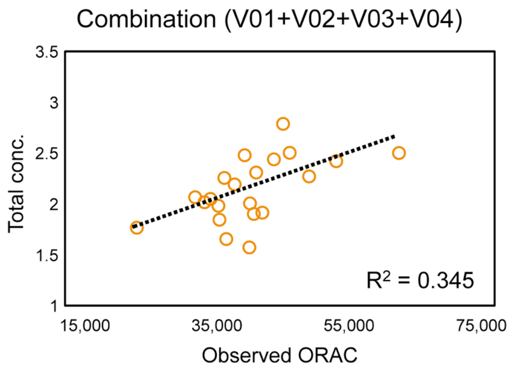

Supplementary Figure S4. The correlation plot of the highest VIP-valued compounds at the concentration level of GTEs. (**A**) Correlations between ORAC and the concentration of each of the top-4 components with the largest VIP values (>1). (**B**) Correlations between ORAC and the summed concentrations of the top-4-VIP components.

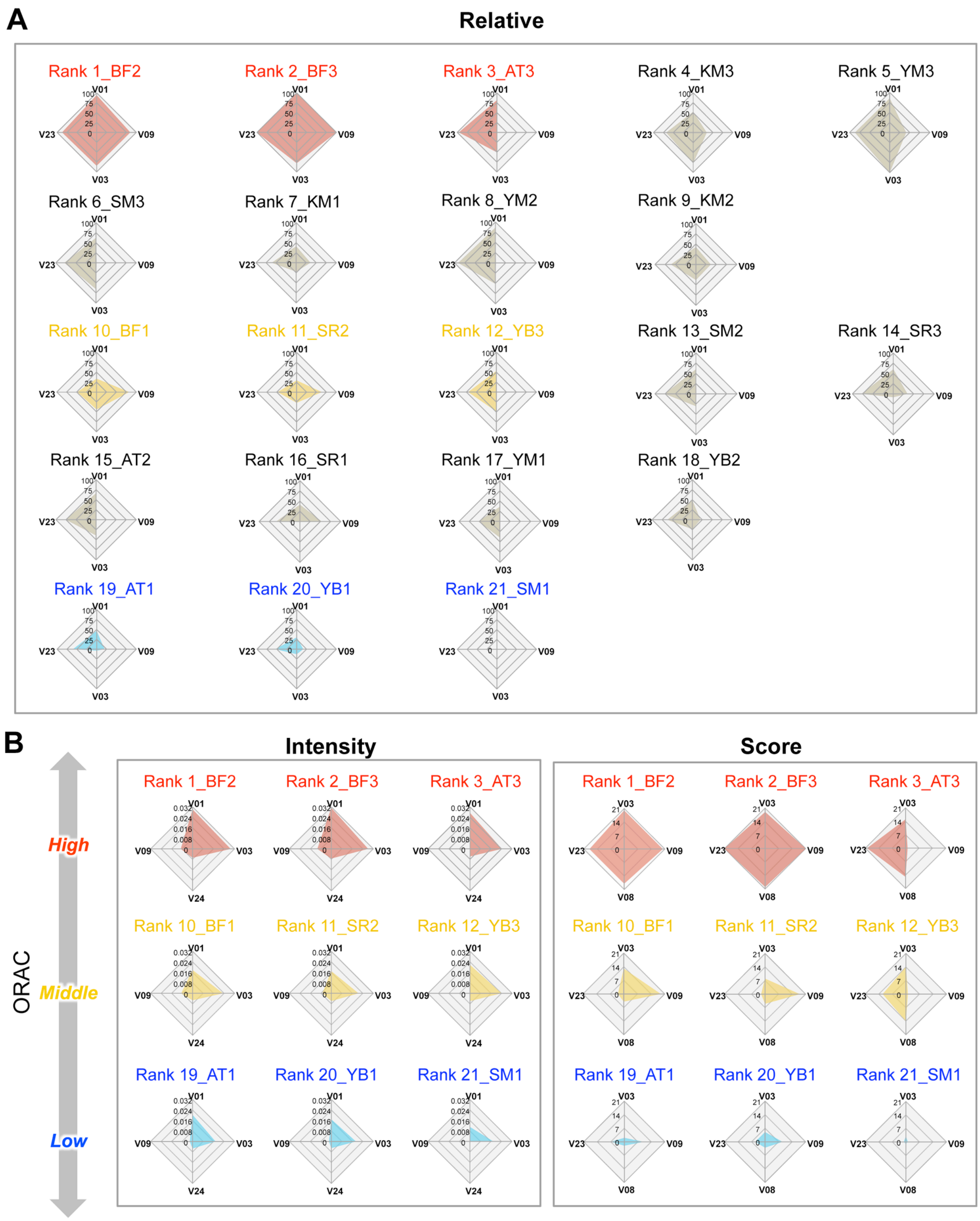

Supplementary Figure S5. The chart visualization of the observed ORAC values of GTEs using the selected combinations with the highest correlation values. (**A**) Observed ORAC values of GTEs visualized as radar charts using information from the 4 selected components (V01+V03+V09+V23). Charts for all GTEs are shown. (**B**) A representative section of the charts for nine GTEs is also illustrated using the Intensity data of the combination (V01+V03+V09+V24) or the Score data of the combination (V03+V08+V09+V23)

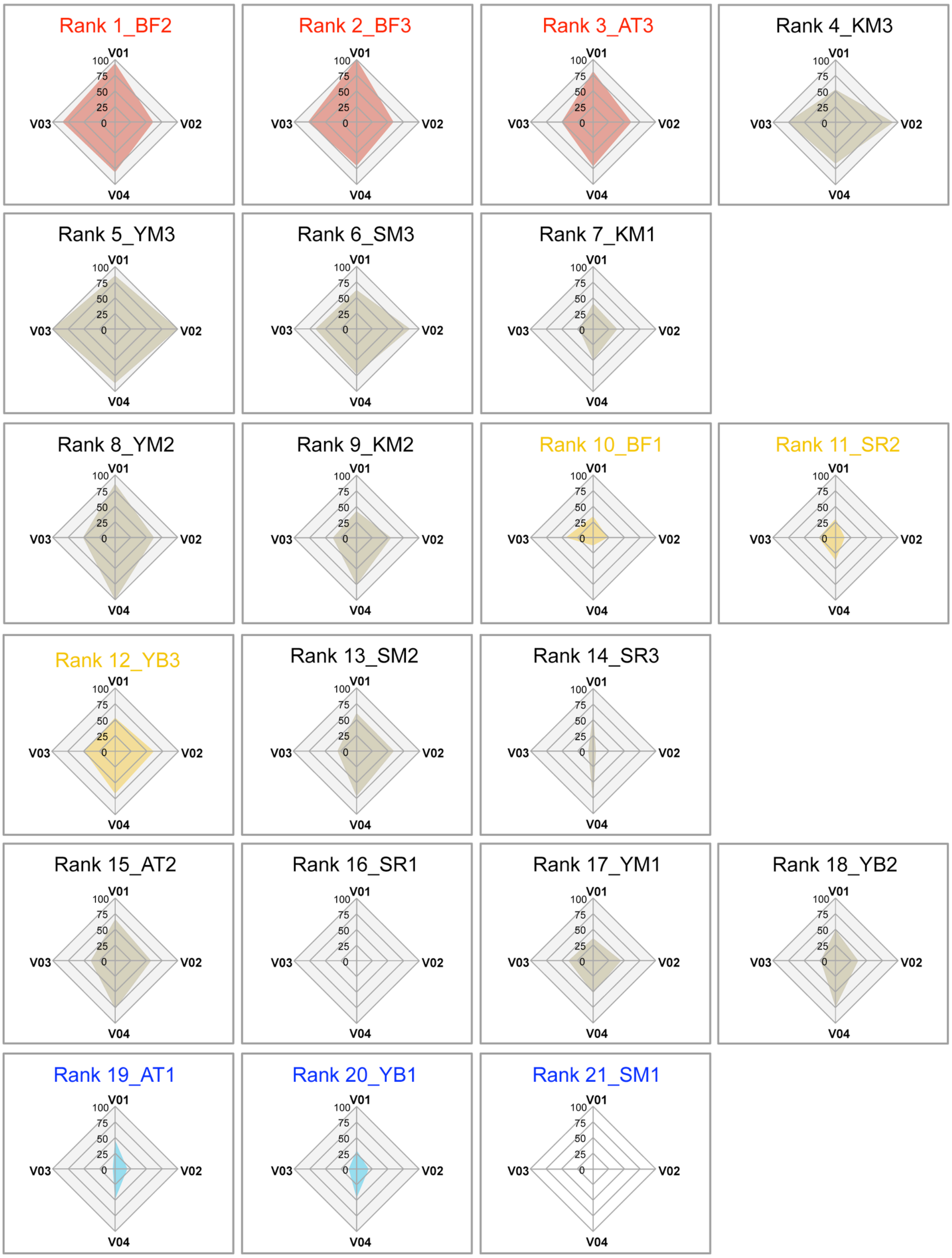

Supplementary Figure S6. The chart visualization of the observed ORAC values of GTEs using the selected combination with low correlation value. Observed ORAC values of GTEs visualized as radar charts using information from the 4 selected components (V01+V02+V03+V04) with low correlation value. Charts for all GTEs are shown.

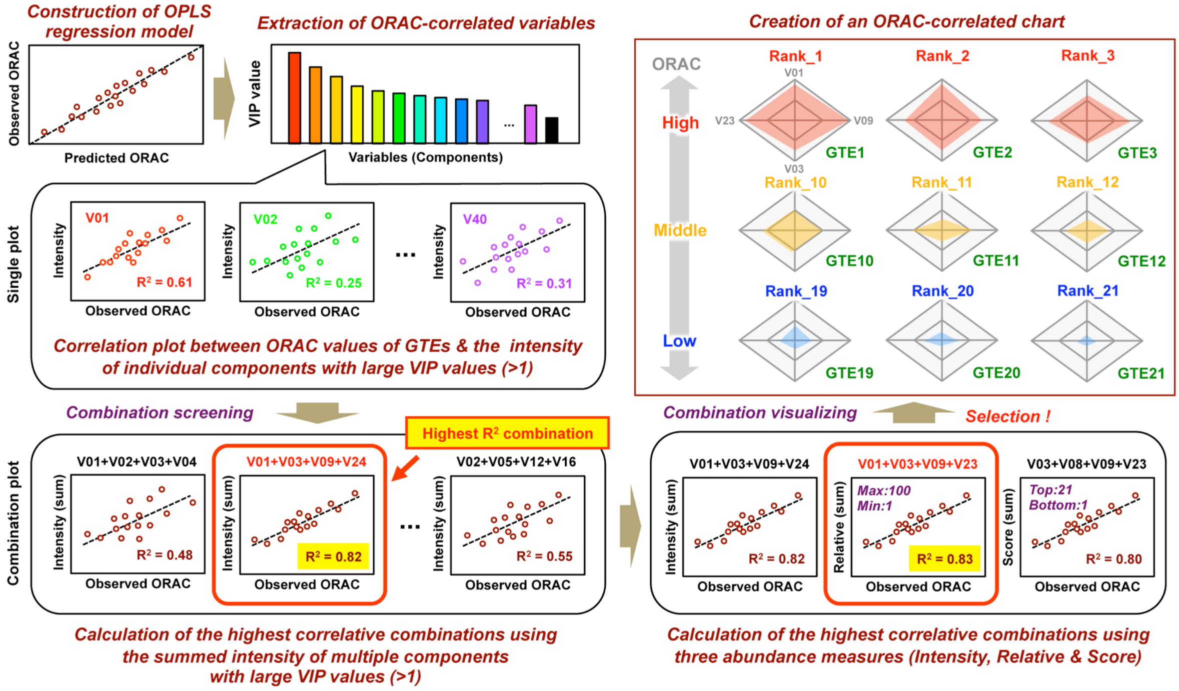

Supplementary Figure S7. Scheme of chemometrics-driven selection of GTE bioactivity-correlated chemical combinations.

In View of Pharmaceutical, Nutraceutical, & Food Functionality Researches		*In View of Metabolomics Research*		
	Conventional strategy	Novel strategy (MALDI-MS-MP)	Conventional strategy (LC-MS-MP or GC-MS-MP)	
Evaluation target	Single	Multiple		
Evaluation unit	Absolute quantity	Compositional balance		
Narrowing candidate components contributed to bioactivity	Multiple steps	One step		
Decision process of combination of multiple components	Hypothesis or experiment-driven	Hypothesis-generating or data-driven		
Bioassay validation (Frequency; labor-intensive)	High	Low		
		○	×	Direct analysis of crude sample
		◎	△	Rapid & high-throughput
		◎	△	Extensibility of sample number (>1,000)
		○	○	Theoretical presentation of combination for bioactivity
		△	◎	Detective performance of small molecules

Supplementary Figure S8. Comparison of the MALDI–MS-MP system with the conventional strategy in view of 2 distinct research fields.

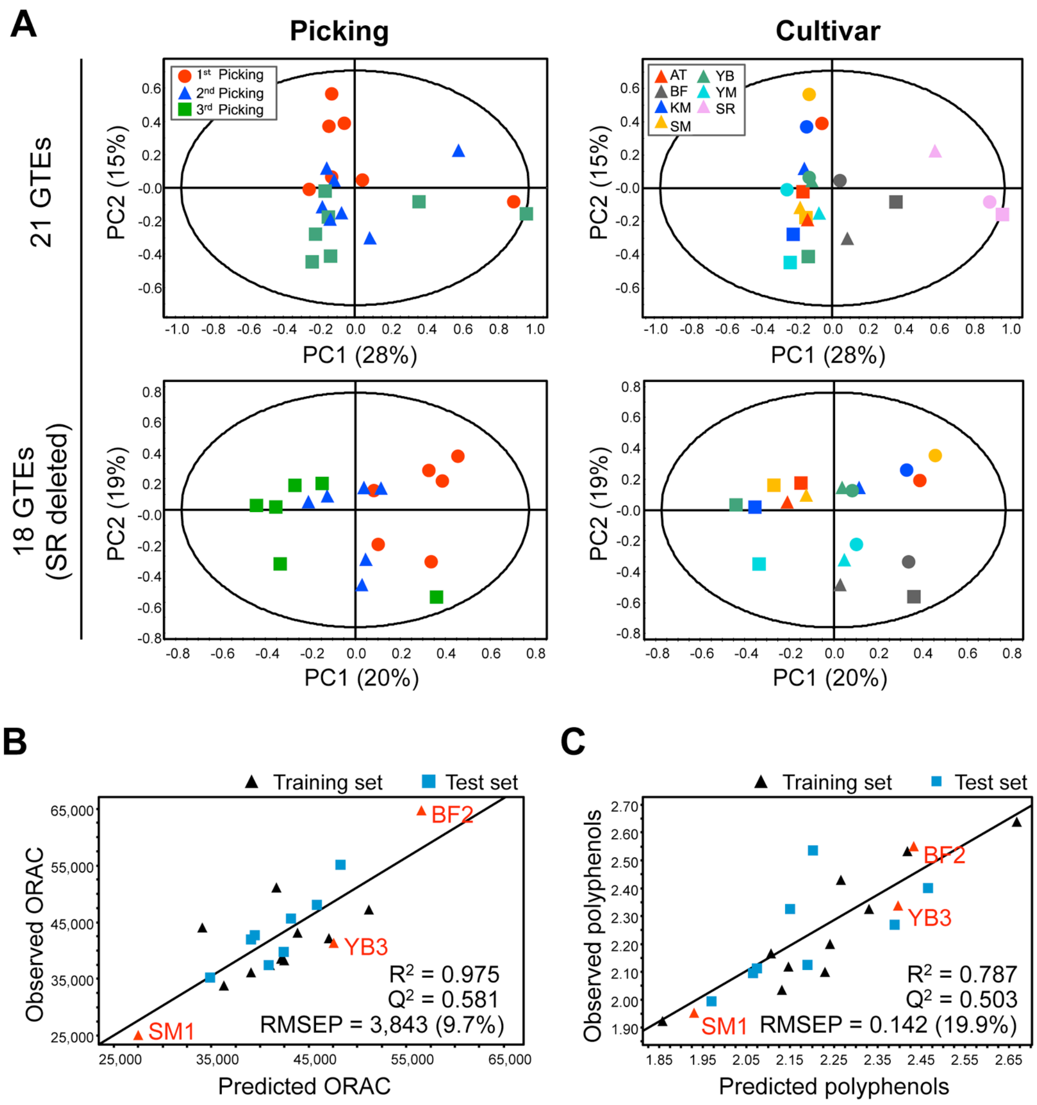

Supplementary Figure S9. LC–MS-MP of 21 GTEs for evaluating their quality and bioactivity. (**A**) PCA score plot of LC–MS datasets of 21 GTEs showing different clusters of MS profiles, based on the attributes of picking seasons and cultivars. (**B**) Models for predicting (**B**) ORAC or (**C**) total polyphenol content were calculated from the LC–MS datasets of 21 GTEs, including 13 training (black triangles) and 8 test (blue squares) sets.

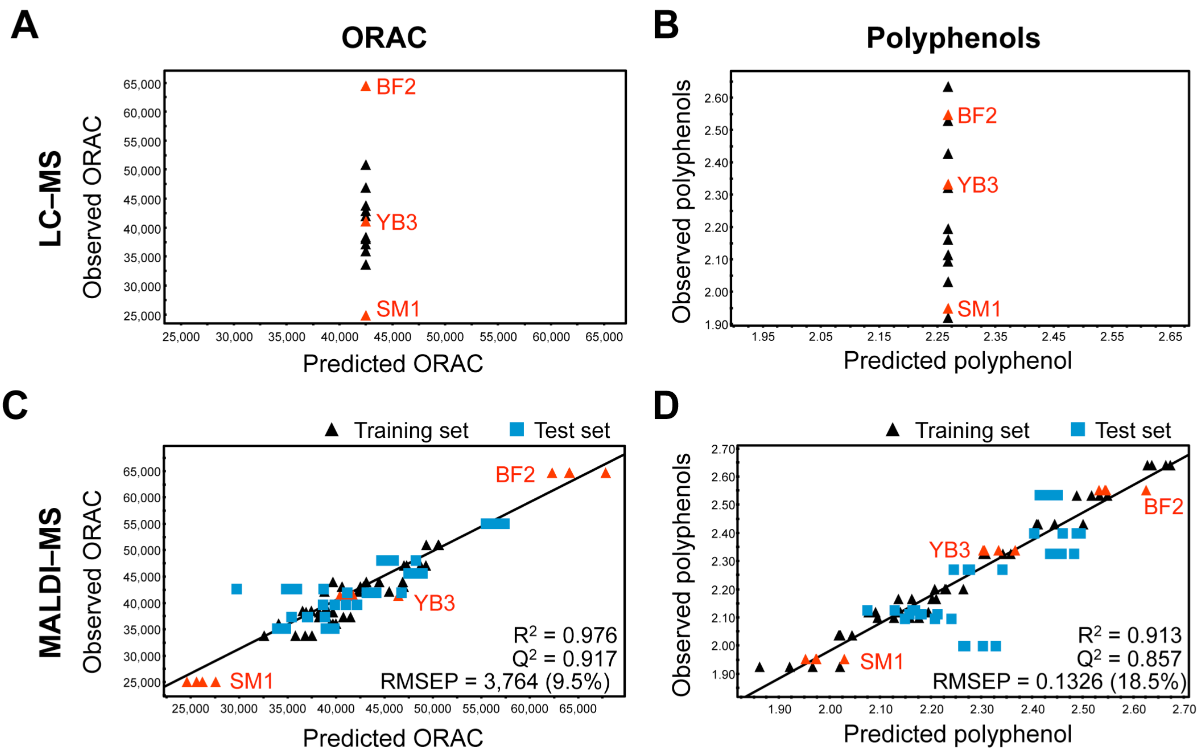

Supplementary Figure S10. The effect of components with the highest VIP values (>1) on the construction of OPLS regression models. Models for predicting ORAC (**A, C**) or total polyphenol content (**B, D**) were calculated from the LC–MS (**A, B**) or MALDI–MS (**C, D**) datasets of 21 GTEs, including 13 training (black triangles) and 8 test (blue squares) sets. These models were constructed using datasets excluding components with the highest VIP values (>1).

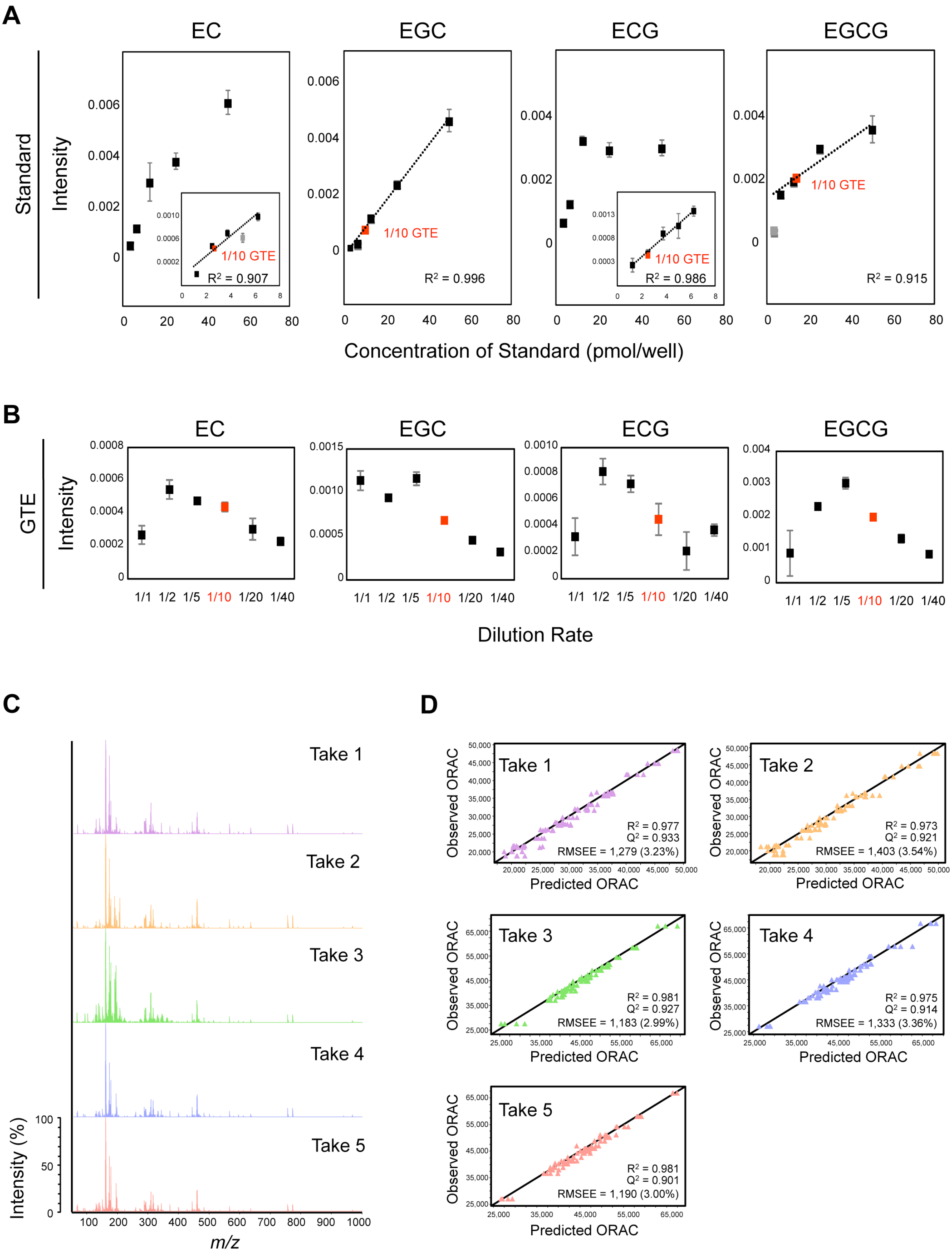

Supplementary Figure S11. The evaluation of the robustness of the present MALDI–MS-MP system. (**A**) The calibration curve from the representative GTE components (EGCG, EGC, ECG, and EC at the standard level) in MALDI–MS system. In EC and EGCG, the coefficient of determination (R^2) was calculated using datasets excluding the value of grey square symbol. Red square symbol indicates the data corresponding to 1/10 GTE sample. (**B**) Plots of these four compounds detected in a dilution series of the representative GTE (YB1). Data are shown as mean ± SD (*n*=3-5). (**C**) Verification of the repeatability of MS data acquired on a different day using the representative GTE (YB1). (**D**) OPLS regression models using such MS datasets.

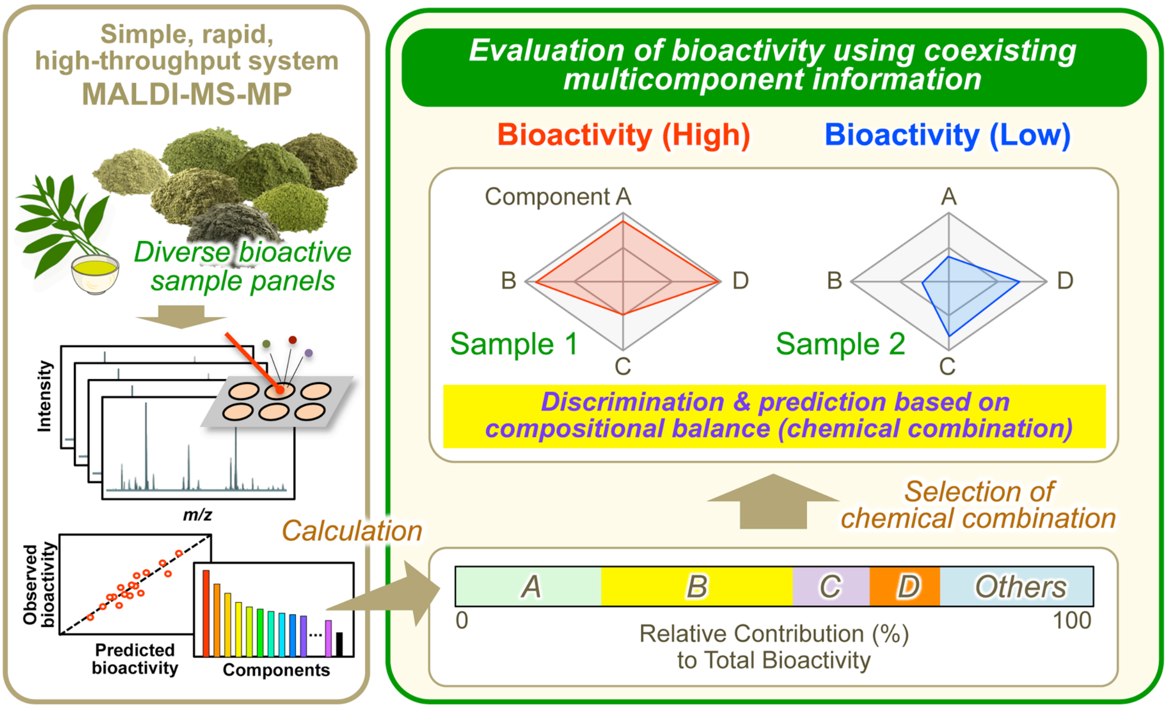

Supplementary Figure S12. Graphical representation of MALDI–MS-MP system for the bioactivity evaluation of diverse bioactive sample panels representing multicomponent systems and the effective selection of bioactivity-predictive chemical combinations.

Supplementary Table S1. The ionization rates of the representative phytochemicals for MALDI matrix screening.

	m/z		Acetone				MeOH			
	Theoretical	Observed [M-H]$^-$	9AA	1,5-DAN	Nor-Ho	Harmine	9AA	1,5-DAN	Nor-Ho	Harmine
EC	290.08	289.07	0.0016	0.0045	0.0006	0.0062	0.0011	0.0070	0.0005	0.0072
EGC	306.08	305.15	0.0003	0.0083	0.0001	0.0022	0.0005	0.0067	0.0003	0
ECG	442.09	441.17	0.0018	0.0123	0.0048	0.0058	0.0004	0.0282	0.0010	0.0046
EGCG	458.08	457.13	0.0002	0.0066	0.0009	0.0007	0	0.0092	0.0001	0
EGCG3"Me	472.10	471.12	0	0.0034	0.0002	0.0002	0	0	0.0001	0
EGCG4"Me	472.10	471.03	0	0.0033	0.0008	0.0004	0	0.0019	0.0001	0
Hesperetin	302.08	301.05	0.0095	0.0574	0.0154	0.0245	0.0049	0.0559	0.0147	0.0174
4'-Hydroxyflavanone	240.08	239.11	0.0026	0.0182	0.0086	0.0125	0	0.0144	0.0125	0.0060
6'-Hydroxyflavanone	240.08	239.07	0	0.0157	0.0001	0.0007	0	0.0127	0	0
Hesperidin	610.19	609.28	0.0008	0.0039	0.0005	0.0028	0	0.0036	0.0006	0.0018
Naringenin	272.07	271.09	0.0170	0.0524	0.0450	0.0444	0.0092	0.0683	0.0164	0.0582
Flavanone	224.08	223.10	0.0008	0.0010	0.0001	0	0	0	0.0001	0
Eriodictyol	288.07	287.12	0.0151	0.0500	0.0119	0.0658	0.0065	0.0520	0.0151	0
Naringin	580.18	579.23	0.0017	0.0059	0.0056	0.0033	0.0009	0.0020	0.0062	0.0049
Quercetin	302.04	301.08	0.0146	0.0364	0.0143	0.0245	0	0.0687	0.0054	0.0182
Kaempferol	286.05	285.09	0.0192	0.0559	0.0251	0.0399	0.0103	0.0538	0.0116	0.0344
Myricitrin	464.10	463.19	0.0012	0.0034	0.0004	0.0052	0	0.0045	0.0002	0.0009
Rutin	610.15	609.23	0.0112	0.0702	0.0189	0.0316	0.0030	0.0486	0.0126	0.0302
Kaempferol-3-O-rutinoside	594.16	593.23	0.0075	0.0410	0.0162	0.0183	0.0020	0.0328	0.0067	0.0185
Fisetin	286.05	285.10	0.0294	0.0339	0.0086	0.0183	0.0170	0.0372	0.0031	0.0168
Isoquercitrin	464.10	463.20	0.0165	0.0276	0.0179	0.0190	0.0048	0.0438	0.0151	0.0231
Myricetin	318.04	317.09	0.0275	0.0474	0.0179	0.0179	0.0264	0.0511	0.0088	0.0192
Isorthamnetin	316.06	315.08	0.0017	0	0	0	0.0018	0	0	0
Genistein	270.05	269.11	0.0037	0.0488	0.0145	0.0221	0.0019	0.0448	0.0071	0.0427
Daidzein	254.06	253.10	0.0035	0.0289	0.0148	0.0112	0.0034	0.0247	0.0159	0.0125
Equol	242.09	241.08	0	0.0080	0	0	0	0.0026	0	0.0001
Apiin	564.15	563.27	0.0023	0.0089	0.0076	0.0043	0	0.0120	0.0022	0.0085
Taxifolin	304.06	303.11	0.0074	0.0149	0.0085	0.0209	0.0031	0.0227	0.0015	0.0210
Pelargonidin chloride	306.03	305.04	0.0006	0	0	0	0.0007	0	0	0
Malviidin chloride	366.05	365.06	0	0	0	0.0002	0	0	0	0
Peonidin chloride	336.04	335.11	0.0004	0	0	0.0002	0	0	0	0
Callistephin chloride	468.08	-	0	0	0	0	0	0	0	0
Malvin chloride	690.16	-	0	0	0	0	0	0	0	0
Cyanin chloride	646.13	-	0	0	0	0	0	0	0	0
Delphinidin-3-rutinoside chloride	646.13	-	0	0	0	0	0	0	0	0
Cyanidin-3-glucoside chloride	484.08	483.04	0.0002	0	0	0	0	0	0	0
Syringic acid	198.05	197.12	0	0.0161	0.0018	0	0	0.0123	0	0
Gentisic acid	154.03	152.94	0.0135	0.0311	0.0065	0.0565	0.0072	0.0833	0.0071	0.0522
Ferulic Acid	194.06	193.06	0.2511	0	0.0036	0.0161	0.2590	0	0.0038	0.0138
Rosmarinic acid	360.08	359.16	0.0026	0	0.0015	0.0076	0.0007	0.0081	0.0003	0
trans-Cinnamic acid	148.05	146.99	0	0.0010	0	0	0	0.0007	0	0
Neochlorogenic acid	354.10	353.12	0.0030	0.0092	0.0017	0.0164	0.0009	0.0059	0	0.0177
4-O-Caffeoylquinic acid (Clorogenic acid)	354.10	353.13	0.0024	0.0011	0.0004	0.0123	0	0	0.0008	0.0046
D-(-)-Quinic acid	192.06	190.99	0.0857	0.0226	0.0072	0.0692	0.0581	0.0402	0.0014	0.0309
Chlorogenic acid hemihydrate	354.10	353.12	0.0022	0.0011	0.0009	0.0112	0	0.0014	0.0002	0.0047
Shikimic acid	174.06	173.04	0.0081	0	0.0034	0	0.0132	0	0	0
Ellagnic acid dihydrate	302.01	301.04	0.0620	0.0431	0.0404	0.0660	0.0648	0.0471	0.0285	0.0700
Strictinin	634.08	633.14	0.0073	0.0105	0.0083	0.0217	0.0057	0.0213	0.0043	0.0231
β-Carotene	536.44	535.38	0	0.0001	0	0	0	0	0	0
β-Cryptoxanthin	552.44	-	0	0	0	0	0	0	0	0
Zeaxanthin	568.43	-	0	0	0	0	0	0	0	0
Lycopene	536.44	-	0	0	0	0	0	0	0	0
α,α-D-Trehalose anhydrous	342.12	341.07	0	0	0.0001	0	0.0003	0	0	0.0003
D-Cellobiose	342.12	341.15	0.0002	0	0	0	0	0	0	0
Galactinol hydrate	342.12	341.09	0.0002	0	0	0	0	0	0	0
γ-Oryzanol	602.44	601.33	0	0.0010	0	0.0001	0.0002	0	0	0
Maclurin	262.05	261.07	0.0050	0.0266	0.0059	0.0456	0.0018	0.0199	0.0041	0.0307
Xanthone	196.06	195.07	0	0	0.0112	0	0	0	0.0036	0
2-Isopropylmalic acid	176.07	175.03	0.0353	0.0112	0.0129	0.0717	0.0269	0.0085	0.0126	0
(R)-(-)-Citramalic acid	148.04	146.95	0.0696	0.0012	0.0154	0	0.0815	0.0011	0.0069	0
Methyl jasmonate	224.14	223.10	0.0011	0	0	0	0	0	0	0
Jasmonic acid	210.13	209.09	0.0030	0.0093	0.0004	0.0070	0	0	0	0.0011
Tomatine	1033.55	1032.49	0	0.0004	0.0002	0	0	0	0.0001	0
Tomatidine	415.35	-	0	0	0	0	0	0	0	0
Curcumine	368.13	367.08	0.0003	0	0.0001	0	0.0002	0.0037	0.0001	0
Resveratrol	228.08	227.17	0	0.0189	0.0002	0.0076	0	0.0121	0	0
Sulforaphane	177.03	175.92	0	0	0.0010	0	0	0	0.0006	0
1,3-Dimethyluric acid	196.06	195.08	0	0.0397	0	0.0611	0	0.0100	0	0
trans-Zeatin	219.11	218.17	0.0046	0.0113	0	0.0139	0.0035	0.0041	0.0012	0.0041
Indole-3-acetic acid	175.06	173.97	0	0	0	0.0005	0	0	0	0
Pteroic acid	312.10	311.21	0	0	0.0014	0.0103	0.0004	0	0.0006	0
Sarsasapogenin	416.33	415.12	0	0	0	0	0	0	0	0

Supplementary Table S2. MALDI–MS datasets of 21 GTEs for multivariate statistical analyses.

Name	AT1	AT2	AT3	BF1	BF2	BF3	KM1	KM2	KM3	SM1	SM2	SM3	SR1	SR2	SR3	YB1	YB2	YB3	YM1	YM2	YM3
ORAC	35,647	38,661	51,482	42,552	65,085	55,612	44,400	43,107	48,544	25,445	40,191	46,160	37,930	42,436	38,953	34,246	36,545	41,729	37,746	43,496	47,566
Polyphenol	1.93	2.11	2.54	2.04	2.56	2.54	2.00	2.11	2.41	1.96	2.13	2.33	2.44	2.33	2.17	2.12	2.13	2.34	2.21	2.28	2.65
(m/z) 70.98	0.0022	0.0029	0.0033	0.0017	0.0001	0.0020	0.0018	0.0020	0.0026	0.0040	0.0018	0.0025	0.0001	0.0030	0.0029	0.0015	0.0022	0.0022	0.0022	0.0019	0.0020
112.97	0.0001	0.0001	0.0032	0.0001	0.0001	0.0001	0.0001	0.0001	0.0039	0.0001	0.0001	0.0001	0.0001	0.0059	0.0051	0.0001	0.0001	0.0001	0.0001	0.0001	0.0001
116.99	0.0066	0.0038	0.0001	0.0080	0.0031	0.0027	0.0051	0.0051	0.0026	0.0073	0.0077	0.0001	0.0071	0.0046	0.0039	0.0051	0.0056	0.0044	0.0060	0.0039	0.0001
122.99	0.0060	0.0076	0.0061	0.0056	0.0069	0.0068	0.0054	0.0065	0.0066	0.0054	0.0060	0.0089	0.0060	0.0055	0.0053	0.0055	0.0081	0.0090	0.0061	0.0071	0.0076
124.01	0.0080	0.0102	0.0089	0.0073	0.0096	0.0097	0.0071	0.0088	0.0092	0.0071	0.0085	0.0131	0.0081	0.0079	0.0083	0.0073	0.0105	0.0121	0.0080	0.0097	0.0104
125.02	0.0165	0.0201	0.0180	0.0151	0.0212	0.0205	0.0157	0.0189	0.0194	0.0156	0.0175	0.0265	0.0180	0.0186	0.0184	0.0155	0.0215	0.0241	0.0178	0.0194	0.0211
126.97	0.0085	0.0043	0.0001	0.0068	0.0051	0.0001	0.0066	0.0045	0.0001	0.0072	0.0036	0.0040	0.0065	0.0070	0.0038	0.0061	0.0069	0.0047	0.0073	0.0038	0.0040
128.00	0.0043	0.0018	0.0001	0.0167	0.0064	0.0024	0.0074	0.0061	0.0001	0.0152	0.0034	0.0001	0.0053	0.0061	0.0037	0.0093	0.0055	0.0021	0.0121	0.0030	0.0024
131.98	0.0055	0.0045	0.0036	0.0065	0.0061	0.0023	0.0069	0.0066	0.0046	0.0083	0.0048	0.0021	0.0059	0.0054	0.0039	0.0072	0.0094	0.0061	0.0047	0.0044	0.0066
132.97	0.0242	0.0127	0.0062	0.0282	0.0191	0.0129	0.0208	0.0180	0.0078	0.0217	0.0177	0.0053	0.0189	0.0249	0.0175	0.0174	0.0243	0.0112	0.0172	0.0144	0.0076
134.99	0.0044	0.0040	0.0001	0.0042	0.0001	0.0001	0.0001	0.0001	0.0001	0.0075	0.0036	0.0037	0.0089	0.0073	0.0049	0.0001	0.0058	0.0053	0.0001	0.0001	0.0001
136.99	0.0158	0.0199	0.0187	0.0158	0.0220	0.0205	0.0175	0.0210	0.0233	0.0191	0.0181	0.0276	0.0161	0.0200	0.0161	0.0156	0.0208	0.0248	0.0198	0.0194	0.0244
138.00	0.0092	0.0121	0.0113	0.0093	0.0129	0.0124	0.0103	0.0123	0.0135	0.0105	0.0110	0.0169	0.0084	0.0102	0.0087	0.0090	0.0119	0.0144	0.0111	0.0115	0.0144
138.77	0.0001	0.0001	0.0001	0.0001	0.0001	0.0001	0.0001	0.0001	0.0017	0.0001	0.0001	0.0021	0.0001	0.0001	0.0001	0.0001	0.0001	0.0001	0.0001	0.0001	0.0019
139.78	0.0023	0.0023	0.0020	0.0021	0.0023	0.0022	0.0021	0.0022	0.0022	0.0021	0.0001	0.0029	0.0018	0.0001	0.0001	0.0020	0.0025	0.0028	0.0021	0.0021	0.0026
144.99	0.0001	0.0001	0.0001	0.0182	0.0083	0.0001	0.0133	0.0082	0.0001	0.0217	0.0001	0.0001	0.0001	0.0001	0.0001	0.0197	0.0001	0.0001	0.0243	0.0001	0.0001
145.99	0.0107	0.0062	0.0051	0.0114	0.0074	0.0052	0.0119	0.0100	0.0071	0.0150	0.0081	0.0036	0.0096	0.0081	0.0063	0.0115	0.0106	0.0086	0.0129	0.0091	0.0076
149.00	0.0026	0.0030	0.0025	0.0035	0.0035	0.0030	0.0025	0.0032	0.0034	0.0034	0.0025	0.0025	0.0029	0.0035	0.0028	0.0025	0.0037	0.0040	0.0030	0.0024	0.0032
150.00	0.0062	0.0068	0.0063	0.0071	0.0092	0.0078	0.0070	0.0074	0.0074	0.0062	0.0061	0.0081	0.0063	0.0075	0.0057	0.0066	0.0075	0.0084	0.0079	0.0071	0.0087
151.00	0.0049	0.0053	0.0047	0.0049	0.0057	0.0054	0.0049	0.0054	0.0053	0.0050	0.0045	0.0066	0.0053	0.0073	0.0052	0.0047	0.0057	0.0068	0.0057	0.0051	0.0059
151.96	0.0037	0.0042	0.0040	0.0035	0.0046	0.0042	0.0036	0.0039	0.0045	0.0035	0.0044	0.0071	0.0059	0.0058	0.0058	0.0037	0.0046	0.0053	0.0037	0.0040	0.0050
152.95	0.0050	0.0061	0.0053	0.0044	0.0058	0.0058	0.0046	0.0058	0.0061	0.0041	0.0052	0.0078	0.0062	0.0066	0.0059	0.0045	0.0059	0.0067	0.0047	0.0056	0.0066
154.90	0.0063	0.0064	0.0048	0.0062	0.0059	0.0048	0.0050	0.0058	0.0059	0.0054	0.0048	0.0052	0.0067	0.0095	0.0073	0.0055	0.0065	0.0064	0.0059	0.0054	0.0060
157.87	0.0100	0.0096	0.0088	0.0079	0.0107	0.0099	0.0093	0.0100	0.0096	0.0001	0.0111	0.0091	0.0001	0.0103	0.0100	0.0097	0.0110	0.0099	0.0112	0.0109	0.0103
158.62	0.0001	0.0001	0.0001	0.0015	0.0001	0.0001	0.0001	0.0001	0.0001	0.0001	0.0001	0.0001	0.0001	0.0001	0.0001	0.0001	0.0019	0.0001	0.0001	0.0001	0.0001
159.67	0.0001	0.0001	0.0001	0.0001	0.0001	0.0068	0.0001	0.0001	0.0001	0.0001	0.0074	0.0001	0.0001	0.0001	0.0001	0.0001	0.0001	0.0001	0.0001	0.0001	0.0001
160.13	0.0096	0.0070	0.0042	0.0072	0.0063	0.0044	0.0077	0.0078	0.0050	0.0097	0.0069	0.0001	0.0103	0.0001	0.0053	0.0097	0.0090	0.0057	0.0099	0.0071	0.0056
160.83	0.0001	0.0001	0.0058	0.0001	0.0001	0.0064	0.0001	0.0001	0.0001	0.0001	0.0063	0.0061	0.0001	0.0001	0.0063	0.0001	0.0001	0.0001	0.0001	0.0001	0.0001
161.17	0.0083	0.0074	0.0055	0.0067	0.0060	0.0064	0.0067	0.0065	0.0058	0.0087	0.0001	0.0055	0.0001	0.0001	0.0054	0.0070	0.0071	0.0067	0.0082	0.0063	0.0064
161.97	0.0042	0.0040	0.0032	0.0035	0.0034	0.0036	0.0038	0.0038	0.0034	0.0044	0.0034	0.0035	0.0037	0.0032	0.0032	0.0039	0.0040	0.0035	0.0043	0.0039	0.0035
162.64	0.0001	0.0058	0.0053	0.0001	0.0059	0.0058	0.0001	0.0058	0.0056	0.0001	0.0058	0.0057	0.0001	0.0001	0.0058	0.0055	0.0001	0.0059	0.0063	0.0061	0.0060
163.05	0.0050	0.0001	0.0028	0.0054	0.0001	0.0039	0.0048	0.0044	0.0035	0.0069	0.0038	0.0042	0.0001	0.0001	0.0001	0.0055	0.0001	0.0052	0.0001	0.0001	0.0039
163.31	0.0001	0.0035	0.0001	0.0027	0.0031	0.0001	0.0001	0.0001	0.0001	0.0001	0.0001	0.0001	0.0034	0.0033	0.0032	0.0001	0.0039	0.0001	0.0037	0.0035	0.0001
163.76	0.0029	0.0028	0.0026	0.0028	0.0028	0.0030	0.0027	0.0028	0.0027	0.0025	0.0027	0.0027	0.0028	0.0024	0.0026	0.0030	0.0032	0.0029	0.0030	0.0029	0.0026
164.04	0.0023	0.0020	0.0001	0.0029	0.0031	0.0001	0.0021	0.0001	0.0032	0.0026	0.0018	0.0001	0.0027	0.0027	0.0001	0.0027	0.0029	0.0029	0.0001	0.0001	0.0039
164.45	0.0045	0.0046	0.0042	0.0036	0.0047	0.0046	0.0044	0.0046	0.0043	0.0001	0.0048	0.0044	0.0048	0.0048	0.0047	0.0045	0.0050	0.0047	0.0051	0.0049	0.0047
165.05	0.0136	0.0151	0.0122	0.0150	0.0164	0.0149	0.0131	0.0164	0.0148	0.0158	0.0139	0.0151	0.0158	0.0184	0.0159	0.0128	0.0172	0.0171	0.0151	0.0134	0.0155
165.60	0.0025	0.0024	0.0025	0.0022	0.0024	0.0028	0.0023	0.0024	0.0026	0.0021	0.0023	0.0025	0.0027	0.0024	0.0026	0.0025	0.0027	0.0026	0.0026	0.0026	0.0025
166.02	0.0201	0.0266	0.0267	0.0216	0.0276	0.0264	0.0235	0.0272	0.0331	0.0220	0.0288	0.0288	0.0189	0.0217	0.0198	0.0208	0.0238	0.0272	0.0247	0.0270	0.0332
166.26	0.0001	0.0043	0.0039	0.0001	0.0001	0.0001	0.0001	0.0001	0.0001	0.0001	0.0041	0.0001	0.0001	0.0001	0.0001	0.0041	0.0001	0.0001	0.0001	0.0046	0.0001
167.02	0.0335	0.0428	0.0393	0.0317	0.0412	0.0405	0.0332	0.0413	0.0419	0.0293	0.0416	0.0425	0.0355	0.0349	0.0389	0.0326	0.0401	0.0414	0.0363	0.0424	0.0440
167.46	0.0019	0.0020	0.0020	0.0016	0.0001	0.0021	0.0019	0.0020	0.0020	0.0001	0.0021	0.0001	0.0020	0.0019	0.0018	0.0020	0.0020	0.0019	0.0023	0.0021	0.0018
168.06	0.0359	0.0455	0.0422	0.0336	0.0443	0.0441	0.0349	0.0443	0.0419	0.0311	0.0441	0.0475	0.0424	0.0446	0.0519	0.0349	0.0441	0.0458	0.0382	0.0443	0.0441
169.13	0.1112	0.1121	0.0953	0.0867	0.1096	0.1026	0.1011	0.1090	0.0956	0.1049	0.1002	0.1151	0.1178	0.1090	0.1160	0.0999	0.1190	0.1107	0.1169	0.1115	0.1049
170.08	0.0436	0.0001	0.0001	0.0001	0.0001	0.0001	0.0382	0.0001	0.0001	0.0001	0.0001	0.0001	0.0001	0.0001	0.0001	0.0001	0.0001	0.0001	0.0001	0.0001	0.0001
174.83	0.0038	0.0046	0.0040	0.0024	0.0041	0.0042	0.0032	0.0043	0.0041	0.0027	0.0041	0.0042	0.0041	0.0034	0.0044	0.0038	0.0048	0.0047	0.0040	0.0046	0.0044
175.44	0.0020	0.0019	0.0017	0.0128	0.0001	0.0019	0.0018	0.0019	0.0017	0.0181	0.0001	0.0022	0.0001	0.0001	0.0001	0.0020	0.0020	0.0021	0.0023	0.0021	0.0001
176.01	0.0048	0.0045	0.0001	0.0047	0.0001	0.0032	0.0044	0.0046	0.0001	0.0001	0.0001	0.0001	0.0045	0.0001	0.0040	0.0044	0.0050	0.0001	0.0049	0.0041	0.0001
176.71	0.0027	0.0029	0.0026	0.0016	0.0026	0.0027	0.0022	0.0027	0.0026	0.0018	0.0024	0.0028	0.0029	0.0021	0.0026	0.0024	0.0031	0.0031	0.0027	0.0028	0.0028
177.14	0.0072	0.0001	0.0001	0.0070	0.0001	0.0046	0.0066	0.0001	0.0001	0.0091	0.0001	0.0001	0.0074	0.0073	0.0064	0.0063	0.0001	0.0001	0.0074	0.0001	0.0001
177.89	0.0028	0.0028	0.0021	0.0027	0.0022	0.0023	0.0026	0.0029	0.0023	0.0030	0.0022	0.0001	0.0030	0.0024	0.0027	0.0026	0.0032	0.0026	0.0027	0.0026	0.0024
178.61	0.0022	0.0023	0.0022	0.0016	0.0020	0.0023	0.0020	0.0021	0.0021	0.0001	0.0019	0.0024	0.0025	0.0020	0.0023	0.0020	0.0024	0.0025	0.0022	0.0023	0.0023
179.11	0.0113	0.0099	0.0085	0.0111	0.0101	0.0097	0.0092	0.0103	0.0095	0.0119	0.0098	0.0082	0.0143	0.0148	0.0127	0.0099	0.0120	0.0102	0.0103	0.0081	0.0089
179.80	0.0020	0.0022	0.0017	0.0017	0.0001	0.0019	0.0019	0.0021	0.0019	0.0001	0.0001	0.0001	0.0025	0.0001	0.0019	0.0018	0.0024	0.0021	0.0018	0.0020	0.0019
182.12	0.0001	0.0001	0.0001	0.0027	0.0001	0.0019	0.0001	0.0001	0.0001	0.0001	0.0001	0.0001	0.0001	0.0001	0.0001	0.0001	0.0001	0.0001	0.0001	0.0001	0.0001
183.10	0.0021	0.0001	0.0001	0.0087	0.0090	0.0106	0.0037	0.0038	0.0036	0.0001	0.0001	0.0001	0.0056	0.0067	0.0037	0.0018	0.0021	0.0001	0.0001	0.0001	0.0044
184.11	0.0024	0.0001	0.0001	0.0001	0.0001	0.0001	0.0022	0.0001	0.0001	0.0001	0.0001	0.0001	0.0022	0.0001	0.0001	0.0019	0.0001	0.0001	0.0001	0.0001	0.0001
191.14	0.0486	0.0271	0.0222	0.0849	0.0977	0.0701	0.0562	0.0491	0.0493	0.0710	0.0378	0.0262	0.0912	0.1112	0.0801	0.0661	0.0673	0.0433	0.0666	0.0406	0.0551
202.14	0.0074	0.0001	0.0001	0.0001	0.0001	0.0001	0.0001	0.0001	0.0001	0.0001	0.0001	0.0001	0.0001	0.0001	0.0001	0.0064	0.0001	0.0001	0.0001	0.0001	0.0001
204.14	0.0159	0.0001	0.0001	0.0001	0.0001	0.0001	0.0001	0.0001	0.0001	0.0001	0.0001	0.0001	0.0001	0.0001	0.0001	0.0121	0.0001	0.0001	0.0001	0.0001	0.0001
207.19	0.0038	0.0001	0.0001	0.0001	0.0001	0.0001	0.0001	0.0001	0.0001	0.0054	0.0001	0.0001	0.0001	0.0001	0.0001	0.0001	0.0001	0.0001	0.0001	0.0001	0.0001
209.19	0.0085	0.0001	0.0001	0.0096	0.0001	0.0001	0.0076	0.0001	0.0001	0.0087	0.0001	0.0001	0.0085	0.0001	0.0001	0.0075	0.0087	0.0001	0.0078	0.0001	0.0001
218.15	0.0032	0.0001	0.0001	0.0001	0.0001	0.0001	0.0001	0.0001	0.0001	0.0001	0.0001	0.0001	0.0001	0.0001	0.0001	0.0027	0.0001	0.0001	0.0001	0.0001	0.0001
220.15	0.0034	0.0001	0.0001	0.0001	0.0001	0.0001	0.0025	0.0001	0.0001	0.0001	0.0001	0.0001	0.0001	0.0001	0.0001	0.0026	0.0001	0.0001	0.0001	0.0001	0.0001
261.16	0.0001	0.0001	0.0001	0.0001	0.0001	0.0001	0.0001	0.0001	0.0001	0.0001	0.0001	0.0001	0.0026	0.0023	0.0001	0.0001	0.0001	0.0001	0.0001	0.0001	0.0001
270.20	0.0001	0.0001	0.0027	0.0029	0.0001	0.0001	0.0034	0.0001	0.0001	0.0040	0.0001	0.0029	0.0001	0.0001	0.0001	0.0001	0.0001	0.0001	0.0001	0.0001	0.0030
271.20	0.0053	0.0056	0.0058	0.0053	0.0068	0.0074	0.0052	0.0052	0.0056	0.0001	0.0052	0.0059	0.0059	0.0058	0.0059	0.0051	0.0053	0.0059	0.0054	0.0061	0.0063
272.15	0.0020	0.0022	0.0023	0.0021	0.0028	0.0030	0.0020	0.0001	0.0021	0.0001	0.0020	0.0024	0.0022	0.0021	0.0023	0.0020	0.0020	0.0022	0.0021	0.0024	0.0025
273.14	0.0028	0.0030	0.0037	0.0035	0.0047	0.0047	0.0039	0.0039	0.0037	0.0030	0.0033	0.0035	0.0059	0.0058	0.0054	0.0038	0.0033	0.0033	0.0039	0.0036	0.0039
274.12	0.0001	0.0001	0.0018	0.0018	0.0021	0.0020	0.0019	0.0001	0.0018	0.0001	0.0001	0.0001	0.0025	0.0022	0.0001	0.0018	0.0001	0.0001	0.0001	0.0001	0.0018
275.32	0.0037	0.0038	0.0031	0.0032	0.0038	0.0033	0.0033	0.0033	0.0032	0.0026	0.0032	0.0036	0.0033	0.0026	0.0026	0.0037	0.0039	0.0037	0.0039	0.0040	0.0038
284.08	0.0001	0.0047	0.0060	0.0001	0.0001	0.0048	0.0058	0.0048	0.0059	0.0001	0.0061	0.0001	0.0066	0.0063	0.0066	0.0053	0.0001	0.0051	0.0080	0.0093	0.0073
285.12	0.0125	0.0145	0.0168	0.0077	0.0110	0.0114	0.0172	0.0152	0.0149	0.0133	0.0179	0.0169	0.0197	0.0156	0.0161	0.0167	0.0135	0.0140	0.0149	0.0170	0.0131
286.15	0.0071	0.0092	0.0101	0.0060	0.0082	0.0093	0.0085	0.0091	0.0098	0.0064	0.0097	0.0106	0.0094	0.0084	0.0092	0.0080	0.0080	0.0091	0.0077	0.0098	0.0092
287.18	0.0184	0.0246	0.0243	0.0187	0.0253	0.0271	0.0203	0.0237	0.0253	0.0150	0.0235	0.0269	0.0211	0.0202	0.0229	0.0193	0.0224	0.0251	0.0207	0.0261	0.0268
288.20	0.0094	0.0132	0.0138	0.0097	0.0140	0.0156	0.0101	0.0122	0.0140	0.0073	0.0125	0.0164	0.0101	0.0100	0.0121	0.0096	0.0113	0.0133	0.0104	0.0139	0.0146
289.19	0.0174	0.0226	0.0242	0.0232	0.0291	0.0281	0.0201	0.0224	0.0280	0.0177	0.0213	0.0264	0.0171	0.0207	0.0181	0.0188	0.0203	0.0239	0.0226	0.0243	0.0313
291.19	0.0029	0.0033	0.0030	0.0034	0.0034	0.0032	0.0030	0.0033	0.0032	0.0030	0.0030	0.0033	0.0033	0.0029	0.0028	0.0030	0.0033	0.0033	0.0032	0.0033	0.0036
292.18	0.0017	0.0019	0.0017	0.0021	0.0001	0.0019	0.0018	0.0019	0.0018	0.0001	0.0001	0.0001	0.0001	0.0001	0.0001	0.0017	0.0019	0.0001	0.0001	0.0001	0.0019
293.20	0.0001	0.0001	0.0026	0.0001	0.0001	0.0029	0.0001	0.0001	0.0001	0.0001	0.0001	0.0001	0.0001	0.0001	0.0001	0.0001	0.0001	0.0001	0.0001	0.0032	0.0034
300.18	0.0064	0.0070	0.0118	0.0001	0.0001	0.0071	0.0072	0.0056	0.0103	0.0070	0.0069	0.0087	0.0105	0.0123	0.0133	0.0055	0.0001	0.0081	0.0068	0.0079	0.0071
301.18	0.0122	0.0149	0.0254	0.0057	0.0082	0.0118	0.0138	0.0107	0.0193	0.0130	0.0142	0.0189	0.0195	0.0219	0.0234	0.0110	0.0090	0.0152	0.0091	0.0102	0.0092
303.19	0.0061	0.0073	0.0081	0.0053	0.0062	0.0068	0.0070	0.0068	0.0078	0.0064	0.0068	0.0070	0.0069	0.0065	0.0066	0.0059	0.0059	0.0066	0.0063	0.0067	0.0071
304.16	0.0072	0.0097	0.0097	0.0068	0.0089	0.0098	0.0081	0.0091	0.0101	0.0069	0.0088	0.0101	0.0066	0.0064	0.0068	0.0073	0.0079	0.0086	0.0081	0.0097	0.0107
305.19	0.0407	0.0558	0.0571	0.0422	0.0571	0.0564	0.0484	0.0549	0.0695	0.0465	0.0564	0.0668	0.0336	0.0390	0.0348	0.0407	0.0474	0.0571	0.0508	0.0576	0.0725

(continued)

Name	AT1	AT2	AT3	BF1	BF2	BF3	KM1	KM2	KM3	SM1	SM2	SM3	SR1	SR2	SR3	YB1	YB2	YB3	YM1	YM2	YM3
ORAC	35,647	38,661	51,482	42,552	65,085	55,612	44,400	43,107	48,544	25,445	40,191	46,160	37,930	42,436	38,953	34,246	36,545	41,729	37,746	43,496	47,566
Polyphenol	1.93	2.11	2.54	2.04	2.56	2.54	2.00	2.11	2.41	1.96	2.13	2.33	2.44	2.33	2.17	2.12	2.13	2.34	2.21	2.28	2.65
306.80	0.0001	0.0019	0.0019	0.0015	0.0001	0.0018	0.0001	0.0001	0.0019	0.0001	0.0001	0.0001	0.0001	0.0001	0.0001	0.0001	0.0001	0.0001	0.0001	0.0001	0.0001
307.24	0.0044	0.0053	0.0050	0.0040	0.0001	0.0001	0.0049	0.0051	0.0001	0.0043	0.0001	0.0001	0.0039	0.0040	0.0037	0.0044	0.0049	0.0052	0.0051	0.0051	0.0001
307.82	0.0021	0.0028	0.0031	0.0020	0.0001	0.0027	0.0024	0.0025	0.0033	0.0022	0.0021	0.0030	0.0020	0.0021	0.0001	0.0021	0.0022	0.0027	0.0023	0.0027	0.0028
308.24	0.0027	0.0030	0.0028	0.0025	0.0024	0.0027	0.0029	0.0030	0.0029	0.0026	0.0024	0.0021	0.0025	0.0021	0.0001	0.0027	0.0028	0.0028	0.0030	0.0031	0.0029
308.82	0.0001	0.0018	0.0022	0.0001	0.0001	0.0001	0.0017	0.0001	0.0023	0.0001	0.0001	0.0020	0.0001	0.0001	0.0001	0.0001	0.0001	0.0001	0.0001	0.0001	0.0001
309.20	0.0057	0.0068	0.0061	0.0053	0.0059	0.0061	0.0058	0.0066	0.0067	0.0001	0.0062	0.0061	0.0001	0.0051	0.0050	0.0058	0.0064	0.0066	0.0062	0.0072	0.0070
317.52	0.0020	0.0001	0.0084	0.0001	0.0001	0.0001	0.0001	0.0001	0.0001	0.0001	0.0001	0.0089	0.0124	0.0123	0.0135	0.0017	0.0001	0.0001	0.0001	0.0001	0.0001
319.16	0.0052	0.0060	0.0054	0.0047	0.0053	0.0056	0.0050	0.0054	0.0058	0.0042	0.0052	0.0061	0.0057	0.0049	0.0001	0.0051	0.0058	0.0060	0.0052	0.0059	0.0061
337.23	0.0001	0.0001	0.0001	0.0036	0.0043	0.0001	0.0001	0.0001	0.0001	0.0001	0.0031	0.0001	0.0001	0.0001	0.0001	0.0045	0.0046	0.0032	0.0001	0.0001	0.0001
338.28	0.0025	0.0021	0.0019	0.0026	0.0031	0.0023	0.0024	0.0021	0.0020	0.0001	0.0023	0.0001	0.0022	0.0001	0.0001	0.0030	0.0029	0.0024	0.0024	0.0024	0.0023
339.30	0.0001	0.0001	0.0001	0.0001	0.0001	0.0001	0.0054	0.0001	0.0001	0.0001	0.0001	0.0001	0.0001	0.0001	0.0001	0.0053	0.0001	0.0001	0.0001	0.0001	0.0001
354.53	0.0019	0.0001	0.0001	0.0014	0.0001	0.0001	0.0017	0.0001	0.0001	0.0001	0.0001	0.0001	0.0001	0.0001	0.0001	0.0018	0.0001	0.0001	0.0020	0.0001	0.0001
413.46	0.0001	0.0001	0.0001	0.0017	0.0021	0.0017	0.0021	0.0001	0.0001	0.0001	0.0001	0.0001	0.0024	0.0021	0.0001	0.0024	0.0001	0.0001	0.0024	0.0001	0.0001
423.19	0.0001	0.0001	0.0001	0.0001	0.0001	0.0001	0.0001	0.0001	0.0001	0.0001	0.0001	0.0001	0.0033	0.0027	0.0031	0.0001	0.0001	0.0001	0.0001	0.0001	0.0001
429.43	0.0001	0.0001	0.0001	0.0001	0.0001	0.0001	0.0001	0.0001	0.0001	0.0001	0.0001	0.0001	0.0029	0.0026	0.0024	0.0001	0.0001	0.0001	0.0001	0.0001	0.0001
431.61	0.0001	0.0001	0.0001	0.0001	0.0001	0.0024	0.0001	0.0031	0.0026	0.0001	0.0032	0.0031	0.0001	0.0001	0.0001	0.0001	0.0001	0.0001	0.0001	0.0001	0.0001
439.11	0.0001	0.0019	0.0022	0.0001	0.0001	0.0019	0.0001	0.0001	0.0001	0.0001	0.0001	0.0001	0.0001	0.0001	0.0001	0.0001	0.0001	0.0001	0.0001	0.0019	0.0001
440.09	0.0037	0.0046	0.0059	0.0030	0.0054	0.0061	0.0038	0.0038	0.0042	0.0001	0.0047	0.0048	0.0036	0.0030	0.0048	0.0033	0.0038	0.0043	0.0033	0.0056	0.0053
441.14	0.0202	0.0244	0.0274	0.0177	0.0301	0.0313	0.0192	0.0195	0.0215	0.0110	0.0231	0.0236	0.0193	0.0169	0.0231	0.0169	0.0209	0.0216	0.0182	0.0285	0.0283
443.22	0.0034	0.0040	0.0043	0.0023	0.0031	0.0034	0.0035	0.0038	0.0036	0.0001	0.0040	0.0039	0.0026	0.0023	0.0025	0.0035	0.0036	0.0036	0.0031	0.0039	0.0034
444.26	0.0020	0.0022	0.0024	0.0017	0.0001	0.0023	0.0021	0.0020	0.0020	0.0001	0.0022	0.0019	0.0001	0.0001	0.0001	0.0020	0.0001	0.0021	0.0001	0.0025	0.0021
445.25	0.0023	0.0022	0.0021	0.0018	0.0023	0.0023	0.0024	0.0024	0.0021	0.0001	0.0022	0.0001	0.0001	0.0001	0.0001	0.0025	0.0022	0.0021	0.0025	0.0027	0.0023
447.28	0.0036	0.0034	0.0037	0.0024	0.0033	0.0038	0.0051	0.0039	0.0036	0.0001	0.0036	0.0039	0.0090	0.0054	0.0046	0.0048	0.0036	0.0041	0.0032	0.0035	0.0031
448.28	0.0001	0.0001	0.0001	0.0001	0.0001	0.0001	0.0019	0.0001	0.0001	0.0001	0.0001	0.0001	0.0001	0.0001	0.0001	0.0018	0.0001	0.0001	0.0001	0.0001	0.0001
449.22	0.0001	0.0001	0.0001	0.0001	0.0001	0.0001	0.0001	0.0001	0.0001	0.0001	0.0001	0.0001	0.0028	0.0022	0.0022	0.0001	0.0001	0.0001	0.0001	0.0001	0.0001
455.12	0.0061	0.0080	0.0079	0.0048	0.0077	0.0080	0.0068	0.0082	0.0070	0.0041	0.0077	0.0063	0.0057	0.0043	0.0064	0.0064	0.0078	0.0070	0.0062	0.0096	0.0084
456.09	0.0144	0.0179	0.0179	0.0109	0.0181	0.0175	0.0154	0.0182	0.0161	0.0097	0.0172	0.0164	0.0138	0.0122	0.0177	0.0146	0.0173	0.0163	0.0145	0.0214	0.0190
457.20	0.0411	0.0488	0.0472	0.0315	0.0497	0.0468	0.0413	0.0483	0.0458	0.0284	0.0472	0.0475	0.0408	0.0379	0.0494	0.0404	0.0480	0.0460	0.0420	0.0549	0.0513
459.33	0.0049	0.0058	0.0056	0.0039	0.0055	0.0056	0.0050	0.0057	0.0052	0.0001	0.0054	0.0050	0.0044	0.0033	0.0001	0.0050	0.0057	0.0056	0.0050	0.0063	0.0060
460.45	0.0033	0.0036	0.0034	0.0027	0.0037	0.0035	0.0036	0.0036	0.0034	0.0001	0.0035	0.0034	0.0001	0.0029	0.0032	0.0035	0.0036	0.0037	0.0036	0.0039	0.0037
461.40	0.0042	0.0046	0.0039	0.0034	0.0042	0.0039	0.0045	0.0050	0.0041	0.0001	0.0044	0.0027	0.0032	0.0029	0.0027	0.0045	0.0045	0.0043	0.0050	0.0052	0.0047
462.39	0.0026	0.0027	0.0025	0.0022	0.0025	0.0024	0.0029	0.0030	0.0023	0.0001	0.0026	0.0001	0.0024	0.0001	0.0020	0.0030	0.0027	0.0025	0.0030	0.0032	0.0027
463.38	0.0065	0.0066	0.0085	0.0039	0.0049	0.0051	0.0085	0.0052	0.0066	0.0077	0.0064	0.0075	0.0199	0.0168	0.0183	0.0068	0.0055	0.0065	0.0055	0.0054	0.0048
464.45	0.0025	0.0025	0.0029	0.0019	0.0021	0.0020	0.0031	0.0022	0.0024	0.0001	0.0025	0.0001	0.0001	0.0001	0.0001	0.0026	0.0022	0.0024	0.0024	0.0023	0.0020
465.44	0.0024	0.0024	0.0023	0.0018	0.0001	0.0019	0.0026	0.0023	0.0021	0.0001	0.0022	0.0001	0.0035	0.0026	0.0031	0.0025	0.0001	0.0022	0.0024	0.0024	0.0020
473.39	0.0001	0.0018	0.0023	0.0001	0.0001	0.0001	0.0016	0.0001	0.0018	0.0001	0.0024	0.0023	0.0001	0.0001	0.0001	0.0018	0.0001	0.0018	0.0001	0.0001	0.0001
478.23	0.0001	0.0001	0.0017	0.0001	0.0001	0.0001	0.0001	0.0001	0.0001	0.0001	0.0001	0.0001	0.0022	0.0001	0.0021	0.0001	0.0001	0.0001	0.0001	0.0001	0.0001
479.57	0.0083	0.0101	0.0206	0.0001	0.0001	0.0091	0.0088	0.0001	0.0139	0.0001	0.0102	0.0194	0.0302	0.0244	0.0329	0.0077	0.0001	0.0148	0.0001	0.0076	0.0086
494.26	0.0020	0.0018	0.0019	0.0020	0.0001	0.0018	0.0019	0.0021	0.0001	0.0001	0.0027	0.0001	0.0001	0.0001	0.0001	0.0027	0.0001	0.0001	0.0001	0.0024	0.0001
495.36	0.0021	0.0018	0.0018	0.0019	0.0001	0.0001	0.0021	0.0001	0.0001	0.0001	0.0025	0.0001	0.0020	0.0001	0.0001	0.0025	0.0001	0.0001	0.0020	0.0022	0.0001
505.35	0.0001	0.0001	0.0001	0.0001	0.0001	0.0001	0.0001	0.0001	0.0001	0.0001	0.0001	0.0001	0.0001	0.0028	0.0043	0.0001	0.0001	0.0001	0.0001	0.0001	0.0001
511.27	0.0032	0.0019	0.0001	0.0024	0.0034	0.0037	0.0033	0.0043	0.0032	0.0028	0.0040	0.0052	0.0025	0.0019	0.0001	0.0030	0.0043	0.0039	0.0031	0.0028	0.0022
532.97	0.0023	0.0022	0.0025	0.0001	0.0001	0.0001	0.0028	0.0036	0.0021	0.0001	0.0027	0.0025	0.0001	0.0001	0.0001	0.0025	0.0030	0.0024	0.0001	0.0001	0.0001
563.32	0.0115	0.0149	0.0179	0.0021	0.0030	0.0037	0.0120	0.0152	0.0125	0.0101	0.0210	0.0165	0.0001	0.0001	0.0001	0.0115	0.0141	0.0132	0.0074	0.0103	0.0075
571.22	0.0026	0.0058	0.0033	0.0001	0.0025	0.0023	0.0001	0.0037	0.0031	0.0001	0.0026	0.0001	0.0001	0.0074	0.0047	0.0001	0.0001	0.0001	0.0030	0.0032	0.0039
577.31	0.0019	0.0021	0.0027	0.0017	0.0030	0.0028	0.0027	0.0025	0.0019	0.0001	0.0021	0.0019	0.0020	0.0024	0.0023	0.0028	0.0021	0.0018	0.0036	0.0032	0.0030
593.34	0.0033	0.0040	0.0059	0.0015	0.0023	0.0028	0.0040	0.0037	0.0037	0.0001	0.0048	0.0044	0.0021	0.0028	0.0025	0.0034	0.0033	0.0038	0.0036	0.0046	0.0040
609.33	0.0031	0.0025	0.0048	0.0001	0.0001	0.0021	0.0032	0.0021	0.0031	0.0001	0.0028	0.0034	0.0023	0.0028	0.0022	0.0023	0.0001	0.0027	0.0023	0.0022	0.0021
631.23	0.0001	0.0001	0.0001	0.0001	0.0001	0.0001	0.0001	0.0021	0.0027	0.0001	0.0001	0.0019	0.0001	0.0001	0.0001	0.0001	0.0001	0.0023	0.0001	0.0001	0.0001
632.30	0.0001	0.0001	0.0001	0.0001	0.0001	0.0001	0.0001	0.0001	0.0001	0.0001	0.0001	0.0001	0.0032	0.0001	0.0001	0.0015	0.0001	0.0001	0.0001	0.0001	0.0001
633.31	0.0022	0.0030	0.0031	0.0018	0.0025	0.0001	0.0034	0.0021	0.0001	0.0001	0.0054	0.0037	0.0124	0.0025	0.0050	0.0048	0.0035	0.0020	0.0024	0.0028	0.0018
745.19	0.0001	0.0001	0.0001	0.0001	0.0001	0.0001	0.0001	0.0001	0.0001	0.0001	0.0001	0.0001	0.0028	0.0021	0.0034	0.0001	0.0001	0.0001	0.0001	0.0001	0.0001
755.31	0.0117	0.0122	0.0120	0.0037	0.0064	0.0049	0.0145	0.0131	0.0085	0.0103	0.0152	0.0097	0.0001	0.0001	0.0001	0.0150	0.0135	0.0085	0.0092	0.0085	0.0043
756.29	0.0041	0.0044	0.0044	0.0001	0.0001	0.0001	0.0053	0.0047	0.0029	0.0001	0.0055	0.0035	0.0001	0.0001	0.0001	0.0056	0.0048	0.0026	0.0031	0.0001	0.0001
761.19	0.0001	0.0001	0.0001	0.0001	0.0001	0.0001	0.0001	0.0001	0.0001	0.0001	0.0001	0.0001	0.0030	0.0024	0.0038	0.0001	0.0001	0.0001	0.0001	0.0001	0.0001
771.34	0.0196	0.0231	0.0320	0.0043	0.0093	0.0134	0.0184	0.0159	0.0240	0.0167	0.0194	0.0207	0.0045	0.0066	0.0075	0.0145	0.0131	0.0182	0.0089	0.0087	0.0056
772.32	0.0077	0.0091	0.0131	0.0001	0.0001	0.0045	0.0073	0.0058	0.0093	0.0066	0.0070	0.0081	0.0001	0.0001	0.0001	0.0056	0.0047	0.0070	0.0031	0.0027	0.0001
787.43	0.0001	0.0021	0.0026	0.0001	0.0001	0.0001	0.0001	0.0001	0.0020	0.0001	0.0019	0.0001	0.0001	0.0001	0.0001	0.0001	0.0001	0.0001	0.0001	0.0001	0.0001
923.16	0.0001	0.0031	0.0025	0.0001	0.0030	0.0035	0.0001	0.0001	0.0021	0.0001	0.0019	0.0001	0.0001	0.0035	0.0026	0.0001	0.0001	0.0001	0.0001	0.0001	0.0028
924.17	0.0001	0.0018	0.0001	0.0001	0.0001	0.0020	0.0001	0.0001	0.0001	0.0001	0.0001	0.0001	0.0001	0.0001	0.0001	0.0001	0.0001	0.0001	0.0001	0.0001	0.0001
939.15	0.0028	0.0069	0.0055	0.0017	0.0058	0.0065	0.0001	0.0039	0.0056	0.0001	0.0053	0.0033	0.0001	0.0080	0.0065	0.0001	0.0030	0.0029	0.0044	0.0046	0.0067
940.09	0.0001	0.0047	0.0034	0.0001	0.0034	0.0039	0.0001	0.0023	0.0034	0.0001	0.0030	0.0020	0.0001	0.0045	0.0037	0.0001	0.0001	0.0001	0.0026	0.0025	0.0042
968.08	0.0036	0.0042	0.0038	0.0001	0.0045	0.0039	0.0032	0.0043	0.0044	0.0001	0.0057	0.0057	0.0001	0.0001	0.0001	0.0036	0.0043	0.0044	0.0049	0.0065	0.0054
969.06	0.0025	0.0029	0.0026	0.0001	0.0030	0.0025	0.0023	0.0029	0.0029	0.0001	0.0040	0.0041	0.0001	0.0001	0.0001	0.0025	0.0031	0.0033	0.0033	0.0043	0.0037

Supplementary Table S3. The top-40 VIP-valued components highly contributing to the construction of OPLS regression model and their correlations with ORAC values.

			(Name)	AT1	AT2	AT3	BF1	BF2	BF3	KM1	KM2	KM3	SM1	SM2	SM3	SR1	SR2	SR3	YB1	YB2	YB3	YM1	YM2	YM3	Correlation with ORAC	
Variables	VIP	*m/z*	(ORAC)	35,647	38,661	51,482	42,552	65,085	55,612	44,400	43,107	48,544	25,445	40,191	46,160	37,930	42,436	38,953	34,246	36,545	41,729	37,746	43,496	47,566	R^2	(Slope)
V01	3.11	441.14		0.0202	0.0244	0.0274	0.0177	0.0301	0.0313	0.0192	0.0195	0.0215	0.0110	0.0231	0.0236	0.0193	0.0169	0.0231	0.0169	0.0209	0.0216	0.0182	0.0285	0.0283	0.616	(+)
V02	3.06	305.19		0.0407	0.0558	0.0571	0.0422	0.0571	0.0564	0.0484	0.0549	0.0695	0.0465	0.0564	0.0668	0.0336	0.0390	0.0348	0.0407	0.0474	0.0571	0.0508	0.0576	0.0725	0.254	(+)
V03	2.86	289.19		0.0174	0.0226	0.0242	0.0232	0.0291	0.0281	0.0201	0.0224	0.0280	0.0177	0.0213	0.0264	0.0171	0.0207	0.0181	0.0188	0.0203	0.0239	0.0226	0.0243	0.0313	0.615	(+)
V04	2.31	457.20		0.0411	0.0488	0.0472	0.0315	0.0497	0.0468	0.0413	0.0483	0.0458	0.0284	0.0472	0.0475	0.0408	0.0379	0.0494	0.0404	0.0480	0.0460	0.0420	0.0549	0.0513	0.253	(+)
V05	2.26	209.19		0.0085	0.0001	0.0001	0.0096	0.0001	0.0001	0.0076	0.0001	0.0001	0.0087	0.0001	0.0001	0.0085	0.0001	0.0001	0.0075	0.0087	0.0001	0.0078	0.0001	0.0001	0.332	(-)
V06	2.21	287.18		0.0184	0.0246	0.0243	0.0187	0.0253	0.0271	0.0203	0.0237	0.0253	0.0150	0.0235	0.0269	0.0211	0.0202	0.0229	0.0193	0.0224	0.0251	0.0207	0.0261	0.0268	0.487	(+)
V07	2.10	167.02		0.0335	0.0428	0.0393	0.0317	0.0412	0.0405	0.0332	0.0413	0.0419	0.0293	0.0416	0.0425	0.0355	0.0349	0.0389	0.0326	0.0401	0.0414	0.0363	0.0424	0.0440	0.283	(+)
V08	1.99	288.20		0.0094	0.0132	0.0138	0.0097	0.0140	0.0156	0.0101	0.0122	0.0140	0.0073	0.0125	0.0164	0.0101	0.0100	0.0121	0.0096	0.0113	0.0133	0.0104	0.0139	0.0146	0.516	(+)
V09	1.90	183.10		0.0021	0.0001	0.0001	0.0087	0.0090	0.0106	0.0037	0.0038	0.0036	0.0001	0.0001	0.0001	0.0056	0.0067	0.0037	0.0018	0.0021	0.0001	0.0001	0.0001	0.0044	0.296	(+)
V10	1.90	166.02		0.0201	0.0266	0.0267	0.0216	0.0276	0.0264	0.0235	0.0272	0.0331	0.0220	0.0288	0.0288	0.0189	0.0217	0.0198	0.0208	0.0238	0.0272	0.0247	0.0270	0.0332	0.281	(+)
V11	1.83	145.99		0.0107	0.0062	0.0051	0.0114	0.0074	0.0052	0.0119	0.0100	0.0071	0.0150	0.0081	0.0036	0.0096	0.0081	0.0063	0.0115	0.0106	0.0086	0.0129	0.0091	0.0076	0.396	(-)
V12	1.82	177.14		0.0072	0.0001	0.0001	0.0070	0.0001	0.0046	0.0066	0.0001	0.0001	0.0091	0.0001	0.0001	0.0074	0.0073	0.0064	0.0063	0.0001	0.0001	0.0074	0.0001	0.0001	0.242	(-)
V13	1.81	134.99		0.0044	0.0040	0.0001	0.0042	0.0001	0.0001	0.0001	0.0001	0.0001	0.0075	0.0036	0.0037	0.0089	0.0073	0.0049	0.0001	0.0058	0.0053	0.0001	0.0001	0.0001	0.310	(-)
V14	1.78	168.06		0.0359	0.0455	0.0422	0.0336	0.0443	0.0441	0.0349	0.0443	0.0419	0.0311	0.0441	0.0475	0.0424	0.0446	0.0519	0.0349	0.0441	0.0458	0.0382	0.0443	0.0441	0.153	(+)
V15	1.77	144.99		0.0001	0.0001	0.0001	0.0182	0.0083	0.0001	0.0133	0.0082	0.0001	0.0217	0.0001	0.0001	0.0001	0.0001	0.0001	0.0197	0.0001	0.0001	0.0243	0.0001	0.0001	0.105	(-)
V16	1.75	175.44		0.0020	0.0019	0.0017	0.0128	0.0001	0.0019	0.0018	0.0019	0.0017	0.0181	0.0001	0.0022	0.0001	0.0001	0.0001	0.0020	0.0020	0.0021	0.0023	0.0021	0.0001	0.190	(-)
V17	1.72	116.99		0.0066	0.0038	0.0001	0.0080	0.0031	0.0027	0.0051	0.0051	0.0026	0.0073	0.0077	0.0001	0.0071	0.0046	0.0039	0.0051	0.0056	0.0044	0.0060	0.0039	0.0001	0.368	(-)
V18	1.71	128.00		0.0043	0.0018	0.0001	0.0167	0.0064	0.0024	0.0074	0.0061	0.0001	0.0152	0.0034	0.0001	0.0053	0.0061	0.0037	0.0093	0.0055	0.0021	0.0121	0.0030	0.0024	0.182	(-)
V19	1.66	132.97		0.0242	0.0127	0.0062	0.0282	0.0191	0.0129	0.0208	0.0180	0.0078	0.0217	0.0177	0.0053	0.0189	0.0249	0.0175	0.0174	0.0243	0.0112	0.0172	0.0144	0.0076	0.149	(-)
V20	1.57	126.97		0.0085	0.0043	0.0001	0.0068	0.0051	0.0001	0.0066	0.0045	0.0001	0.0072	0.0036	0.0040	0.0065	0.0070	0.0038	0.0061	0.0069	0.0047	0.0073	0.0038	0.0040	0.336	(-)
V21	1.56	136.99		0.0158	0.0199	0.0187	0.0158	0.0220	0.0205	0.0175	0.0210	0.0233	0.0191	0.0181	0.0276	0.0161	0.0200	0.0161	0.0156	0.0208	0.0248	0.0198	0.0194	0.0244	0.162	(+)
V22	1.53	160.13		0.0096	0.0070	0.0042	0.0072	0.0063	0.0044	0.0077	0.0078	0.0050	0.0097	0.0069	0.0001	0.0103	0.0001	0.0053	0.0097	0.0090	0.0057	0.0099	0.0071	0.0056	0.247	(-)
V23	1.51	440.09		0.0037	0.0046	0.0059	0.0030	0.0054	0.0061	0.0038	0.0038	0.0042	0.0001	0.0047	0.0048	0.0036	0.0030	0.0048	0.0033	0.0038	0.0043	0.0033	0.0056	0.0053	0.544	(+)
V24	1.51	271.20		0.0053	0.0056	0.0058	0.0053	0.0068	0.0074	0.0052	0.0052	0.0056	0.0001	0.0052	0.0059	0.0059	0.0058	0.0059	0.0051	0.0053	0.0059	0.0054	0.0061	0.0063	0.494	(+)
V25	1.49	307.24		0.0044	0.0053	0.0050	0.0040	0.0001	0.0001	0.0049	0.0051	0.0001	0.0043	0.0001	0.0001	0.0039	0.0040	0.0037	0.0044	0.0049	0.0052	0.0051	0.0051	0.0001	0.315	(-)
V26	1.48	939.15		0.0028	0.0069	0.0055	0.0017	0.0058	0.0065	0.0001	0.0039	0.0056	0.0001	0.0053	0.0033	0.0001	0.0080	0.0065	0.0001	0.0030	0.0029	0.0044	0.0046	0.0067	0.256	(+)
V27	1.46	157.87		0.0100	0.0096	0.0088	0.0079	0.0107	0.0099	0.0093	0.0100	0.0096	0.0001	0.0111	0.0091	0.0001	0.0103	0.0100	0.0097	0.0110	0.0099	0.0112	0.0109	0.0103	0.170	(+)
V28	1.44	456.09		0.0144	0.0179	0.0179	0.0109	0.0181	0.0175	0.0154	0.0182	0.0161	0.0097	0.0172	0.0164	0.0138	0.0122	0.0177	0.0146	0.0173	0.0163	0.0145	0.0214	0.0190	0.247	(+)
V29	1.36	138.00		0.0092	0.0121	0.0113	0.0093	0.0129	0.0124	0.0103	0.0123	0.0135	0.0105	0.0110	0.0169	0.0084	0.0102	0.0087	0.0090	0.0119	0.0144	0.0111	0.0115	0.0144	0.202	(+)
V30	1.36	162.64		0.0001	0.0058	0.0053	0.0001	0.0059	0.0058	0.0001	0.0058	0.0056	0.0001	0.0058	0.0057	0.0001	0.0001	0.0058	0.0055	0.0001	0.0059	0.0063	0.0061	0.0060	0.180	(+)
V31	1.25	940.09		0.0001	0.0047	0.0034	0.0001	0.0034	0.0039	0.0001	0.0023	0.0034	0.0001	0.0030	0.0020	0.0001	0.0045	0.0037	0.0001	0.0001	0.0001	0.0026	0.0025	0.0042	0.259	(+)
V32	1.25	923.16		0.0001	0.0031	0.0025	0.0001	0.0030	0.0035	0.0001	0.0001	0.0021	0.0001	0.0019	0.0001	0.0001	0.0035	0.0026	0.0001	0.0001	0.0001	0.0001	0.0001	0.0028	0.304	(+)
V33	1.22	125.02		0.0165	0.0201	0.0180	0.0151	0.0212	0.0205	0.0157	0.0189	0.0194	0.0156	0.0175	0.0265	0.0180	0.0186	0.0184	0.0155	0.0215	0.0241	0.0178	0.0194	0.0211	0.161	(+)
V34	1.21	204.14		0.0159	0.0001	0.0001	0.0001	0.0001	0.0001	0.0001	0.0001	0.0001	0.0001	0.0001	0.0001	0.0001	0.0001	0.0001	0.0121	0.0001	0.0001	0.0001	0.0001	0.0001	0.094	(-)
V35	1.15	207.19		0.0038	0.0001	0.0001	0.0001	0.0001	0.0001	0.0001	0.0001	0.0001	0.0054	0.0001	0.0001	0.0001	0.0001	0.0001	0.0001	0.0001	0.0001	0.0001	0.0001	0.0001	0.269	(-)
V36	1.15	304.16		0.0072	0.0097	0.0097	0.0068	0.0089	0.0098	0.0081	0.0091	0.0101	0.0069	0.0088	0.0101	0.0066	0.0064	0.0068	0.0073	0.0079	0.0086	0.0081	0.0097	0.0107	0.319	(+)
V37	1.13	309.20		0.0057	0.0068	0.0061	0.0053	0.0059	0.0061	0.0058	0.0066	0.0067	0.0001	0.0062	0.0061	0.0001	0.0051	0.0050	0.0058	0.0064	0.0066	0.0062	0.0072	0.0070	0.200	(+)
V38	1.09	459.33		0.0049	0.0058	0.0056	0.0039	0.0055	0.0056	0.0050	0.0057	0.0052	0.0001	0.0054	0.0050	0.0044	0.0033	0.0001	0.0050	0.0057	0.0056	0.0050	0.0063	0.0060	0.220	(+)
V39	1.08	150.00		0.0062	0.0068	0.0063	0.0071	0.0092	0.0078	0.0070	0.0074	0.0074	0.0062	0.0061	0.0081	0.0063	0.0075	0.0057	0.0066	0.0075	0.0084	0.0079	0.0071	0.0087	0.368	(+)
V40	1.01	176.01		0.0048	0.0045	0.0001	0.0047	0.0001	0.0032	0.0044	0.0046	0.0001	0.0001	0.0001	0.0001	0.0045	0.0001	0.0040	0.0044	0.0050	0.0001	0.0049	0.0041	0.0001	0.119	(-)

Supplementary Table S4. Combinations of multiple components with the highest VIP values (>1) and their correlation values using three abundance measures.

	Combinations using the Intensity data				R^2
1	V01	V03	V09	V24	0.828
2	V01	V03	V09	V39	0.825
3	V01	V03	V09	V23	0.825
4	V01	V03	V09	-	0.824
5	V01	V03	V09	V38	0.818
6	V03	V08	V09	V23	0.815
7	V01	V03	V09	V32	0.814
8	V03	V09	V23	V24	0.813
9	V01	V03	V08	V09	0.813
10	V01	V09	V29	V39	0.812
11	V01	V03	V09	V36	0.810
12	V01	V09	V36	V39	0.810
13	V01	V03	V09	V37	0.810
14	V01	V08	V09	V39	0.808
15	V01	V09	V29	-	0.807
16	V01	V09	V24	V29	0.807
17	V03	V08	V09	V24	0.807
18	V01	V03	V09	V31	0.806
19	V08	V09	V23	V39	0.804
20	V03	V09	V23	V28	0.804
21	V01	V09	V23	V29	0.802
22	V08	V09	V23	V36	0.802
23	V01	V03	V09	V29	0.802
24	V03	V09	V24	V28	0.801
25	V03	V09	V23	V38	0.801
26	V03	V06	V09	V23	0.800
27	V03	V09	V23	V36	0.799
28	V01	V09	V29	V32	0.799
29	V01	V09	V29	V38	0.798
30	V01	V09	V10	V24	0.798
31	V01	V03	V06	V09	0.797
32	V01	V09	V23	V39	0.796
33	V01	V09	V38	V39	0.796
34	V01	V09	V21	V24	0.795
35	V03	V09	V23	-	0.795
36	V01	V03	V09	V27	0.794
37	V03	V06	V09	V24	0.793
38	V01	V09	V37	V39	0.793
39	V01	V09	V10	V39	0.793
40	V01	V09	V21	V23	0.792
41	V01	V09	V29	V36	0.791
42	V08	V09	V23	V38	0.791
43	V03	V09	V28	-	0.791
44	V03	V09	V28	V32	0.791
45	V01	V09	V39	-	0.790
46	V01	V09	V29	V37	0.790
47	V01	V09	V29	V31	0.790
48	V03	V08	V09	V38	0.789
49	V03	V09	V23	V32	0.789
50	V08	V09	V24	V36	0.788
51	V03	V09	V23	V37	0.788
52	V03	V09	V24	V36	0.787
53	V01	V09	V24	V39	0.787
54	V01	V09	V21	-	0.787
55	V01	V03	V09	V26	0.787
56	V03	V09	V23	V39	0.786
57	V01	V06	V09	V39	0.786
58	V01	V08	V09	V38	0.786
59	V08	V09	V36	V39	0.785
60	V01	V09	V10	-	0.785
61	V01	V08	V09	-	0.784
62	V01	V09	V21	V32	0.784
63	V03	V09	V23	V31	0.784
64	V01	V09	V36	-	0.784
65	V01	V09	V21	V38	0.784
66	V03	V08	V09	V32	0.784
67	V03	V09	V28	V39	0.783
68	V01	V09	V10	V23	0.783
69	V01	V03	V09	V30	0.783
70	V03	V08	V09	-	0.783
71	V01	V09	V24	V36	0.783
72	V06	V09	V23	V39	0.783
73	V08	V09	V32	V38	0.783
74	V01	V08	V09	V29	0.783
75	V01	V03	V09	V28	0.783
76	V01	V09	V31	V39	0.782
77	V03	V08	V09	V28	0.782
78	V03	V06	V09	V32	0.781
79	V09	V10	V23	V24	0.781
80	V01	V09	V21	V39	0.781
81	V01	V08	V09	V36	0.780
82	V03	V06	V09	-	0.780
83	V09	V23	V36	V39	0.780
84	V01	V08	V09	V24	0.779
85	V01	V09	V32	V39	0.779
86	V01	V03	V09	V33	0.778
87	V01	V09	V36	V38	0.778
88	V08	V09	V31	V38	0.777
89	V08	V09	V36	-	0.777
90	V01	V09	V10	V32	0.777
91	V09	V23	V29	V36	0.777
92	V01	V09	V23	V36	0.777
93	V08	V09	V23	-	0.776
94	V01	V08	V09	V37	0.776
95	V01	V03	V09	V21	0.776
96	V03	V06	V09	V38	0.775
97	V01	V09	V21	V37	0.775
98	V08	V09	V36	V38	0.775
99	V01	V09	V32	V38	0.775
100	V01	V08	V09	V32	0.775
101	V03	V08	V09	V37	0.775
102	V01	V09	V31	V38	0.774
103	V03	V09	V24	V38	0.774
104	V01	V08	V09	V23	0.774
105	V09	V23	V24	V29	0.774
106	V01	V09	V36	V37	0.773
107	V03	V09	V28	V38	0.773
108	V08	V09	V23	V29	0.773
109	V03	V08	V09	V39	0.772
110	V08	V09	V23	V37	0.772
111	V01	V09	V30	V39	0.772
112	V01	V09	V38	-	0.771
113	V01	V09	V21	V31	0.771
114	V01	V09	V32	V36	0.771
115	V03	V08	V09	V36	0.771
116	V06	V09	V23	V36	0.770
117	V03	V09	V32	V38	0.770
118	V03	V06	V09	V39	0.770
119	V03	V08	V09	V31	0.770
120	V08	V09	V38	V39	0.770
121	V03	V09	V23	V30	0.769
122	V01	V09	V21	V36	0.769
123	V03	V09	V28	V36	0.768
124	V03	V09	V28	V31	0.768
125	V01	V09	V37	-	0.768
126	V03	V06	V08	V09	0.768
127	V03	V09	V24	V32	0.767
128	V06	V09	V36	V39	0.767
129	V01	V08	V09	V31	0.766
130	V01	V09	V23	V38	0.766
131	V01	V09	V10	V38	0.766
132	V03	V09	V24	V31	0.765
133	V01	V03	V09	V10	0.765
134	V03	V09	V31	V38	0.765
135	V03	V09	V24	V37	0.765
136	V08	V09	V38	-	0.765
137	V08	V09	V23	V24	0.765
138	V01	V06	V09	-	0.765
139	V03	V09	V23	V29	0.765
140	V06	V09	V32	V38	0.765
141	V01	V09	V31	V36	0.765
142	V03	V06	V09	V31	0.764
143	V03	V09	V24	V30	0.764
144	V03	V09	V24	-	0.764
145	V03	V06	V09	V37	0.763
146	V08	V09	V32	V36	0.763
147	V08	V09	V36	V37	0.763
148	V01	V08	V09	V10	0.762
149	V09	V23	V29	V38	0.762
150	V03	V06	V09	V36	0.762
151	V08	V09	V28	V39	0.762
152	V06	V08	V09	V39	0.762
153	V03	V09	V23	V26	0.762
154	V01	V09	V27	V29	0.761
155	V01	V08	V09	V21	0.761
156	V08	V09	V37	V39	0.761
157	V01	V09	V24	V38	0.761
158	V06	V09	V23	-	0.761
159	V01	V06	V09	V38	0.761
160	V01	V06	V09	V29	0.761
161	V01	V06	V09	V32	0.760

	Combinations using the Relative data				R^2
1	V01	V03	V09	V23	0.835
2	V03	V08	V09	V23	0.826
3	V03	V09	V23	-	0.824
4	V01	V02	V09	V24	0.823
5	V01	V09	V23	V39	0.821
6	V02	V09	V23	-	0.821
7	V01	V03	V08	V09	0.820
8	V01	V03	V09	V24	0.819
9	V01	V09	V10	V24	0.817
10	V02	V09	V23	V24	0.815
11	V03	V09	V23	V24	0.814
12	V01	V09	V23	V29	0.813
13	V01	V02	V09	V23	0.813
14	V03	V06	V09	V23	0.813
15	V08	V09	V23	V39	0.813
16	V01	V09	V10	V23	0.812
17	V01	V03	V06	V09	0.810
18	V01	V03	V09	-	0.810
19	V01	V08	V09	V39	0.809
20	V03	V08	V09	V24	0.809
21	V09	V10	V23	-	0.809
22	V01	V09	V21	V23	0.807
23	V03	V09	V23	V28	0.805
24	V01	V02	V09	-	0.805
25	V03	V04	V09	V23	0.804
26	V09	V10	V23	V24	0.804
27	V01	V09	V24	V29	0.803
28	V01	V09	V10	-	0.801
29	V01	V03	V04	V09	0.801
30	V01	V03	V09	V28	0.799
31	V09	V23	V29	-	0.798
32	V01	V08	V09	-	0.796
33	V03	V06	V09	V24	0.794
34	V03	V09	V24	V28	0.794
35	V09	V23	V36	V39	0.793
36	V01	V09	V24	V36	0.793
37	V09	V23	V36	-	0.792
38	V01	V06	V09	V39	0.792
39	V06	V09	V23	V39	0.792
40	V01	V03	V09	V38	0.792
41	V08	V09	V23	-	0.791
42	V01	V09	V29	-	0.791
43	V03	V08	V09	-	0.791
44	V03	V09	V28	-	0.790
45	V03	V09	V23	V29	0.789
46	V03	V04	V09	-	0.789
47	V03	V09	V23	V39	0.789
48	V03	V09	V23	V36	0.788
49	V03	V09	V24	V36	0.787
50	V02	V03	V09	V23	0.787
51	V09	V23	V24	V29	0.787
52	V03	V09	V10	V23	0.787
53	V02	V08	V09	V24	0.787
54	V03	V08	V09	V28	0.787
55	V03	V09	V23	V38	0.786
56	V03	V04	V09	V24	0.786
57	V01	V09	V36	-	0.786
58	V01	V09	V21	V24	0.785
59	V01	V09	V36	V39	0.784
60	V01	V03	V09	V37	0.784
61	V03	V06	V09	-	0.783
62	V01	V08	V09	V10	0.783
63	V01	V08	V09	V23	0.783
64	V01	V03	V09	V27	0.782
65	V08	V09	V10	V24	0.782
66	V03	V04	V08	V09	0.781
67	V01	V08	V09	V24	0.781
68	V01	V03	V09	V29	0.781
69	V09	V23	V24	V36	0.781
70	V08	V09	V10	V23	0.781
71	V02	V08	V09	V23	0.780
72	V01	V03	V09	V36	0.780
73	V09	V23	V28	V39	0.780
74	V01	V02	V08	V09	0.780
75	V01	V03	V09	V10	0.779
76	V01	V03	V09	V39	0.779
77	V09	V24	V36	-	0.778
78	V02	V09	V24	-	0.778
79	V09	V10	V23	V39	0.777
80	V01	V09	V28	V39	0.777
81	V01	V06	V09	-	0.777
82	V01	V02	V03	V09	0.777
83	V03	V07	V09	V23	0.777
84	V02	V09	V23	V39	0.776
85	V01	V09	V23	V36	0.776
86	V01	V08	V09	V38	0.775
87	V03	V09	V23	V33	0.774
88	V01	V03	V07	V09	0.774
89	V03	V08	V09	V38	0.774
90	V02	V03	V09	V24	0.774
91	V03	V09	V23	V37	0.773
92	V03	V06	V08	V09	0.773
93	V01	V04	V09	V39	0.771
94	V01	V09	V10	V39	0.771
95	V04	V09	V23	V39	0.771
96	V03	V09	V21	V23	0.770
97	V01	V03	V09	V33	0.770
98	V03	V09	V23	V27	0.770
99	V01	V03	V09	V14	0.770
100	V03	V09	V10	V24	0.770
101	V01	V02	V06	V09	0.770
102	V08	V09	V24	V36	0.769
103	V02	V06	V09	V24	0.769
104	V09	V21	V23	V24	0.769
105	V01	V06	V09	V10	0.768
106	V02	V06	V09	V23	0.768
107	V01	V08	V09	V29	0.767
108	V01	V09	V24	V39	0.767
109	V06	V09	V23	-	0.767
110	V03	V09	V14	V23	0.766
111	V03	V06	V09	V28	0.766
112	V01	V02	V09	V39	0.766
113	V03	V08	V09	V27	0.766
114	V08	V09	V28	V39	0.765
115	V09	V21	V23	-	0.765
116	V01	V06	V09	V23	0.765
117	V02	V09	V24	V28	0.765
118	V01	V06	V08	V09	0.763
119	V08	V09	V23	V29	0.763
120	V01	V03	V09	V21	0.763
121	V06	V09	V10	V23	0.763
122	V01	V08	V09	V37	0.763
123	V01	V09	V21	-	0.761
124	V08	V09	V23	V24	0.761
125	V03	V07	V09	V24	0.761
126	V03	V04	V06	V09	0.761
127	V06	V09	V10	V24	0.760
128	V01	V06	V09	V24	0.759
129	V09	V10	V24	-	0.759
130	V01	V06	V09	V29	0.759
131	V08	V09	V24	V39	0.758
132	V01	V08	V09	V36	0.758
133	V01	V09	V23	-	0.758
134	V03	V09	V24	V29	0.758
135	V09	V24	V36	V39	0.758
136	V02	V04	V09	V24	0.757
137	V03	V08	V09	V37	0.757
138	V01	V08	V09	V27	0.757
139	V08	V09	V23	V38	0.756
140	V03	V06	V09	V38	0.756
141	V01	V09	V28	V29	0.755
142	V01	V03	V24	-	0.755
143	V09	V23	V24	V39	0.754
144	V08	V09	V23	V36	0.754
145	V01	V08	V09	V21	0.754
146	V01	V07	V09	V39	0.753
147	V03	V09	V14	V38	0.753
148	V07	V09	V23	V39	0.753
149	V06	V08	V09	V23	0.753
150	V06	V08	V09	-	0.752
151	V01	V02	V04	V09	0.752
152	V04	V08	V09	V39	0.752
153	V06	V09	V23	V29	0.752
154	V03	V06	V09	V27	0.752
155	V06	V08	V09	V39	0.752
156	V01	V02	V09	V27	0.752
157	V09	V23	V28	V29	0.752
158	V03	V09	V24	-	0.752
159	V09	V10	V24	V28	0.750
160	V06	V09	V24	V36	0.750
161	V02	V04	V09	V23	0.750

	Combinations using the Score data				R^2
1	V03	V09	V23	-	0.812
2	V03	V08	V09	V23	0.803
3	V01	V03	V08	V09	0.794
4	V01	V03	V09	-	0.793
5	V02	V03	V09	V23	0.792
6	V01	V03	V09	V23	0.787
7	V01	V02	V03	V09	0.784
8	V03	V09	V10	V23	0.783
9	V02	V09	V23	-	0.782
10	V03	V06	V09	V23	0.781
11	V03	V08	V09	-	0.780
12	V03	V09	V23	V39	0.778
13	V01	V03	V09	V10	0.774
14	V01	V02	V09	-	0.772
15	V01	V09	V23	V39	0.772
16	V01	V03	V06	V09	0.772
17	V03	V09	V23	V24	0.771
18	V02	V09	V23	V39	0.771
19	V08	V09	V23	V39	0.770
20	V03	V09	V23	V29	0.767
21	V02	V03	V09	V24	0.765
22	V09	V10	V23	-	0.758
23	V02	V08	V09	-	0.757
24	V01	V03	V09	V39	0.757
25	V03	V09	V10	V24	0.756
26	V03	V09	V23	V36	0.756
27	V09	V10	V23	V24	0.756
28	V02	V09	V23	V24	0.755
29	V01	V02	V09	V39	0.754
30	V03	V08	V09	V24	0.754
31	V03	V06	V08	V09	0.754
32	V02	V03	V08	V09	0.754
33	V02	V08	V09	V23	0.753
34	V01	V08	V09	V39	0.753
35	V03	V06	V09	-	0.753
36	V01	V03	V09	V29	0.752
37	V01	V03	V09	V36	0.751
38	V01	V02	V08	V09	0.751
39	V01	V03	V09	V24	0.750

Among 15,250 combinations of 2−4 components, the representative combinations with higher correlation values (R^2>0.75) are shown.

(Continued)

	Combinations using the Intensity data				R^2
162	V03	V08	V09	V27	0.760
163	V06	V09	V24	V36	0.760
164	V01	V09	V26	V29	0.760
165	V01	V09	V32	V37	0.760
166	V01	V09	V23	V37	0.760
167	V06	V09	V36	-	0.760
168	V01	V09	V23	-	0.760
169	V03	V09	V23	V27	0.759
170	V01	V06	V09	V24	0.759
171	V01	V09	V10	V31	0.759
172	V08	V09	V23	V32	0.759
173	V01	V06	V09	V36	0.758
174	V09	V23	V29	V31	0.758
175	V06	V09	V23	V38	0.757
176	V08	V09	V24	V29	0.757
177	V08	V09	V24	V39	0.757
178	V09	V23	V29	V32	0.757
179	V01	V09	V24	V37	0.757
180	V01	V09	V10	V37	0.756
181	V03	V23	V24	V32	0.756
182	V03	V09	V24	V39	0.755
183	V08	V09	V23	V31	0.755
184	V03	V06	V09	V28	0.755
185	V01	V09	V24	-	0.755
186	V09	V23	V24	V36	0.755
187	V01	V09	V37	V38	0.754
188	V06	V09	V32	V36	0.754
189	V01	V06	V09	V23	0.754
190	V01	V09	V29	V30	0.754
191	V01	V09	V23	V24	0.754
192	V09	V23	V36	V38	0.754
193	V08	V09	V24	V38	0.754
194	V01	V09	V26	V39	0.754
195	V06	V08	V09	V23	0.753
196	V01	V06	V09	V37	0.753
197	V09	V23	V29	-	0.753
198	V01	V09	V27	V39	0.753
199	V01	V09	V21	V27	0.753
200	V01	V09	V10	V36	0.753
201	V06	V09	V23	V32	0.753
202	V03	V09	V28	V37	0.752
203	V01	V09	-	-	0.752
204	V03	V09	V24	V29	0.752
205	V01	V09	V31	V37	0.752
206	V03	V08	V09	V26	0.752
207	V08	V09	V37	-	0.752
208	V09	V24	V29	V36	0.752
209	V09	V23	V29	V37	0.751
210	V01	V08	V09	V27	0.751
211	V01	V03	V24	V39	0.751
212	V09	V23	V36	V37	0.751
213	V08	V09	V10	V24	0.751
214	V03	V09	V32	V36	0.751
215	V01	V09	V23	V31	0.751
216	V03	V09	V26	V38	0.751
217	V01	V09	V24	V31	0.751
218	V01	V09	V23	V32	0.751
219	V03	V06	V09	V27	0.750
220	V01	V09	V30	V38	0.750
221	V01	V06	V08	V09	0.750
222	V06	V09	V31	V38	0.750
223	V06	V09	V38	V39	0.750

Combinations of the top-4 VIP components				R^2 (Intensity)	R^2 (Relative)	R^2 (Score)
V01	V02	V03	V04	0.486	0.561	0.510
V01	V02	V03	-	0.493	0.589	0.560
V01	V02	V04	-	0.433	0.473	0.416
V01	V03	V04	-	0.567	0.626	0.530
V02	V03	V04	-	0.403	0.479	0.472
V01	V02	-	-	0.439	0.524	0.478
V01	V03	-	-	0.721	0.722	0.643
V01	V04	-	-	0.434	0.461	0.365
V02	V03	-	-	0.358	0.451	0.487
V02	V04	-	-	0.325	0.330	0.336
V03	V04	-	-	0.493	0.573	0.503

Top 25 VIP	R^2 (Intensity)	R^2 (Relative)	R^2 (Score)	Slope
V01	0.616	0.616	0.483	(+)
V02	0.254	0.254	0.326	(+)
V03	0.615	0.615	0.602	(+)
V04	0.253	0.253	0.209	(+)
V06	0.487	0.487	0.481	(+)
V07	0.283	0.283	0.176	(+)
V08	0.516	0.516	0.544	(+)
V09	0.296	0.296	0.173	(+)
V10	0.281	0.281	0.271	(+)
V14	0.153	0.153	0.063	(+)
V21	0.162	0.162	0.201	(+)
V23	0.544	0.544	0.468	(+)
V24	0.494	0.494	0.426	(+)
V26	0.256	0.256	0.252	(+)
V27	0.170	0.170	0.024	(+)
V28	0.247	0.247	0.273	(+)
V29	0.202	0.202	0.276	(+)
V30	0.180	0.180	0.182	(+)
V31	0.259	0.259	0.264	(+)
V32	0.304	0.304	0.297	(+)
V33	0.161	0.161	0.235	(+)
V36	0.319	0.319	0.291	(+)
V37	0.200	0.200	0.068	(+)
V38	0.220	0.220	0.137	(+)
V39	0.368	0.368	0.316	(+)

These data are showing correlation values of positively correlated components (Slope >0) among the top-40 components with large VIP values (>1).

Supplementary Table S5. LC–MS datasets of 21 GTEs for multivariate statistical analyses.

			(Name)	AT1	AT2	AT3	BF1	BF2	BF3	KM1	KM2	KM3	SM1	SM2	SM3	SR1	SR2	SR3	YB1	YB2	YB3	YM1	YM2	YM3
			(ORAC)	35,647	38,661	51,482	42,552	65,085	55,612	44,400	43,107	48,544	25,445	40,191	46,160	37,930	42,436	38,953	34,246	36,545	41,729	37,746	43,496	47,566
m/z	R.T.	Polar	(Polyphenol)	1.93	2.11	2.54	2.04	2.56	2.54	2.00	2.11	2.41	1.96	2.13	2.33	2.44	2.33	2.17	2.12	2.13	2.34	2.21	2.28	2.65
125.03	8.909	-		0.00007	0.00006	0.00008	0.00014	0.00009	0.00008	0.00002	0.00002	0.00008	0.00000	0.00003	0.00007	0.00004	0.00005	0.00002	0.00004	0.00001	0.00005	0.00005	0.00009	0.00005
133.02	2.142	-		0.00051	0.00023	0.00013	0.00044	0.00018	0.00017	0.00049	0.00028	0.00012	0.00027	0.00031	0.00011	0.00012	0.00021	0.00013	0.00055	0.00024	0.00004	0.00043	0.00031	0.00006
135.03	2.325	-		0.00011	0.00005	0.00005	0.00003	0.00000	0.00000	0.00013	0.00004	0.00002	0.00009	0.00009	0.00005	0.00006	0.00002	0.00003	0.00009	0.00003	0.00001	0.00009	0.00006	0.00000
137.03	8.56	-		0.00003	0.00001	0.00006	0.00000	0.00004	0.00003	0.00002	0.00003	0.00007	0.00004	0.00002	0.00003	0.00000	0.00001	0.00000	0.00004	0.00001	0.00003	0.00006	0.00006	0.00007
139.04	8.917	+		0.00302	0.00400	0.00406	0.00363	0.00329	0.00342	0.00318	0.00363	0.00421	0.00314	0.00392	0.00432	0.00364	0.00295	0.00359	0.00374	0.00425	0.00446	0.00325	0.00400	0.00452
146.96	13.209	-		0.00003	0.00005	0.00000	0.00002	0.00003	0.00007	0.00002	0.00000	0.00004	0.00004	0.00005	0.00008	0.00003	0.00001	0.00003	0.00003	0.00000	0.00005	0.00005	0.00002	0.00003
147.04	9.514	+		0.00127	0.00000	0.00000	0.00218	0.00279	0.00100	0.00197	0.00149	0.00100	0.00240	0.00172	0.00190	0.00193	0.00135	0.00000	0.00343	0.00264	0.00201	0.00164	0.00109	0.00112
154.97	13.759	-		0.00004	0.00002	0.00008	0.00003	0.00002	0.00003	0.00004	0.00001	0.00001	0.00004	0.00004	0.00002	0.00003	0.00004	0.00003	0.00001	0.00002	0.00001	0.00003	0.00005	0.00002
158.08	2.173	+		0.00400	0.00178	0.00093	0.00480	0.00221	0.00147	0.00433	0.00294	0.00092	0.00489	0.00247	0.00148	0.00172	0.00192	0.00172	0.00468	0.00266	0.00058	0.00446	0.00255	0.00122
163.08	12.362	+		0.00000	0.00024	0.00000	0.00000	0.00011	0.00025	0.00000	0.00011	0.00029	0.00000	0.00029	0.00024	0.00011	0.00000	0.00012	0.00000	0.00015	0.00028	0.00015	0.00041	0.00031
163.13	11.066	+		0.00152	0.00125	0.00138	0.00138	0.00197	0.00089	0.00118	0.00193	0.00137	0.00119	0.00178	0.00115	0.00154	0.00163	0.00145	0.00166	0.00149	0.00161	0.00131	0.00095	0.00155
165.02	8.481	-		0.00001	0.00001	0.00001	0.00003	0.00002	0.00003	0.00000	0.00003	0.00001	0.00004	0.00000	0.00004	0.00000	0.00000	0.00000	0.00000	0.00001	0.00001	0.00004	0.00004	0.00003
166.03	8.481	-		0.00007	0.00009	0.00009	0.00008	0.00009	0.00008	0.00010	0.00009	0.00011	0.00009	0.00009	0.00010	0.00004	0.00004	0.00003	0.00009	0.00007	0.00011	0.00011	0.00008	0.00011
166.08	6.24	+		0.00013	0.00054	0.00000	0.00011	0.00070	0.00010	0.00000	0.00033	0.00176	0.00019	0.00000	0.00048	0.00000	0.00066	0.00000	0.00041	0.00119	0.00128	0.00000	0.00011	0.00233
169.02	4.749	-		0.00000	0.00001	0.00001	0.00000	0.00001	0.00000	0.00002	0.00001	0.00000	0.00001	0.00007	0.00009	0.00006	0.00002	0.00006	0.00002	0.00002	0.00004	0.00000	0.00000	0.00000
169.02	8.906	-		0.00034	0.00037	0.00039	0.00037	0.00041	0.00029	0.00027	0.00033	0.00037	0.00027	0.00040	0.00046	0.00034	0.00031	0.00031	0.00034	0.00039	0.00042	0.00031	0.00042	0.00039
169.05	8.493	+		0.00023	0.00034	0.00031	0.00007	0.00024	0.00010	0.00000	0.00016	0.00000	0.00022	0.00011	0.00018	0.00004	0.00020	0.00004	0.00024	0.00026	0.00008	0.00012	0.00014	0.00012
171.07	10.651	-		0.00002	0.00001	0.00002	0.00002	0.00002	0.00001	0.00003	0.00002	0.00000	0.00004	0.00001	0.00021	0.00000	0.00003	0.00002	0.00001	0.00001	0.00000	0.00000	0.00000	0.00001
173.04	2.13	-		0.00012	0.00002	0.00001	0.00011	0.00003	0.00003	0.00011	0.00002	0.00004	0.00009	0.00000	0.00000	0.00001	0.00003	0.00005	0.00010	0.00003	0.00002	0.00013	0.00002	0.00001
173.05	2.112	-		0.00008	0.00009	0.00004	0.00000	0.00000	0.00003	0.00000	0.00011	0.00002	0.00003	0.00004	0.00007	0.00004	0.00013	0.00007	0.00003	0.00000	0.00007	0.00004	0.00003	0.00005
173.05	9.565	-		0.00003	0.00000	0.00000	0.00008	0.00013	0.00002	0.00007	0.00003	0.00001	0.00011	0.00006	0.00005	0.00004	0.00001	0.00000	0.00017	0.00009	0.00006	0.00004	0.00001	0.00001
173.10	2.166	-		0.00134	0.00048	0.00018	0.00210	0.00085	0.00046	0.00148	0.00084	0.00022	0.00200	0.00080	0.00045	0.00056	0.00066	0.00046	0.00164	0.00085	0.00026	0.00164	0.00079	0.00036
173.22	2.161	-		0.00012	0.00000	0.00000	0.00035	0.00005	0.00000	0.00018	0.00003	0.00000	0.00021	0.00003	0.00000	0.00001	0.00004	0.00000	0.00021	0.00006	0.00000	0.00012	0.00004	0.00000
175.03	2.07	-		0.00069	0.00000	0.00000	0.00043	0.00000	0.00000	0.00054	0.00000	0.00000	0.00122	0.00000	0.00000	0.00000	0.00000	0.00000	0.00085	0.00000	0.00000	0.00123	0.00000	0.00000
175.11	2.169	+		0.02333	0.01038	0.00565	0.02800	0.01320	0.00865	0.02470	0.01690	0.00634	0.02769	0.01567	0.00914	0.01060	0.01285	0.01050	0.02670	0.01630	0.00533	0.02540	0.01580	0.00768
180.90	15.052	-		0.00002	0.00002	0.00002	0.00002	0.00001	0.00001	0.00003	0.00000	0.00004	0.00002	0.00002	0.00003	0.00000	0.00001	0.00003	0.00003	0.00000	0.00006	0.00002	0.00000	0.00001
180.90	13.334	-		0.00002	0.00002	0.00000	0.00002	0.00000	0.00002	0.00001	0.00000	0.00000	0.00000	0.00003	0.00005	0.00002	0.00003	0.00002	0.00000	0.00002	0.00000	0.00002	0.00002	0.00000
180.98	13.566	-		0.00005	0.00011	0.00003	0.00004	0.00007	0.00002	0.00003	0.00005	0.00005	0.00006	0.00012	0.00009	0.00004	0.00006	0.00007	0.00004	0.00003	0.00011	0.00007	0.00004	0.00006
181.07	8.027	+		0.00153	0.00057	0.00086	0.00274	0.00189	0.00265	0.00102	0.00055	0.00069	0.00057	0.00049	0.00062	0.00143	0.00116	0.00171	0.00100	0.00045	0.00043	0.00068	0.00077	0.00079
181.99	2.513	+		0.00000	0.00022	0.00012	0.00000	0.00009	0.00000	0.00000	0.00000	0.00030	0.00000	0.00010	0.00000	0.00000	0.00000	0.00000	0.00000	0.00020	0.00000	0.00000	0.00014	0.00038
185.12	11.07	+		0.00052	0.00044	0.00048	0.00056	0.00061	0.00034	0.00029	0.00063	0.00048	0.00045	0.00055	0.00041	0.00047	0.00050	0.00046	0.00050	0.00049	0.00049	0.00046	0.00032	0.00050
186.22	9.172	+		0.00089	0.00128	0.00087	0.00101	0.00094	0.00045	0.00085	0.00082	0.00080	0.00088	0.00078	0.00081	0.00064	0.00101	0.00045	0.00065	0.00085	0.00097	0.00114	0.00069	0.00046
187.10	11.019	-		0.00002	0.00004	0.00002	0.00001	0.00001	0.00001	0.00008	0.00002	0.00000	0.00002	0.00007	0.00003	0.00001	0.00002	0.00001	0.00000	0.00002	0.00002	0.00001	0.00002	0.00002
188.07	8.093	+		0.00017	0.00034	0.00032	0.00028	0.00073	0.00028	0.00029	0.00064	0.00039	0.00020	0.00030	0.00025	0.00045	0.00045	0.00036	0.00047	0.00070	0.00035	0.00014	0.00024	0.00039
190.93	5.207	-		0.00006	0.00008	0.00007	0.00005	0.00005	0.00010	0.00011	0.00004	0.00003	0.00006	0.00003	0.00013	0.00007	0.00005	0.00009	0.00003	0.00001	0.00025	0.00006	0.00012	0.00004
190.93	7.06	-		0.00007	0.00008	0.00005	0.00006	0.00008	0.00012	0.00011	0.00009	0.00011	0.00006	0.00011	0.00010	0.00007	0.00006	0.00010	0.00004	0.00009	0.00011	0.00012	0.00016	0.00006
190.93	11.557	-		0.00016	0.00018	0.00008	0.00009	0.00005	0.00010	0.00021	0.00018	0.00020	0.00014	0.00007	0.00010	0.00017	0.00011	0.00014	0.00027	0.00021	0.00026	0.00022	0.00019	0.00014
190.93	16.183	-		0.00008	0.00008	0.00009	0.00008	0.00017	0.00011	0.00008	0.00009	0.00012	0.00001	0.00008	0.00008	0.00011	0.00010	0.00015	0.00024	0.00063	0.00008	0.00016	0.00004	0.00011
190.93	4.443	-		0.00009	0.00005	0.00012	0.00008	0.00007	0.00004	0.00012	0.00011	0.00008	0.00010	0.00007	0.00021	0.00013	0.00005	0.00009	0.00007	0.00003	0.00031	0.00012	0.00007	0.00010
190.93	2.592	-		0.00008	0.00013	0.00010	0.00022	0.00023	0.00025	0.00054	0.00023	0.00009	0.00053	0.00030	0.00034	0.00007	0.00024	0.00018	0.00028	0.00005	0.00033	0.00052	0.00034	0.00038
190.93	3.785	-		0.00010	0.00009	0.00009	0.00020	0.00024	0.00013	0.00001	0.00000	0.00009	0.00013	0.00005	0.00002	0.00013	0.00007	0.00003	0.00004	0.00008	0.00001	0.00018	0.00008	0.00013
190.94	2.392	-		0.00009	0.00011	0.00013	0.00053	0.00018	0.00014	0.00087	0.00081	0.00012	0.00012	0.00035	0.00070	0.00010	0.00022	0.00048	0.00013	0.00002	0.00020	0.00024	0.00002	0.00002
190.97	2.343	-		0.00001	0.00000	0.00002	0.00002	0.00001	0.00000	0.00001	0.00008	0.00030	0.00016	0.00011	0.00010	0.00000	0.00002	0.00013	0.00025	0.00000	0.00002	0.00002	0.00000	0.00002
191.01	2.157	-		0.00000	0.00000	0.00023	0.00000	0.00028	0.00030	0.00037	0.00000	0.00054	0.00008	0.00000	0.00000	0.00037	0.00043	0.00023	0.00000	0.00008	0.00022	0.00000	0.00019	0.00029
191.03	2.388	-		0.00027	0.00044	0.00038	0.00150	0.00040	0.00059	0.00102	0.00077	0.00019	0.00112	0.00083	0.00040	0.00025	0.00028	0.00038	0.00116	0.00047	0.00021	0.00117	0.00065	0.00035
191.06	9.555	-		0.00003	0.00002	0.00002	0.00002	0.00004	0.00002	0.00001	0.00002	0.00002	0.00003	0.00003	0.00002	0.00003	0.00003	0.00000	0.00003	0.00002	0.00003	0.00006	0.00001	0.00000
191.06	1.935	-		0.00399	0.00232	0.00254	0.00668	0.00584	0.00541	0.00510	0.00420	0.00419	0.00439	0.00308	0.00302	0.00391	0.00597	0.00426	0.00651	0.00402	0.00288	0.00727	0.00370	0.00416
192.92	7.431	-		0.00001	0.00000	0.00000	0.00001	0.00002	0.00000	0.00000	0.00000	0.00000	0.00000	0.00000	0.00001	0.00001	0.00002	0.00000	0.00000	0.00000	0.00000	0.00001	0.00000	0.00000
194.98	11.363	-		0.00002	0.00002	0.00005	0.00005	0.00004	0.00004	0.00002	0.00005	0.00004	0.00003	0.00002	0.00005	0.00004	0.00003	0.00001	0.00006	0.00004	0.00002	0.00006	0.00003	0.00003
194.98	12.783	-		0.00001	0.00000	0.00003	0.00000	0.00002	0.00000	0.00001	0.00003	0.00000	0.00002	0.00002	0.00000	0.00000	0.00001	0.00003	0.00003	0.00000	0.00003	0.00000	0.00000	0.00001
194.99	8.669	-		0.00004	0.00003	0.00005	0.00001	0.00001	0.00004	0.00000	0.00006	0.00008	0.00008	0.00007	0.00002	0.00004	0.00003	0.00003	0.00004	0.00013	0.00010	0.00005	0.00005	0.00003
195.81	2.152	-		0.00001	0.00026	0.00034	0.00044	0.00054	0.00043	0.00000	0.00021	0.00086	0.00018	0.00031	0.00056	0.00050	0.00039	0.00055	0.00000	0.00044	0.00071	0.00021	0.00008	0.00071
196.03	10.499	-		0.00000	0.00000	0.00001	0.00000	0.00000	0.00000	0.00001	0.00000	0.00000	0.00000	0.00000	0.00016	0.00000	0.00000	0.00000	0.00000	0.00000	0.00000	0.00000	0.00000	0.00000
196.88	11.993	-		0.00011	0.00000	0.00003	0.00018	0.00007	0.00000	0.00007	0.00009	0.00013	0.00008	0.00004	0.00000	0.00022	0.00000	0.00007	0.00007	0.00013	0.00000	0.00015	0.00008	0.00018
196.99	11.998	-		0.00008	0.00020	0.00026	0.00024	0.00006	0.00000	0.00013	0.00011	0.00026	0.00027	0.00030	0.00027	0.00022	0.00020	0.00029	0.00022	0.00038	0.00042	0.00021	0.00028	0.00005
197.18	11.996	-		0.00085	0.00072	0.00067	0.00077	0.00062	0.00060	0.00061	0.00044	0.00072	0.00067	0.00078	0.00067	0.00077	0.00066	0.00070	0.00072	0.00067	0.00071	0.00069	0.00072	0.00064
197.33	11.997	-		0.00077	0.00059	0.00020	0.00067	0.00052	0.00027	0.00020	0.00022	0.00035	0.00021	0.00023	0.00021	0.00044	0.00031	0.00023	0.00022	0.00064	0.00010	0.00048	0.00046	0.00010
197.81	2.147	-		0.00004	0.00025	0.00043	0.00060	0.00073	0.00051	0.00002	0.00031	0.00107	0.00025	0.00039	0.00069	0.00063	0.00047	0.00070	0.00000	0.00057	0.00102	0.00024	0.00010	0.00095
197.89	13.67	-		0.00001	0.00000	0.00000	0.00001	0.00000	0.00000	0.00001	0.00000	0.00003	0.00001	0.00001	0.00000	0.00002	0.00001	0.00000	0.00001	0.00000	0.00000	0.00000	0.00001	0.00000
199.17	16.307	-		0.00003	0.00003	0.00002	0.00001	0.00002	0.00000	0.00003	0.00002	0.00000	0.00002	0.00003	0.00027	0.00001	0.00003	0.00000	0.00000	0.00001	0.00002	0.00000	0.00001	0.00001
199.81	2.148	-		0.00000	0.00013	0.00019	0.00032	0.00038	0.00033	0.00001	0.00013	0.00048	0.00007	0.00022	0.00037	0.00025	0.00027	0.00036	0.00000	0.00023	0.00046	0.00000	0.00002	0.00043
203.02	2.836	-		0.00011	0.00013	0.00019	0.00009	0.00008	0.00005	0.00014	0.00015	0.00014	0.00011	0.00018	0.00012	0.00008	0.00007	0.00005	0.00016	0.00013	0.00012	0.00011	0.00015	0.00012
203.08	8.077	-		0.00002	0.00009	0.00003	0.00006	0.00012	0.00000	0.00005	0.00010	0.00003	0.00004	0.00006	0.00003	0.00002	0.00001	0.00001	0.00013	0.00020	0.00005	0.00000	0.00003	0.00006
203.10	13.789	+		0.00227	0.00025	0.00217	0.00208	0.00046	0.00155	0.00406	0.00257	0.00084	0.00416	0.00113	0.00266	0.00034	0.00215	0.00000	0.00072	0.00220	0.00020	0.00046	0.00200	0.00058
203.11	14.744	+		0.00055	0.00052	0.00069	0.00000	0.00037	0.00119	0.00034	0.00088	0.00089	0.00028	0.00016	0.00084	0.00101	0.00100	0.00090	0.00102	0.00049	0.00037	0.00097	0.00113	0.00015
205.10	8.092	+		0.00051	0.00100	0.00084	0.00060	0.00168	0.00067	0.00088	0.00159	0.00086	0.00046	0.00082	0.00063	0.00104	0.00111	0.00093	0.00110	0.00177	0.00093	0.00027	0.00058	0.00096
209.06	2.053	-		0.00004	0.00000	0.00000	0.00015	0.00002	0.00000	0.00000	0.00003	0.00000	0.00007	0.00000	0.00000	0.00004	0.00000	0.00000	0.00005	0.00000	0.00000	0.00000	0.00000	0.00000
219.17	15.482	+		0.00173	0.00219	0.00181	0.00230	0.00196	0.00176	0.00212	0.00211	0.00222	0.00209	0.00184	0.00213	0.00183	0.00209	0.00191	0.00240	0.00192	0.00237	0.00187	0.00179	0.00188
221.08	12.506	-		0.00062	0.00033	0.00058	0.00104	0.00083	0.00081	0.00057	0.00080	0.00061	0.00049	0.00087	0.00068	0.00055	0.00075	0.00043	0.00067	0.00044	0.00025	0.00064	0.00056	0.00043
221.14	16.737	+		0.00177	0.00138	0.00155	0.00202	0.00065	0.00166	0.00105	0.00094	0.00202	0.00108	0.00134	0.00165	0.00221	0.00189	0.00041	0.00198	0.00090	0.00110	0.00025	0.00216	0.00235
221.15	13.642	-		0.00169	0.00013	0.00153	0.00168	0.00038	0.00151	0.00302	0.00173	0.00060	0.00286	0.00075	0.00187	0.00016	0.00156	0.00022	0.00048	0.00151	0.00000	0.00047	0.00129	0.00032
225.15	13.163	+		0.00000	0.00033	0.00000	0.00000	0.00021	0.00008	0.00000	0.00021	0.00016	0.00000	0.00000	0.00009	0.00009	0.00013	0.00010	0.00016	0.00014	0.00007	0.00014	0.00020	0.00031
226.18	12.655	+		0.00274	0.00060	0.00197	0.00278	0.00152	0.00283	0.00204	0.00285	0.00053	0.00256	0.00112	0.00037	0.00027	0.00191	0.00036	0.00060	0.00272	0.00057	0.00099	0.00250	0.00215
227.20	17.127	-		0.00021	0.00031	0.00028	0.00026	0.00022	0.00027	0.00042	0.00025	0.00024	0.00026	0.00030	0.00104	0.00024	0.00023	0.00017	0.00024	0.00025	0.00024	0.00027	0.00020	0.00028
229.19	12.769	+		0.00181	0.00018	0.00139	0.00165	0.00072	0.00183	0.00088	0.00115	0.00010	0.00171	0.00030	0.00000	0.00003	0.00113	0.00013	0.00011	0.00179	0.00026	0.00042	0.00183	0.00164
231.93	5.343	-		0.00001	0.00004	0.00004	0.00003	0.00003	0.00002	0.00004	0.00001	0.00002	0.00000	0.00002	0.00006	0.00003	0.00005	0.00004	0.00002	0.00005	0.00005	0.00001	0.00002	0.00003
231.93	3.957	-		0.00005	0.00007	0.00005	0.00006	0.00002	0.00004	0.00003	0.00005	0.00007	0.00006	0.00002	0.00004	0.00005	0.00005	0.00001	0.00003	0.00003	0.00013	0.00004	0.00004	0.00001
231.93	6.921	-		0.00005	0.00010	0.00010	0.00006	0.00005	0.00004	0.00006	0.00005	0.00008	0.00005	0.00003	0.00004	0.00001	0.00002	0.00005	0.00006	0.00009	0.00004	0.00003	0.00006	0.00006
233.15	14.789	-		0.00005	0.00005	0.00004	0.00007	0.00006	0.00005	0.00004	0.00005	0.00005	0.00005	0.00003	0.00004	0.00002	0.00004	0.00004	0.00005	0.00005	0.00004	0.00006	0.00003	0.00004
235.17	14.789	+		0.00040	0.00036	0.00028	0.00042	0.00038	0.00035	0.00038	0.00042	0.00042	0.00035	0.00039	0.00041	0.00038	0.00041	0.00044	0.00042	0.00039	0.00037	0.00045	0.00038	0.00040
235.92	5.108	-		0.00001	0.00000	0.00007	0.00000	0.00001	0.00003	0.00001	0.00002	0.00007	0.00003	0.00000	0.00002	0.00004	0.00000	0.00000	0.00003	0.00002	0.00003	0.00002	0.00004	0.00005
236.11	13.642	-		0.00179	0.00017	0.00149	0.00154	0.00036	0.00149	0.00305	0.00170	0.00049	0.00286	0.00067	0.00190	0.00013	0.00158	0.00018	0.00043	0.00148	0.00003	0.00041	0.00124	0.00029
237.08	7.85	+		0.00014	0.00021	0.00032	0.00000	0.00007	0.00021	0.00000	0.00000	0.00000	0.00019	0.00000	0.00000	0.00000	0.00011	0.00000	0.00000	0.00019	0.00000	0.00000	0.00016	0.00041
239.13	13.526	-		0.00027	0.00027	0.00040	0.00024	0.00033	0.00024	0.00046	0.00047	0.00035	0.00044	0.00051	0.00044	0.00030	0.00023	0.00028	0.00033	0.00033	0.00032	0.00020	0.00022	0.00032
241.07	12.938	-		0.00003	0.00001	0.00013	0.00004	0.00006	0.00004	0.00004	0.00001	0.00017	0.00003	0.00004	0.00003	0.00003	0.00012	0.00006	0.00014	0.00006	0.00003	0.00007	0.00004	0.00007
241.07	14.604	-		0.00002	0.00004	0.00007	0.00009	0.00007	0.00002	0.00004	0.00004	0.00012	0.00007	0.00007	0.00004	0.00002	0.00004	0.00002	0.00004	0.00002	0.00002	0.00001	0.00001	0.00004
241.07	11.315	-		0.00008	0.00015	0.00001	0.00003	0.00000	0.00002	0.00006	0.00006	0.00001	0.00003	0.00002	0.00002	0.00001	0.00001	0.00000	0.00002	0.00002	0.00004	0.00022	0.00000	0.00005
241.22	17.449	-		0.00004	0.00011	0.00007	0.00007	0.00006	0.00012	0.00007	0.00003	0.00010	0.00007	0.00008	0.00032	0.00011	0.00012	0.00009	0.00006	0.00006	0.00017	0.00013	0.00008	0.00009
242.18	12.643	-		0.00248	0.00049	0.00223	0.00239	0.00126	0.00239	0.00236	0.00300	0.00059	0.00281	0.00112	0.00040	0.00036	0.00158	0.00043	0.00071	0.00306	0.00055	0.00078	0.00282	0.00250
243.02	2.05	-		0.00000	0.00004	0.00010	0.00000	0.00000	0.00004	0.00000	0.00009	0.00020	0.00002	0.00008	0.00013	0.00000	0.00000	0.00000	0.00000	0.00000	0.00001	0.00023	0.00007	0.00014
243.14	16.75	+		0.00067	0.00047	0.00049	0.00051	0.00110	0.00095	0.00000	0.00072	0.00012	0.00050	0.00044	0.00104	0.00051	0.00050	0.00073	0.00050	0.00013	0.00134	0.00073	0.00077	0.00054
244.19	12.656	+		0.00129	0.00028	0.00065	0.00132	0.00067	0.00136	0.00168	0.00208	0.00019	0.00083	0.00084	0.00028	0.00011	0.00097	0.00028	0.00021	0.00088	0.00038	0.00042	0.00085	0.00078
244.99	13.858	-		0.00034	0.00056	0.00023	0.00022	0.00068	0.00052	0.00064	0.00038	0.00041	0.00025	0.00064	0.00051	0.00044	0.00043	0.00079	0.00074	0.00072	0.00103	0.00073	0.00038	0.00057
244.99	11.484	-		0.00009	0.00000	0.00002	0.00000	0.00008	0.00002	0.00002	0.00045	0.00015	0.00007	0.00022	0.00025	0.00008	0.00048	0.00024	0.00021	0.00078	0.00089	0.00000	0.00000	0.00000
244.99	15.451	-		0.00006	0.00017	0.00020	0.00008	0.00006	0.00010	0.00029	0.00045	0.00056	0.00036	0.00029	0.00019	0.00038	0.00009	0.00028	0.00053	0.00018	0.00044	0.00021	0.00066	0.00020
244.99	12.518	-		0.00002	0.00073	0.00047	0.00000	0.00000	0.00000	0.00000	0.00059	0.00062	0.00018	0.00054	0.00124	0.00014	0.00034	0.00005	0.00034	0.00006	0.00077	0.00028	0.00000	0.00000	0.00059
245.04	2.121	-		0.00009	0.00002	0.00010	0.00000	0.00000	0.00004	0.00010	0.00016	0.00008	0.00008	0.00004	0.00011	0.00025	0.00005	0.00011	0.00013	0.00004	0.00000	0.00010	0.00009	0.00005
248.08	13.509	-		0.00028	0.00030	0.00038	0.00092	0.00016	0.00050	0.00045	0.00067	0.00018	0.00086	0.00031	0.00011	0.00016	0.00040	0.00029	0.00030	0.00060	0.00020	0.00027	0.00026	0.00012
249.15	17.375	-		0.00016	0.00005	0.00017	0.00016	0.00015	0.00010	0.00017	0.00019	0.00006	0.00022	0.00013	0.00007	0.00012	0.00018	0.00003	0.00012	0.00022	0.00009	0.00008	0.00011	0.00007
250.17	12.802	+		0.00127	0.00061	0.00119	0.00123	0.00053	0.00122	0.00121	0.00146	0.00065	0.00146	0.00053	0.00000	0.00047	0.00096	0.00031	0.00019	0.00176	0.00066	0.00018	0.00163	0.00135
253.14	12.823	-		0.00005	0.00001	0.00001	0.00005	0.00003	0.00001	0.00001	0.00001	0.00001	0.00001	0.00004	0.00001	0.00001	0.00006	0.00002	0.00001	0.00002	0.00007	0.00002	0.00004	0.00001	0.00002
253.21	17.368	-		0.00000	0.00000	0.00002	0.00004	0.00002	0.00004	0.00006	0.00002	0.00001	0.00002	0.00001	0.00005	0.00001	0.00001	0.00001	0.00002	0.00002	0.00000	0.00000	0.00001	0.00001
255.12	17.86	-		0.00006	0.00003	0.00004	0.00000	0.00010	0.00005	0.00013	0.00005	0.00017	0.00004	0.00012	0.00012	0.00006	0.00006	0.00009	0.00005	0.00006	0.00003	0.00014	0.00000	0.00004
259.02	2.061	-		0.00075	0.00050	0.00036	0.00063	0.00049	0.00026	0.00066	0.00048	0.00049	0.00083	0.00048	0.00043	0.00060	0.00038	0.00030	0.00065	0.00059	0.00046	0.00083	0.00053	0.00032
261.11	12.364	+		0.00126	0.00171	0.00121	0.00152	0.00132	0.00116	0.00142	0.00160	0.00181	0.00158	0.00151	0.00159	0.00160	0.00139	0.00171	0.00176	0.00170	0.00181	0.00110	0.00137	0.00128
265.15	15.893	-		0.00003	0.00016	0.00008	0.00012	0.00009	0.00006	0.00005	0.00013	0.00017	0.00010	0.00015	0.00004	0.00004	0.00007	0.00013	0.00018	0.00008	0.00019	0.00011	0.00005	0.00006
265.15	15.538	-		0.00012	0.00008	0.00003	0.00007	0.00003	0.00011	0.00000	0.00003	0.00004	0.00003	0.00002	0.00002	0.00000	0.00010	0.00003	0.00003	0.00004	0.00000	0.00011	0.00001	0.00004
265.16	16.72	+		0.00114	0.00153	0.00133	0.00088	0.00070	0.00154	0.00187	0.00067	0.00121	0.00030	0.00167	0.00113	0.00156	0.00156	0.00178	0.00153	0.00182	0.00127	0.00172	0.00128	0.00156
267.20	14.996	-		0.00010	0.00002	0.00005	0.00016	0.00014	0.00012	0.00009	0.00014	0.00003	0.00003	0.00013	0.00013	0.00004	0.00011	0.00005	0.00003	0.00004	0.00000	0.00008	0.00005	0.00004
271.06	2.133	-		0.00006	0.00000	0.00000	0.00005	0.00000	0.00000	0.00000	0.00003	0.00000	0.00011	0.00000	0.00000	0.00002	0.00000	0.00000	0.00000	0.00001	0.00000	0.00002	0.00000	0.00000
271.08	12.542	-		0.00032	0.00037	0.00045	0.00043	0.00026	0.00004	0.00023	0.00013	0.00046	0.00033	0.00008	0.00023	0.00045	0.00033	0.00013	0.00025	0.00040	0.00005	0.00040	0.00015	0.00028
271.23	16.393	-		0.00009	0.00005	0.00012	0.00008	0.00015	0.00010	0.00007	0.00010	0.00007	0.00007	0.00009	0.00029	0.00005	0.00008	0.00002	0.00005	0.00007	0.00001	0.00011	0.00004	0.00007
273.07	9.482	+		0.00282	0.00344	0.00341	0.00458	0.00461	0.00412	0.00218	0.00221	0.00338	0.00212	0.00257	0.00322	0.00368	0.00326	0.00356	0.00289	0.00289	0.00313	0.00240	0.00282	0.00405
277.06	12.77	+		0.00058	0.00040	0.00044	0.00000	0.00000	0.00040	0.00074	0.00078	0.00021	0.00000	0.00083	0.00045	0.00099	0.00042	0.00037	0.00044	0.00000	0.00161	0.00050	0.00060	0.00045
277.15	15.124	-		0.00013	0.00014	0.00025	0.00012	0.00021	0.00011	0.00016	0.00013	0.00012	0.00012	0.00016	0.00010	0.00017	0.00012	0.00008	0.00012	0.00019	0.00008	0.00019	0.00017	0.00020
277.18	14.76	+		0.00415	0.00412	0.00305	0.00451	0.00421	0.00370	0.00397	0.00400	0.00372	0.00336	0.00364	0.00469	0.00458	0.00474	0.00468	0.00429	0.00438	0.00421	0.00472	0.00468	0.00371
277.18	13.655	+		0.01285	0.00164	0.01150	0.01200	0.00304	0.01150	0.02170	0.01390	0.00475	0.02166	0.00606	0.01350	0.00146	0.01216	0.00196	0.00404	0.01170	0.00043	0.00394	0.01070	0.00277
277.18	14.781	-		0.00050	0.00038	0.00034	0.00036	0.00049	0.00022	0.00029	0.00047	0.00018	0.00026	0.00053	0.00033	0.00035	0.00039	0.00030	0.00039	0.00021	0.00047	0.00037	0.00018	0.00035
279.09	10.75	-		0.00029	0.00016	0.00000	0.00092	0.00077	0.00011	0.00087	0.00065	0.00008	0.00025	0.00023	0.00004	0.00000	0.00000	0.00000	0.00063	0.00070	0.00010	0.00000	0.00004	0.00000
279.13	15.311	+		0.00120	0.00075	0.00045	0.00141	0.00077	0.00099	0.00063	0.00020	0.00035	0.00000	0.00023	0.00096	0.00030	0.00075	0.00076	0.00104	0.00115	0.00077	0.00086	0.00028	0.00000
279.16	15.345	+		0.00088	0.00063	0.00059	0.00096	0.00084	0.00077	0.00067	0.00079	0.00096	0.00065	0.00080	0.00077	0.00069	0.00114	0.00103	0.00096	0.00063	0.00072	0.00140	0.00088	0.00074
279.23	17.55	-		0.00002	0.00003	0.00002	0.00001	0.00002	0.00004	0.00006	0.00001	0.00001	0.00003	0.00001	0.00001	0.00000	0.00003	0.00000	0.00000	0.00000	0.00001	0.00004	0.00001	0.00002
280.98	15.993	-		0.00250	0.00390	0.00145	0.00000	0.00000	0.00143	0.00203	0.00138	0.00212	0.00134	0.00299	0.00000	0.00021	0.00358	0.00075	0.00115	0.00123	0.00108	0.00034	0.00205	0.00126
280.98	13.458	-		0.00336	0.00412	0.00306	0.00308	0.00322	0.00276	0.00407	0.00378	0.00337	0.00240	0.00357	0.00376	0.00293	0.00374	0.00340	0.00375	0.00325	0.00343	0.00359	0.00292	0.00314
281.01	8.021	+		0.00066	0.00008	0.00040	0.00059	0.00047	0.00080	0.00037	0.00030	0.00010	0.00000	0.00021	0.00000	0.00012	0.00000	0.00000	0.00054	0.00010	0.00022	0.00031	0.00012	0.00018
281.03	7.845	+		0.00025	0.00019	0.00036	0.00000	0.00000	0.00000	0.00033	0.00050	0.00035	0.00020	0.00000	0.00045	0.00020	0.00009	0.00039	0.00066	0.00018	0.00044	0.00019	0.00038	0.00016
281.05	2.437	+		0.00036	0.00052	0.00000	0.00102	0.00082	0.00027	0.00077	0.00103	0.00058	0.00104	0.00047	0.00042	0.00026	0.00102	0.00061	0.00000	0.00151	0.00030	0.00000	0.00046	0.00055
281.25	18.034	-		0.00052	0.00063	0.00050	0.00064	0.00057	0.00055	0.00060	0.00050	0.00057	0.00062	0.00053	0.00052	0.00047	0.00068	0.00049	0.00058	0.00058	0.00053	0.00061	0.00046	0.00056
284.27	17.497	-		0.00002	0.00003	0.00003	0.00001	0.00002	0.00001	0.00000	0.00001	0.00002	0.00004	0.00001	0.00003	0.00002	0.00003	0.00000	0.00002	0.00001	0.00001	0.00000	0.00000	0.00002
287.05	10.431	+		0.01643	0.01673	0.01540	0.01060	0.01220	0.00793	0.02240	0.01870	0.01440	0.01904	0.01860	0.01630	0.01650	0.01155	0.00850	0.02630	0.02110	0.01680	0.01000	0.00858	0.00722
287.05	8.35	+		0.00000	0.00000	0.00000	0.00000	0.00006	0.00017	0.00000	0.00000	0.00000	0.00000	0.00000	0.00000	0.00256	0.00109	0.00123	0.00000	0.00000	0.00000	0.00000	0.00000	0.00014
289.07	12.401	-		0.00002	0.00016	0.00010	0.00008	0.00035	0.00011	0.00000	0.00004	0.00004	0.00004	0.00009	0.00006	0.00000	0.00001	0.00002	0.00028	0.00002	0.00013	0.00003	0.00008	0.00019
289.07	8.913	+		0.01909	0.02251	0.02180	0.02290	0.02120	0.01910	0.01740	0.02120	0.02300	0.01670	0.02120	0.02300	0.02280	0.02082	0.02320	0.02080	0.02340	0.02490	0.01880	0.02070	0.02300
289.07	9.176	-		0.01577	0.01705	0.01900	0.01870	0.01900	0.01630	0.01950	0.01710	0.02150	0.01897	0.01691	0.01530	0.01080	0.01294	0.00957	0.02080	0.01590	0.01650	0.02660	0.01820	0.02190
289.07	7.642	+		0.00045	0.00064	0.00152	0.00032	0.00063	0.00025	0.00056	0.00366	0.00040	0.00052	0.00081	0.00090	0.00011	0.00024	0.00058	0.00134	0.00078	0.00026	0.00057	0.00117	0.00252
289.07	10.498	-		0.00000	0.00282	0.00000	0.00000	0.00002	0.00003	0.00582	0.00562	0.00545	0.00000	0.00008	0.00005	0.00000	0.00000	0.00000	0.00000	0.00000	0.00004	0.00003	0.00000	0.00000
289.09	8.574	+		0.00389	0.00000	0.00000	0.00229	0.00000	0.00000	0.01960	0.00000	0.00263	0.00152	0.02381	0.00226	0.00016	0.00000	0.00000	0.00583	0.00213	0.00000	0.00152	0.00000	0.00275
289.21	9.177	-		0.00054	0.00063	0.00104	0.00103	0.00109	0.00096	0.00111	0.00056	0.00122	0.00104	0.00087	0.00082	0.00056	0.00070	0.00031	0.00116	0.00085	0.00088	0.00104	0.00097	0.00083
289.40	9.177	-		0.00054	0.00036	0.00068	0.00062	0.00065	0.00055	0.00069	0.00037	0.00075	0.00041	0.00057	0.00000	0.00011	0.00027	0.00000	0.00073	0.00017	0.00020	0.00092	0.00063	0.00076
289.93	2.793	-		0.00007	0.00001	0.00003	0.00016	0.00014	0.00016	0.00015	0.00017	0.00001	0.00005	0.00018	0.00015	0.00008	0.00009	0.00012	0.00015	0.00007	0.00000	0.00002	0.00001	0.00011
291.08	9.161	+		0.01825	0.02057	0.02290	0.02310	0.02480	0.02180	0.02210	0.02110	0.02490	0.02177	0.01931	0.02100	0.01470	0.01717	0.01330	0.02360	0.01950	0.02200	0.02810	0.02310	0.02620
291.11	8.032	-		0.00004	0.00006	0.00004	0.00002	0.00005	0.00000	0.00000	0.00000	0.00000	0.00002	0.00002	0.00001	0.00000	0.00000	0.00000	0.00006	0.00006	0.00004	0.00005	0.00008	0.00010
291.13	16.138	-		0.00044	0.00033	0.00061	0.00015	0.00015	0.00024	0.00000	0.00000	0.00012	0.00057	0.00036	0.00052	0.00052	0.00018	0.00007	0.00000	0.00000	0.00000	0.00045	0.00055	0.00042
292.99	1.97	+		0.00128	0.00087	0.00093	0.00222	0.00247	0.00201	0.00183	0.00209	0.00177	0.00096	0.00136	0.00102	0.00198	0.00237	0.00198	0.00260	0.00178	0.00186	0.00219	0.00165	0.00174
293.00	13.644	-		0.00000	0.00004	0.00057	0.00084	0.00016	0.00040	0.00119	0.00048	0.00000	0.00109	0.00020	0.00108	0.00002	0.00048	0.00003	0.00006	0.00043	0.00000	0.00026	0.00050	0.00013
293.03	13.642	-		0.00029	0.00000	0.00067	0.00065	0.00000	0.00062	0.00103	0.00017	0.00013	0.00061	0.00041	0.00047	0.00004	0.00073	0.00002	0.00000	0.00038	0.00000	0.00010	0.00057	0.00006
293.17	13.644	-		0.01638	0.00206	0.01460	0.01560	0.00400	0.01450	0.02700	0.01680	0.00584	0.02567	0.00785	0.01820	0.00174	0.01563	0.00241	0.00513	0.01520	0.00090	0.00502	0.01260	0.00341
293.32	13.644	-		0.00074	0.00004	0.00065	0.00068	0.00015	0.00065	0.00131	0.00078	0.00022	0.00122	0.00032	0.00081	0.00004	0.00069	0.00008	0.00019	0.00067	0.00000	0.00018	0.00056	0.00013
293.50	13.643	-		0.00090	0.00002	0.00081	0.00087	0.00012	0.00082	0.00162	0.00096	0.00029	0.00156	0.00040	0.00105	0.00000	0.00087	0.00010	0.00025	0.00085	0.00000	0.00023	0.00071	0.00010
295.00	10.928	-		0.00010	0.00097	0.00012	0.00035	0.00021	0.00000	0.00061	0.00025	0.00013	0.00008	0.00033	0.00058	0.00008	0.00108	0.00006	0.00009	0.00010	0.00019	0.00013	0.00028	0.00010
295.08	10.23	-		0.00018	0.00016	0.00000	0.00086	0.00049	0.00017	0.00037	0.00035	0.00000	0.00003	0.00012	0.00000	0.00000	0.00000	0.00000	0.00020	0.00025	0.00000	0.00000	0.00004	0.00000
297.08	12.852	-		0.00007	0.00005	0.00003	0.00007	0.00003	0.00003	0.00003	0.00020	0.00009	0.00018	0.00012	0.00013	0.00002	0.00016	0.00011	0.00011	0.00012	0.00030	0.00012	0.00000	0.00003
297.15	13.752	-		0.00006	0.00001	0.00005	0.00000	0.00006	0.00001	0.00002	0.00001	0.00002	0.00010	0.00001	0.00001	0.00004	0.00002	0.00001	0.00001	0.00008	0.00003	0.00007	0.00013	0.00005
297.15	14.776	-		0.00008	0.00012	0.00005	0.00011	0.00019	0.00011	0.00021	0.00007	0.00012	0.00011	0.00007	0.00004	0.00008	0.00004	0.00001	0.00005	0.00010	0.00009	0.00020	0.00008	0.00014
297.16	16.106	-		0.00022	0.00019	0.00017	0.00004	0.00025	0.00091	0.00009	0.00055	0.00020	0.00003	0.00015	0.00031	0.00009	0.00011	0.00009	0.00026	0.00044	0.00007	0.00069	0.00005	0.00009
297.24	16.4	-		0.00001	0.00001	0.00005	0.00003	0.00001	0.00001	0.00000	0.00000	0.00004	0.00003	0.00000	0.00002	0.00001	0.00001	0.00000	0.00008	0.00005	0.00000	0.00000	0.00004	0.00006
298.09	8.366	+		0.00135	0.00082	0.00106	0.00152	0.00126	0.00075	0.00140	0.00130	0.00102	0.00065	0.00133	0.00139	0.00093	0.00091	0.00107	0.00231	0.00197	0.00102	0.00146	0.00104	0.00125
299.00	12.006	+		0.00077	0.00072	0.00060	0.00069	0.00057	0.00054	0.00065	0.00073	0.00067	0.00073	0.00071	0.00067	0.00072	0.00065	0.00076	0.00065	0.00095	0.00078	0.00069	0.00068	0.00060
299.15	14.767	+		0.00006	0.00006	0.00000	0.00016	0.00019	0.00000	0.00011	0.00006	0.00000	0.00007	0.00006	0.00018	0.00010	0.00019	0.00009	0.00009	0.00009	0.00008	0.00018	0.00011	0.00004
299.17	14.765	+		0.00007	0.00010	0.00006	0.00012	0.00000	0.00013	0.00000	0.00000	0.00010	0.00005	0.00000	0.00000	0.00004	0.00000	0.00000	0.00010	0.00000	0.00000	0.00002	0.00007	0.00012
299.20	16.827	-		0.00020	0.00027	0.00016	0.00007	0.00006	0.00006	0.00028	0.00012	0.00006	0.00011	0.00019	0.00006	0.00004	0.00010	0.00000	0.00009	0.00008	0.00003	0.00000	0.00007	0.00017
299.25	16.769	-		0.00004	0.00002	0.00001	0.00002	0.00001	0.00003	0.00002	0.00002	0.00001	0.00001	0.00001	0.00015	0.00001	0.00001	0.00001	0.00001	0.00002	0.00001	0.00004	0.00001	0.00002
301.00	10.56	-		0.00008	0.00014	0.00013	0.00009	0.00013	0.00001	0.00024	0.00010	0.00000	0.00003	0.00021	0.00015	0.00042	0.00012	0.00012	0.00025	0.00027	0.00018	0.00003	0.00010	0.00008
301.03	10.959	-		0.00013	0.00022	0.00033	0.00000	0.00000	0.00000	0.00005	0.00006	0.00018	0.00008	0.00017	0.00024	0.00021	0.00006	0.00017	0.00006	0.00014	0.00020	0.00000	0.00000	0.00000
301.14	15.348	+		0.00197	0.00175	0.00135	0.00211	0.00188	0.00187	0.00135	0.00156	0.00202	0.00139	0.00153	0.00167	0.00146	0.00246	0.00206	0.00198	0.00172	0.00162	0.00292	0.00213	0.00186
301.22	17.363	-		0.00012	0.00031	0.00021	0.00004	0.00011	0.00012	0.00025	0.00016	0.00003	0.00005	0.00022	0.00011	0.00004	0.00004	0.00001	0.00003	0.00008	0.00003	0.00000	0.00000	0.00010
303.05	10.143	+		0.01680	0.01634	0.02530	0.00684	0.00858	0.00981	0.01650	0.01140	0.02090	0.02086	0.01255	0.01780	0.01960	0.02113	0.02060	0.01440	0.01110	0.01750	0.00584	0.00525	0.00543
303.05	7.85	-		0.00057	0.00069	0.00055	0.00085	0.00060	0.00043	0.00047	0.00056	0.00084	0.00070	0.00047	0.00053	0.00051	0.00065	0.00044	0.00063	0.00083	0.00044	0.00073	0.00066	0.00084
303.06	10.533	+		0.10609	0.04191	0.05430	0.03130	0.00286	0.02780	0.00854	0.02980	0.00827	0.05213	0.00146	0.00307	0.00006	0.04873	0.00424	0.00000	0.03010	0.00784	0.03150	0.01330	0.01390
303.10	11.696	+		0.00076	0.00130	0.00086	0.00034	0.00120	0.00033	0.00082	0.00138	0.00091	0.00070	0.00058	0.00105	0.00140	0.00052	0.00135	0.00115	0.00041	0.00065	0.00068	0.00065	0.00076

(continued)

			(Name)	AT1	AT2	AT3	BF1	BF2	BF3	KM1	KM2	KM3	SM1	SM2	SM3	SR1	SR2	SR3	YB1	YB2	YB3	YM1	YM2	YM3
			(ORAC)	35,647	38,661	51,482	42,552	65,085	55,612	44,400	43,107	48,544	25,445	40,191	46,160	37,930	42,436	38,953	34,246	36,545	41,729	37,746	43,496	47,566
m/z	R.T.	Polar	olyphenol)	1.93	2.11	2.54	2.04	2.56	2.54	2.00	2.11	2.41	1.96	2.13	2.33	2.44	2.33	2.17	2.12	2.13	2.34	2.21	2.28	2.65
303.21	16.293	+		0.00014	0.00099	0.00125	0.00067	0.00094	0.00101	0.00000	0.00149	0.00059	0.00167	0.00149	0.00118	0.00068	0.00106	0.00050	0.00069	0.00109	0.00116	0.00101	0.00183	0.00098
303.23	16.157	+		0.00040	0.00104	0.00089	0.00119	0.00135	0.00052	0.00153	0.00011	0.00098	0.00270	0.00068	0.00199	0.00097	0.00171	0.00161	0.00017	0.00183	0.00062	0.00000	0.00079	0.00069
303.60	11.705	+		0.00005	0.00043	0.00004	0.00013	0.00059	0.00006	0.00008	0.00029	0.00007	0.00018	0.00011	0.00009	0.00032	0.00000	0.00021	0.00007	0.00000	0.00015	0.00019	0.00003	0.00004
304.06	7.816	-		0.00074	0.00079	0.00109	0.00081	0.00075	0.00069	0.00116	0.00069	0.00043	0.00078	0.00067	0.00097	0.00050	0.00084	0.00068	0.00048	0.00073	0.00086	0.00147	0.00061	0.00091
304.88	8.263	-		0.00029	0.00002	0.00071	0.00032	0.00000	0.00018	0.00019	0.00019	0.00084	0.00036	0.00069	0.00065	0.00000	0.00010	0.00000	0.00040	0.00000	0.00002	0.00002	0.00027	0.00079
304.91	8.354	-		0.00153	0.00109	0.00182	0.00083	0.00287	0.00077	0.00051	0.00041	0.00138	0.00103	0.00047	0.00048	0.00057	0.00051	0.00063	0.00118	0.00189	0.00058	0.00256	0.00155	0.00000
304.95	7.828	-		0.00023	0.00178	0.00080	0.00047	0.00011	0.00009	0.00127	0.00015	0.00057	0.00430	0.00014	0.00112	0.00239	0.00007	0.00003	0.00023	0.00020	0.00116	0.00007	0.00455	0.00084
305.06	8.485	-		0.04370	0.05356	0.06660	0.04580	0.05230	0.04240	0.05740	0.05240	0.07700	0.05677	0.05876	0.05900	0.02510	0.02700	0.01980	0.05470	0.04300	0.05830	0.07100	0.05880	0.07140
305.07	11.404	-		0.00006	0.00008	0.00015	0.00007	0.00005	0.00025	0.00024	0.00017	0.00023	0.00007	0.00024	0.00013	0.00007	0.00004	0.00005	0.00017	0.00013	0.00022	0.00005	0.00006	0.00000
305.07	10.455	-		0.00008	0.00006	0.00034	0.00005	0.00000	0.00009	0.00009	0.00058	0.00023	0.00004	0.00002	0.00033	0.00000	0.00000	0.00008	0.00000	0.00011	0.00016	0.00010	0.00000	0.00000
305.08	12.266	-		0.00044	0.00072	0.00117	0.00002	0.00002	0.00010	0.00008	0.00007	0.00059	0.00006	0.00042	0.00030	0.00016	0.00027	0.00035	0.00008	0.00005	0.00018	0.00048	0.00052	0.00067
305.19	16.358	-		0.00004	0.00007	0.00005	0.00009	0.00006	0.00005	0.00011	0.00006	0.00009	0.00005	0.00006	0.00005	0.00003	0.00005	0.00003	0.00006	0.00007	0.00004	0.00010	0.00003	0.00008
305.22	8.481	-		0.00250	0.00313	0.00384	0.00255	0.00303	0.00243	0.00324	0.00299	0.00444	0.00329	0.00331	0.00346	0.00135	0.00146	0.00099	0.00308	0.00242	0.00327	0.00413	0.00333	0.00418
305.31	8.478	-		0.00143	0.00181	0.00225	0.00146	0.00175	0.00140	0.00191	0.00174	0.00259	0.00184	0.00198	0.00196	0.00075	0.00081	0.00017	0.00182	0.00141	0.00191	0.00238	0.00193	0.00246
305.40	8.478	-		0.00154	0.00188	0.00241	0.00160	0.00185	0.00148	0.00203	0.00184	0.00277	0.00206	0.00209	0.00219	0.00083	0.00091	0.00062	0.00194	0.00151	0.00203	0.00266	0.00210	0.00257
306.07	2.244	-		0.00001	0.00000	0.00005	0.00023	0.00000	0.00000	0.00011	0.00000	0.00006	0.00004	0.00001	0.00002	0.00013	0.00000	0.00002	0.00014	0.00000	0.00000	0.00018	0.00006	0.00000
307.08	8.494	+		0.04757	0.05722	0.06270	0.04760	0.05010	0.04610	0.05530	0.05680	0.07340	0.05734	0.05941	0.06140	0.03040	0.03241	0.02730	0.05570	0.04930	0.06060	0.06690	0.06100	0.06850
307.08	2.367	+		0.00719	0.00519	0.00450	0.00594	0.00290	0.00276	0.00865	0.00875	0.00489	0.00635	0.00957	0.00585	0.00411	0.00247	0.00314	0.01040	0.00545	0.00432	0.00681	0.00521	0.00341
307.14	10.499	-		0.00073	0.00135	0.00041	0.00022	0.00023	0.00015	0.00028	0.00038	0.00052	0.00080	0.00116	0.00042	0.00050	0.00041	0.00011	0.00049	0.00046	0.00031	0.00052	0.00049	0.00023
307.59	2.371	+		0.00169	0.00090	0.00093	0.00161	0.00052	0.00066	0.00096	0.00136	0.00090	0.00114	0.00182	0.00063	0.00082	0.00068	0.00036	0.00205	0.00133	0.00055	0.00181	0.00125	0.00077
307.85	3.475	-		0.00000	0.00003	0.00001	0.00000	0.00000	0.00004	0.00002	0.00001	0.00001	0.00002	0.00008	0.00004	0.00003	0.00012	0.00000	0.00000	0.00001	0.00003	0.00009	0.00000	0.00001
307.85	5.33	-		0.00000	0.00009	0.00003	0.00002	0.00002	0.00002	0.00006	0.00005	0.00009	0.00008	0.00006	0.00004	0.00007	0.00007	0.00003	0.00003	0.00003	0.00001	0.00004	0.00005	0.00005
307.86	3.864	-		0.00006	0.00001	0.00002	0.00004	0.00005	0.00004	0.00002	0.00006	0.00006	0.00004	0.00011	0.00010	0.00009	0.00007	0.00005	0.00007	0.00005	0.00004	0.00005	0.00004	0.00002
307.86	6.73	-		0.00011	0.00005	0.00006	0.00010	0.00004	0.00006	0.00011	0.00009	0.00007	0.00008	0.00004	0.00008	0.00012	0.00013	0.00003	0.00004	0.00009	0.00005	0.00009	0.00006	0.00010
309.09	11.188	+		0.00000	0.00000	0.00000	0.00195	0.00258	0.00291	0.00000	0.00000	0.00069	0.00000	0.00039	0.00038	0.00129	0.00216	0.00258	0.00000	0.00000	0.00034	0.00263	0.00247	0.00265
309.17	13.655	-		0.00081	0.00013	0.00078	0.00081	0.00029	0.00102	0.00076	0.00111	0.00055	0.00330	0.00041	0.00089	0.00019	0.00096	0.00014	0.00047	0.00085	0.00003	0.00048	0.00076	0.00024
309.20	15.994	+		0.00089	0.00095	0.00093	0.00089	0.00083	0.00063	0.00086	0.00101	0.00061	0.00054	0.00060	0.00069	0.00077	0.00103	0.00100	0.00100	0.00068	0.00075	0.00059	0.00099	0.00073
311.17	12.27	-		0.00003	0.00007	0.00003	0.00003	0.00039	0.00005	0.00004	0.00000	0.00006	0.00001	0.00008	0.00010	0.00005	0.00006	0.00001	0.00014	0.00010	0.00003	0.00015	0.00007	0.00012
312.17	16.696	-		0.00006	0.00008	0.00003	0.00012	0.00010	0.00013	0.00002	0.00004	0.00005	0.00006	0.00006	0.00003	0.00004	0.00001	0.00004	0.00008	0.00003	0.00007	0.00001	0.00001	0.00004
312.17	15.394	-		0.00002	0.00007	0.00004	0.00015	0.00006	0.00002	0.00012	0.00009	0.00010	0.00011	0.00007	0.00013	0.00007	0.00006	0.00004	0.00006	0.00007	0.00004	0.00012	0.00007	0.00006
313.09	16.63	-		0.00003	0.00007	0.00007	0.00032	0.00005	0.00008	0.00018	0.00017	0.00001	0.00000	0.00017	0.00000	0.00011	0.00005	0.00001	0.00006	0.00014	0.00010	0.00001	0.00006	0.00004
313.10	15.261	-		0.00006	0.00002	0.00034	0.00020	0.00003	0.00019	0.00015	0.00016	0.00010	0.00000	0.00004	0.00001	0.00030	0.00002	0.00008	0.00013	0.00008	0.00032	0.00019	0.00008	0.00006
313.16	15.306	-		0.00004	0.00003	0.00005	0.00013	0.00000	0.00008	0.00011	0.00006	0.00003	0.00003	0.00002	0.00009	0.00005	0.00003	0.00002	0.00004	0.00006	0.00005	0.00007	0.00004	0.00002
313.24	15.117	-		0.00080	0.00101	0.00086	0.00102	0.00090	0.00087	0.00092	0.00104	0.00098	0.00092	0.00096	0.00092	0.00082	0.00097	0.00093	0.00099	0.00098	0.00099	0.00107	0.00090	0.00087
315.02	8.248	-		0.00000	0.00007	0.00000	0.00000	0.00000	0.00000	0.00000	0.00001	0.00001	0.00003	0.00007	0.00004	0.00032	0.00011	0.00010	0.00013	0.00008	0.00000	0.00002	0.00000	0.00000
315.07	8.006	-		0.00082	0.00023	0.00030	0.00005	0.00006	0.00002	0.00000	0.00036	0.00009	0.00008	0.00002	0.00016	0.00004	0.00018	0.00022	0.00012	0.00006	0.00003	0.00008	0.00045	0.00014
315.09	15.429	-		0.00000	0.00000	0.00001	0.00000	0.00000	0.00008	0.00000	0.00000	0.00000	0.00000	0.00000	0.00000	0.00000	0.00000	0.00000	0.00000	0.00000	0.00000	0.00007	0.00003	0.00000
315.09	7.737	+		0.00014	0.00053	0.00075	0.00000	0.00048	0.00007	0.00000	0.00050	0.00043	0.00000	0.00021	0.00036	0.00020	0.00022	0.00000	0.00054	0.00037	0.00020	0.00012	0.00012	0.00029
315.10	11.635	+		0.00000	0.00008	0.00000	0.00000	0.00000	0.00000	0.00000	0.00013	0.00000	0.00000	0.00000	0.00000	0.00012	0.00000	0.00000	0.00000	0.00011	0.00007	0.00000	0.00017	0.00027
315.53	8.246	-		0.00032	0.00036	0.00032	0.00041	0.00028	0.00009	0.00038	0.00023	0.00020	0.00030	0.00047	0.00039	0.00130	0.00056	0.00074	0.00061	0.00055	0.00029	0.00023	0.00022	0.00022
316.03	8.248	-		0.00139	0.00179	0.00127	0.00179	0.00137	0.00043	0.00164	0.00097	0.00089	0.00127	0.00208	0.00185	0.00575	0.00273	0.00333	0.00274	0.00239	0.00124	0.00102	0.00098	0.00098
316.53	8.251	-		0.00023	0.00032	0.00025	0.00031	0.00025	0.00008	0.00031	0.00019	0.00014	0.00024	0.00042	0.00032	0.00105	0.00044	0.00060	0.00047	0.00041	0.00024	0.00017	0.00016	0.00019
317.05	2.087	-		0.00049	0.00019	0.00024	0.00026	0.00013	0.00033	0.00026	0.00023	0.00018	0.00043	0.00040	0.00029	0.00000	0.00002	0.00000	0.00038	0.00027	0.00022	0.00048	0.00024	0.00006
317.10	10.607	+		0.00088	0.00089	0.00110	0.00000	0.00102	0.00038	0.00000	0.00046	0.00132	0.00040	0.00117	0.00081	0.00116	0.00081	0.00088	0.00105	0.00000	0.00028	0.00022	0.00051	0.00080
317.18	13.691	+		0.00034	0.00000	0.00076	0.00089	0.00000	0.00025	0.00204	0.00047	0.00032	0.00043	0.00000	0.00000	0.00000	0.00000	0.00000	0.00058	0.00053	0.00000	0.00000	0.00119	0.00037
319.02	2.215	-		0.00015	0.00001	0.00000	0.00000	0.00000	0.00000	0.00021	0.00002	0.00000	0.00017	0.00007	0.00000	0.00001	0.00003	0.00000	0.00019	0.00005	0.00000	0.00010	0.00008	0.00000
319.04	9.903	+		0.00465	0.00460	0.00813	0.00255	0.00385	0.00465	0.00443	0.00358	0.00800	0.00712	0.00480	0.00916	0.01780	0.01814	0.01900	0.00504	0.00465	0.00919	0.00335	0.00293	0.00415
321.09	10.924	-		0.00008	0.00001	0.00001	0.00000	0.00000	0.00000	0.00012	0.00008	0.00002	0.00013	0.00018	0.00031	0.00000	0.00000	0.00000	0.00013	0.00012	0.00006	0.00000	0.00000	0.00000
323.22	17.855	-		0.00050	0.00058	0.00028	0.00054	0.00038	0.00052	0.00048	0.00028	0.00025	0.00041	0.00052	0.00024	0.00047	0.00054	0.00047	0.00051	0.00051	0.00037	0.00058	0.00042	0.00042
325.05	9.227	-		0.00072	0.00067	0.00073	0.00076	0.00000	0.00056	0.00086	0.00072	0.00083	0.00084	0.00070	0.00000	0.00030	0.00059	0.00035	0.00091	0.00000	0.00000	0.00115	0.00048	0.00074
325.07	12.006	+		0.00064	0.00030	0.00023	0.00049	0.00017	0.00039	0.00023	0.00042	0.00030	0.00024	0.00046	0.00017	0.00032	0.00036	0.00039	0.00027	0.00072	0.00046	0.00042	0.00027	0.00000
325.18	15.688	-		0.00022	0.00017	0.00031	0.00014	0.00008	0.00003	0.00024	0.00020	0.00016	0.00013	0.00006	0.00006	0.00014	0.00028	0.00015	0.00038	0.00032	0.00015	0.00017	0.00063	0.00010
326.19	16.515	-		0.00000	0.00002	0.00004	0.00001	0.00000	0.00009	0.00004	0.00004	0.00001	0.00001	0.00005	0.00005	0.00001	0.00002	0.00004	0.00010	0.00001	0.00006	0.00000	0.00006	0.00001
329.29	17.613	-		0.00009	0.00018	0.00026	0.00046	0.00012	0.00044	0.00029	0.00076	0.00020	0.00020	0.00011	0.00042	0.00046	0.00028	0.00077	0.00015	0.00045	0.00263	0.00064	0.00015	0.00001
330.98	15.19	-		0.00049	0.00027	0.00011	0.00000	0.00005	0.00005	0.00003	0.00020	0.00004	0.00015	0.00000	0.00002	0.00004	0.00013	0.00004	0.00004	0.00020	0.00002	0.00021	0.00043	0.00010
330.98	13.901	-		0.00005	0.00012	0.00010	0.00008	0.00010	0.00006	0.00009	0.00003	0.00005	0.00012	0.00006	0.00012	0.00015	0.00004	0.00005	0.00008	0.00013	0.00006	0.00007	0.00014	0.00007
331.06	3.556	-		0.00060	0.00079	0.00061	0.00056	0.00033	0.00009	0.00064	0.00022	0.00038	0.00055	0.00057	0.00054	0.00135	0.00070	0.00072	0.00075	0.00069	0.00028	0.00052	0.00036	0.00014
331.07	8.103	-		0.00018	0.00025	0.00026	0.00000	0.00006	0.00009	0.00000	0.00003	0.00008	0.00005	0.00002	0.00002	0.00002	0.00002	0.00001	0.00000	0.00006	0.00000	0.00000	0.00006	0.00010
331.25	13.989	-		0.00069	0.00060	0.00073	0.00081	0.00067	0.00059	0.00072	0.00077	0.00081	0.00080	0.00075	0.00081	0.00080	0.00071	0.00081	0.00079	0.00085	0.00095	0.00076	0.00080	0.00074
333.06	2.044	-		0.00043	0.00017	0.00039	0.00009	0.00016	0.00018	0.00032	0.00051	0.00024	0.00022	0.00016	0.00029	0.00099	0.00032	0.00050	0.00046	0.00029	0.00023	0.00006	0.00024	0.00022
335.14	2.332	-		0.00006	0.00006	0.00004	0.00016	0.00002	0.00007	0.00006	0.00025	0.00000	0.00044	0.00007	0.00004	0.00001	0.00008	0.00001	0.00005	0.00002	0.00000	0.00035	0.00008	0.00001
337.08	2.206	-		0.00015	0.00005	0.00007	0.00013	0.00005	0.00002	0.00016	0.00007	0.00001	0.00017	0.00009	0.00005	0.00003	0.00007	0.00002	0.00018	0.00008	0.00005	0.00017	0.00009	0.00004
337.09	9.563	-		0.00773	0.00222	0.00112	0.01220	0.01410	0.00512	0.01260	0.00871	0.00573	0.01594	0.01074	0.01050	0.00918	0.00654	0.00287	0.02220	0.01600	0.01070	0.01180	0.00672	0.00668
337.16	2.346	+		0.00194	0.00201	0.00184	0.00287	0.00049	0.00158	0.00183	0.00522	0.00061	0.00769	0.00254	0.00123	0.00106	0.00194	0.00092	0.00183	0.00116	0.00056	0.00576	0.00218	0.00103
339.05	12.358	+		0.00000	0.00012	0.00000	0.00000	0.00009	0.00018	0.00018	0.00047	0.00000	0.00000	0.00012	0.00026	0.00000	0.00000	0.00015	0.00000	0.00000	0.00013	0.00000	0.00022	0.00043
341.11	1.837	-		0.00713	0.00454	0.00603	0.00442	0.00193	0.00410	0.00598	0.00420	0.00377	0.00817	0.00492	0.00356	0.00264	0.00338	0.00384	0.00438	0.00437	0.00264	0.00727	0.00549	0.00332
342.06	6.052	-		0.00002	0.00002	0.00001	0.00021	0.00024	0.00008	0.00008	0.00000	0.00002	0.00005	0.00001	0.00001	0.00045	0.00037	0.00046	0.00004	0.00004	0.00001	0.00002	0.00000	0.00000
343.07	7.139	-		0.00070	0.00075	0.00135	0.00141	0.00187	0.00055	0.00153	0.00177	0.00013	0.00047	0.00078	0.00230	0.00040	0.00096	0.00116	0.00064	0.00070	0.00365	0.00062	0.00029	0.00025
345.08	6.022	+		0.00124	0.00096	0.00094	0.00336	0.00318	0.00173	0.00179	0.00089	0.00091	0.00153	0.00095	0.00071	0.00573	0.00521	0.00522	0.00235	0.00182	0.00051	0.00071	0.00082	0.00099
349.06	11.06	-		0.00008	0.00015	0.00011	0.00000	0.00000	0.00000	0.00003	0.00008	0.00018	0.00000	0.00027	0.00000	0.00000	0.00000	0.00000	0.00001	0.00000	0.00026	0.00143	0.00168	0.00166
349.15	10.998	-		0.00000	0.00003	0.00000	0.00000	0.00000	0.00000	0.00000	0.00000	0.00000	0.00002	0.00000	0.00003	0.00000	0.00000	0.00000	0.00000	0.00000	0.00000	0.00006	0.00016	0.00013
353.08	8.816	-		0.00000	0.00000	0.00000	0.00308	0.00357	0.00171	0.00000	0.00000	0.00000	0.00073	0.00050	0.00000	0.00682	0.00531	0.00000	0.00086	0.00000	0.00000	0.00434	0.00372	0.00048
357.12	10.14	-		0.00007	0.00000	0.00000	0.00001	0.00000	0.00000	0.00002	0.00000	0.00002	0.00001	0.00013	0.00000	0.00023	0.00004	0.00000	0.00009	0.00000	0.00000	0.00051	0.00046	0.00046
359.33	17.92	+		0.00105	0.00107	0.00052	0.00000	0.00079	0.00423	0.00089	0.00160	0.00078	0.00000	0.00057	0.00109	0.00006	0.00010	0.00074	0.00040	0.00101	0.00019	0.00000	0.00048	0.00069
365.12	8.71	+		0.00044	0.00000	0.00000	0.00165	0.00123	0.00029	0.00096	0.00031	0.00000	0.00065	0.00010	0.00000	0.00046	0.00034	0.00011	0.00105	0.00040	0.00000	0.00132	0.00064	0.00016
365.13	15.051	+		0.00000	0.00049	0.00000	0.00000	0.00025	0.00024	0.00000	0.00009	0.00016	0.00000	0.00009	0.00000	0.00000	0.00019	0.00000	0.00000	0.00000	0.00000	0.00000	0.00020	0.00037
367.29	17.959	+		0.00137	0.00059	0.00044	0.00000	0.00040	0.00145	0.00089	0.00066	0.00129	0.00254	0.00104	0.00022	0.00023	0.00156	0.00006	0.00122	0.00029	0.00022	0.00000	0.00227	0.00131
368.06	8.484	-		0.00126	0.00147	0.00101	0.00138	0.00148	0.00121	0.00161	0.00154	0.00180	0.00149	0.00161	0.00155	0.00058	0.00069	0.00053	0.00155	0.00135	0.00108	0.00177	0.00146	0.00106
371.10	4.254	+		0.00038	0.00120	0.00015	0.00048	0.00049	0.00030	0.00147	0.00127	0.00191	0.00000	0.00046	0.00000	0.00031	0.00127	0.00195	0.00222	0.00140	0.00070	0.00089	0.00077	0.00051
371.10	2.303	+		0.00101	0.00113	0.00099	0.00254	0.00071	0.00109	0.00092	0.00169	0.00174	0.00227	0.00092	0.00047	0.00097	0.00189	0.00185	0.00240	0.00137	0.00243	0.00169	0.00145	0.00201
371.10	6.359	+		0.00046	0.00056	0.00000	0.00000	0.00034	0.00006	0.00029	0.00123	0.00050	0.00016	0.00089	0.00045	0.00019	0.00102	0.00071	0.00015	0.00167	0.00035	0.00000	0.00018	0.00074
371.10	12.123	+		0.00052	0.00071	0.00049	0.00000	0.00000	0.00053	0.00031	0.00134	0.00109	0.00024	0.00148	0.00022	0.00063	0.00135	0.00175	0.00033	0.00115	0.00039	0.00025	0.00058	0.00091
371.10	8.233	+		0.00044	0.00089	0.00287	0.00000	0.00019	0.00000	0.00277	0.00125	0.00046	0.00765	0.00202	0.00071	0.00043	0.00039	0.00118	0.00251	0.00389	0.00161	0.00060	0.00511	0.00138
372.11	15.096	+		0.00000	0.00012	0.00042	0.00028	0.00018	0.00000	0.00000	0.00000	0.00018	0.00051	0.00000	0.00000	0.00026	0.00023	0.00000	0.00019	0.00087	0.00000	0.00012	0.00018	0.00012
377.08	2.103	-		0.00040	0.00026	0.00032	0.00061	0.00019	0.00035	0.00034	0.00031	0.00036	0.00068	0.00037	0.00027	0.00020	0.00021	0.00030	0.00022	0.00035	0.00019	0.00070	0.00031	0.00033
377.16	10.024	-		0.00017	0.00006	0.00004	0.00035	0.00042	0.00027	0.00044	0.00047	0.00031	0.00023	0.00032	0.00000	0.00000	0.00000	0.00000	0.00022	0.00010	0.00000	0.00054	0.00054	0.00050
377.18	10.679	-		0.00014	0.00024	0.00027	0.00031	0.00061	0.00013	0.00019	0.00045	0.00018	0.00007	0.00007	0.00000	0.00042	0.00084	0.00039	0.00008	0.00015	0.00008	0.00064	0.00048	0.00050
387.11	1.833	-		0.00877	0.00555	0.00733	0.00561	0.00269	0.00516	0.00697	0.00503	0.00475	0.00950	0.00609	0.00465	0.00369	0.00447	0.00485	0.00544	0.00545	0.00337	0.00882	0.00681	0.00434
387.13	8.696	-		0.00016	0.00000	0.00000	0.00105	0.00083	0.00027	0.00037	0.00006	0.00000	0.00029	0.00000	0.00000	0.00040	0.00040	0.00011	0.00055	0.00006	0.00000	0.00080	0.00000	0.00000
387.15	13.099	+		0.00190	0.00253	0.00054	0.00091	0.00269	0.00000	0.00047	0.00110	0.00096	0.00000	0.00169	0.00142	0.00000	0.00252	0.00080	0.00164	0.00000	0.00218	0.00237	0.00069	0.00194
387.18	12.77	-		0.00017	0.00032	0.00018	0.00019	0.00017	0.00016	0.00033	0.00034	0.00025	0.00009	0.00036	0.00053	0.00018	0.00021	0.00077	0.00024	0.00018	0.00054	0.00030	0.00017	0.00021
387.18	13.039	+		0.00326	0.00289	0.00234	0.00391	0.00483	0.00234	0.00349	0.00387	0.00296	0.00325	0.00324	0.00352	0.00474	0.00313	0.00244	0.00297	0.00345	0.00406	0.00405	0.00267	0.00339
389.18	11.544	-		0.00004	0.00002	0.00000	0.00000	0.00000	0.00000	0.00008	0.00001	0.00001	0.00008	0.00006	0.00000	0.00006	0.00001	0.00002	0.00000	0.00000	0.00000	0.00002	0.00000	0.00000
389.27	16.303	-		0.00002	0.00002	0.00001	0.00002	0.00002	0.00002	0.00002	0.00002	0.00001	0.00001	0.00000	0.00001	0.00002	0.00001	0.00003	0.00002	0.00003	0.00002	0.00000	0.00002	0.00002
396.24	14.197	-		0.00015	0.00019	0.00017	0.00016	0.00016	0.00014	0.00017	0.00018	0.00019	0.00027	0.00018	0.00017	0.00015	0.00018	0.00016	0.00018	0.00020	0.00019	0.00018	0.00015	0.00017
401.16	10.015	+		0.00039	0.00023	0.00000	0.00083	0.00083	0.00069	0.00085	0.00108	0.00076	0.00052	0.00065	0.00008	0.00015	0.00000	0.00000	0.00058	0.00008	0.00000	0.00137	0.00121	0.00116
406.07	10.402	+		0.00594	0.00596	0.00559	0.00483	0.00514	0.00384	0.00806	0.00768	0.00608	0.00622	0.00590	0.00525	0.00167	0.00137	0.00090	0.00910	0.00789	0.00709	0.00353	0.00275	0.00243
406.57	10.406	+		0.00216	0.00204	0.00218	0.00159	0.00181	0.00142	0.00299	0.00285	0.00237	0.00251	0.00201	0.00184	0.00016	0.00000	0.00000	0.00340	0.00298	0.00251	0.00108	0.00097	0.00078
407.00	8.51	+		0.00320	0.00341	0.00335	0.00336	0.00291	0.00265	0.00359	0.00391	0.00408	0.00393	0.00375	0.00365	0.00229	0.00242	0.00230	0.00374	0.00328	0.00310	0.00380	0.00363	0.00354
407.01	8.022	+		0.00270	0.00158	0.00352	0.00037	0.00198	0.00169	0.00313	0.00434	0.00362	0.00109	0.00483	0.00426	0.00080	0.00273	0.00008	0.00185	0.00248	0.00336	0.00247	0.00189	0.00249
409.16	13.042	+		0.00155	0.00146	0.00119	0.00190	0.00248	0.00115	0.00184	0.00222	0.00153	0.00179	0.00172	0.00141	0.00242	0.00157	0.00125	0.00150	0.00193	0.00210	0.00192	0.00127	0.00172
411.02	8.385	-		0.00400	0.00582	0.00398	0.00128	0.00212	0.00219	0.00173	0.00302	0.00513	0.00101	0.00343	0.00443	0.00154	0.00220	0.00120	0.00179	0.00332	0.00381	0.00345	0.00308	0.00592
413.26	15.4	-		0.00009	0.00014	0.00011	0.00016	0.00018	0.00030	0.00008	0.00010	0.00008	0.00009	0.00004	0.00009	0.00007	0.00025	0.00001	0.00017	0.00014	0.00009	0.00060	0.00003	0.00006
413.26	16.261	-		0.00025	0.00023	0.00014	0.00019	0.00022	0.00012	0.00010	0.00017	0.00005	0.00018	0.00017	0.00016	0.00006	0.00013	0.00024	0.00016	0.00014	0.00029	0.00009	0.00016	0.00013
414.07	10.116	+		0.00466	0.00452	0.00571	0.00226	0.00259	0.00314	0.00482	0.00379	0.00581	0.00504	0.00345	0.00413	0.00159	0.00208	0.00227	0.00421	0.00349	0.00540	0.00163	0.00129	0.00116
414.57	10.117	+		0.00164	0.00173	0.00209	0.00080	0.00090	0.00111	0.00170	0.00148	0.00220	0.00174	0.00111	0.00159	0.00058	0.00073	0.00084	0.00157	0.00128	0.00189	0.00058	0.00046	0.00016
417.08	10.805	-		0.00018	0.00012	0.00011	0.00008	0.00017	0.00000	0.00035	0.00023	0.00020	0.00022	0.00021	0.00021	0.00033	0.00040	0.00011	0.00047	0.00038	0.00035	0.00011	0.00011	0.00013
417.22	15.487	-		0.00014	0.00024	0.00004	0.00017	0.00015	0.00016	0.00006	0.00007	0.00009	0.00005	0.00006	0.00007	0.00004	0.00009	0.00006	0.00011	0.00005	0.00007	0.00019	0.00004	0.00008
419.19	11.178	-		0.00007	0.00004	0.00000	0.00002	0.00000	0.00000	0.00010	0.00007	0.00001	0.00000	0.00000	0.00000	0.00076	0.00129	0.00072	0.00004	0.00000	0.00000	0.00003	0.00000	0.00002
419.22	10.758	-		0.00016	0.00001	0.00008	0.00007	0.00004	0.00014	0.00036	0.00027	0.00022	0.00006	0.00000	0.00000	0.00013	0.00000	0.00008	0.00014	0.00002	0.00011	0.00013	0.00005	0.00005
425.04	8.708	-		0.00000	0.00000	0.00000	0.00193	0.00273	0.00218	0.00000	0.00008	0.00000	0.00000	0.00000	0.00000	0.00019	0.00013	0.00000	0.00000	0.00000	0.00000	0.00061	0.00065	0.00133
425.08	9.856	-		0.00153	0.00141	0.00058	0.00227	0.00165	0.00073	0.00130	0.00079	0.00058	0.00099	0.00120	0.00074	0.00000	0.00000	0.00000	0.00150	0.00113	0.00072	0.00123	0.00128	0.00114
425.09	8.166	-		0.00000	0.00003	0.00002	0.00000	0.00029	0.00002	0.00000	0.00000	0.00005	0.00000	0.00000	0.00000	0.00003	0.00002	0.00000	0.00000	0.00000	0.00001	0.00000	0.00000	0.00016
425.14	13.036	+		0.00000	0.00000	0.00000	0.00000	0.00019	0.00000	0.00000	0.00000	0.00000	0.00000	0.00000	0.00000	0.00032	0.00000	0.00000	0.00000	0.00000	0.00000	0.00000	0.00000	0.00007
427.10	9.847	+		0.00013	0.00032	0.00000	0.00014	0.00016	0.00000	0.00013	0.00000	0.00000	0.00000	0.00008	0.00000	0.00000	0.00000	0.00000	0.00000	0.00000	0.00000	0.00000	0.00029	0.00013
429.20	17.09	-		0.00002	0.00002	0.00004	0.00001	0.00003	0.00001	0.00003	0.00001	0.00000	0.00002	0.00001	0.00001	0.00001	0.00005	0.00003	0.00004	0.00004	0.00001	0.00003	0.00001	0.00002
431.08	17.061	-		0.00016	0.00018	0.00017	0.00015	0.00011	0.00023	0.00010	0.00010	0.00003	0.00020	0.00013	0.00015	0.00031	0.00023	0.00011	0.00008	0.00013	0.00018	0.00013	0.00010	0.00015
431.09	16.473	-		0.00009	0.00004	0.00010	0.00005	0.00003	0.00011	0.00011	0.00015	0.00004	0.00005	0.00010	0.00005	0.00006	0.00007	0.00020	0.00003	0.00008	0.00014	0.00003	0.00001	0.00007
431.10	10.031	-		0.00142	0.00223	0.00154	0.00127	0.00124	0.00084	0.00223	0.00274	0.00229	0.00147	0.00280	0.00294	0.00054	0.00078	0.00040	0.00165	0.00222	0.00274	0.00297	0.00339	0.00364
431.10	10.767	-		0.00025	0.00225	0.00167	0.00002	0.00000	0.00000	0.00069	0.00361	0.00000	0.00167	0.00001	0.00591	0.00000	0.00000	0.00000	0.00164	0.00138	0.00049	0.00394	0.00000	0.00000
431.17	13.021	-		0.00014	0.00009	0.00004	0.00019	0.00026	0.00012	0.00013	0.00017	0.00008	0.00010	0.00012	0.00013	0.00016	0.00014	0.00003	0.00008	0.00010	0.00015	0.00018	0.00008	0.00009
433.08	10.465	-		0.00021	0.00018	0.00032	0.00019	0.00016	0.00021	0.00030	0.00017	0.00040	0.00032	0.00017	0.00035	0.00079	0.00143	0.00110	0.00030	0.00023	0.00052	0.00009	0.00017	0.00016
433.11	9.859	-		0.00047	0.00040	0.00024	0.00033	0.00027	0.00019	0.00047	0.00055	0.00020	0.00028	0.00018	0.00000	0.00000	0.00000	0.00000	0.00008	0.00002	0.00050	0.00078	0.00026	0.00010
433.11	10.076	+		0.00428	0.00710	0.00681	0.00233	0.00407	0.00334	0.00558	0.00596	0.00759	0.00472	0.00715	0.00941	0.00223	0.00360	0.00223	0.00424	0.00499	0.00824	0.00594	0.00702	0.00825
433.17	10.509	-		0.00008	0.00009	0.00006	0.00000	0.00006	0.00008	0.00010	0.00008	0.00006	0.00000	0.00000	0.00000	0.00000	0.00000	0.00000	0.00011	0.00014	0.00000	0.00000	0.00000	0.00000
435.11	11.484	-		0.00000	0.00000	0.00000	0.00029	0.00036	0.00035	0.00000	0.00005	0.00005	0.00000	0.00023	0.00000	0.00000	0.00000	0.00000	0.00000	0.00000	0.00008	0.00121	0.00118	0.00100
435.12	10.206	-		0.00018	0.00015	0.00030	0.00003	0.00012	0.00016	0.00023	0.00022	0.00024	0.00010	0.00000	0.00023	0.00000	0.00000	0.00000	0.00015	0.00018	0.00031	0.00013	0.00002	0.00005
439.10	12.786	-		0.00032	0.00089	0.00093	0.00003	0.00017	0.00021	0.00000	0.00000	0.00028	0.00013	0.00071	0.00081	0.00001	0.00028	0.00033	0.00000	0.00000	0.00000	0.00022	0.00039	0.00044
439.15	9.8	+		0.00030	0.00055	0.00000	0.00208	0.00200	0.00198	0.00113	0.00087	0.00036	0.00056	0.00034	0.00040	0.00078	0.00015	0.00040	0.00034	0.00064	0.00057	0.00000	0.00000	0.00046
439.18	10.282	-		0.00078	0.00151	0.00101	0.00041	0.00077	0.00023	0.00009	0.00072	0.00100	0.00051	0.00125	0.00118	0.00000	0.00000	0.00000	0.00078	0.00193	0.00146	0.00008	0.00042	0.00064
439.26	16.004	-		0.00037	0.00016	0.00002	0.00013	0.00007	0.00005	0.00023	0.00009	0.00026	0.00026	0.00034	0.00011	0.00040	0.00022	0.00036	0.00053	0.00018	0.00047	0.00003	0.00021	0.00019
439.26	14.923	-		0.00015	0.00023	0.00018	0.00014	0.00010	0.00006	0.00010	0.00029	0.00014	0.00003	0.00012	0.00028	0.00002	0.00011	0.00025	0.00020	0.00052	0.00005	0.00036	0.00005	0.00039
440.26	16.201	-		0.00002	0.00003	0.00003	0.00001	0.00003	0.00001	0.00002	0.00003	0.00001	0.00004	0.00006	0.00006	0.00004	0.00002	0.00002	0.00001	0.00005	0.00004	0.00000	0.00002	0.00004
441.08	9.463	-		0.03435	0.04153	0.04070	0.04440	0.04720	0.03910	0.02870	0.03040	0.03740	0.02393	0.03289	0.03600	0.03520	0.03370	0.03510	0.03320	0.03700	0.04040	0.03070	0.03380	0.04190
441.09	12.941	-		0.00008	0.00015	0.00021	0.00018	0.00015	0.00008	0.00002	0.00002	0.00007	0.00003	0.00003	0.00017	0.00004	0.00007	0.00011	0.00002	0.00004	0.00010	0.00009	0.00002	0.00013
441.17	9.17	+		0.00237	0.00123	0.00171	0.00293	0.00266	0.00233	0.00318	0.00303	0.00232	0.00320	0.00269	0.00232	0.00219	0.00227	0.00208	0.00418	0.00367	0.00306	0.00264	0.00220	0.00194
441.26	9.463	-		0.00158	0.00193	0.00184	0.00203	0.00220	0.00181	0.00132	0.00135	0.00169	0.00103	0.00145	0.00163	0.00158	0.00152	0.00159	0.00148	0.00167	0.00188	0.00138	0.00150	0.00194
441.48	9.463	-		0.00098	0.00126	0.00122	0.00135	0.00146	0.00118	0.00084	0.00088	0.00112	0.00065	0.00094	0.00105	0.00107	0.00101	0.00102	0.00104	0.00110	0.00121	0.00090	0.00100	0.00128
442.99	6.013	-		0.00007	0.00004	0.00007	0.00040	0.00042	0.00019	0.00012	0.00001	0.00006	0.00015	0.00005	0.00006	0.00069	0.00064	0.00040	0.00024	0.00017	0.00002	0.00008	0.00000	0.00007
443.09	9.484	+		0.00907	0.01118	0.01090	0.01330	0.01400	0.01230	0.00756	0.00800	0.00991	0.00620	0.00821	0.00950	0.01050	0.00993	0.01170	0.00875	0.00988	0.01140	0.00784	0.00943	0.01150
444.97	15.331	-		0.00020	0.00024	0.00038	0.00007	0.00019	0.00023	0.00035	0.00032	0.00015	0.00013	0.00024	0.00045	0.00013	0.00016	0.00008	0.00019	0.00036	0.00014	0.00025	0.00033	0.00010
445.02	6.032	+		0.00099	0.00049	0.00030	0.00231	0.00231	0.00136	0.00129	0.00051	0.00049	0.00129	0.00035	0.00058	0.00350	0.00325	0.00349	0.00175	0.00128	0.00020	0.00059	0.00043	0.00062
445.25	17.038	+		0.00029	0.00049	0.00030	0.00000	0.00049	0.00000	0.00032	0.00064	0.00025	0.00000	0.00064	0.00055	0.00018	0.00024	0.00014	0.00039	0.00000	0.00000	0.00000	0.00053	0.00026
446.02	16.84	+		0.00000	0.00044	0.00015	0.00000	0.00046	0.00012	0.00000	0.00000	0.00033	0.00000	0.00000	0.00016	0.00040	0.00000	0.00014	0.00015	0.00000	0.00000	0.00025	0.00000	0.00044
447.06	8.25	+		0.00014	0.00030	0.00000	0.00000	0.00015	0.00000	0.00020	0.00053	0.00000	0.00000	0.00061	0.00027	0.00131	0.00058	0.00116	0.00063	0.00025	0.00000	0.00000	0.00012	0.00013
447.09	10.626	-		0.00222	0.00148	0.00148	0.00205	0.00175	0.00100	0.00399	0.00165	0.00143	0.00321	0.00171	0.00192	0.01400	0.00731	0.00467	0.00456	0.00270	0.00256	0.00140	0.00090	0.00075
447.09	8.12	+		0.00000	0.00019	0.00031	0.00000	0.00041	0.00017	0.00000	0.00000	0.00009	0.00036	0.00035	0.00000	0.00134	0.00000	0.00000	0.00000	0.00034	0.00009	0.00000	0.00026	0.00028
447.22	12.226	-		0.00077	0.00057	0.00046	0.00115	0.00090	0.00037	0.00130	0.00060	0.00049	0.00092	0.00110	0.00072	0.00069	0.00046	0.00038	0.00154	0.00101	0.00063	0.00206	0.00116	0.00090
449.08	10.169	-		0.00012	0.00000	0.00002	0.00000	0.00008	0.00009	0.00003	0.00006	0.00024	0.00011	0.00000	0.00015	0.00086	0.00104	0.00085	0.00018	0.00014	0.00012	0.00000	0.00005	0.00000
449.11	9.335	-		0.00223	0.00255	0.00232	0.00211	0.00180	0.00129	0.00294	0.00279	0.00504	0.00240	0.00328	0.00348	0.00159	0.00143	0.00102	0.00235	0.00237	0.00345	0.00437	0.00250	0.00403
449.11	10.434	+		0.00643	0.00665	0.00636	0.00435	0.00562	0.00279	0.00829	0.00647	0.00592	0.00766	0.00761	0.00712	0.00740	0.00520	0.00379	0.00963	0.00801	0.00697	0.00426	0.00363	0.00324
450.11	11.201	-		0.00000	0.00000	0.00008	0.00087	0.00077	0.00077	0.00058	0.00068	0.00073	0.00001	0.00000	0.00013	0.00166	0.00114	0.00093	0.00015	0.00000	0.00000	0.00000	0.00000	0.00000
451.10	9.844	-		0.00039	0.00048	0.00108	0.00010	0.00000	0.00032	0.00031	0.00050	0.00084	0.00050	0.00051	0.00059	0.00000	0.00005	0.00000	0.00051	0.00051	0.00099	0.00061	0.00049	0.00070
451.11	9.424	+		0.00062	0.00057	0.00097	0.00118	0.00152	0.00153	0.00000	0.00102	0.00145	0.00000	0.00053	0.00064	0.00065	0.00053	0.00000	0.00081	0.00052	0.00082	0.00039	0.00067	0.00158
451.29	17.474	-		0.00001	0.00000	0.00003	0.00000	0.00003	0.00003	0.00006	0.00002	0.00000	0.00004	0.00004	0.00000	0.00003	0.00003	0.00000	0.00004	0.00000	0.00006	0.00004	0.00004	0.00002
453.27	17.127	-		0.00001	0.00004	0.00005	0.00003	0.00002	0.00003	0.00001	0.00004	0.00002	0.00005	0.00003	0.00001	0.00003	0.00002	0.00005	0.00006	0.00002	0.00002	0.00005	0.00002	0.00002
453.27	16.111	-		0.00002	0.00014	0.00015	0.00003	0.00004	0.00005	0.00003	0.00002	0.00018	0.00011	0.00004	0.00007	0.00006	0.00013	0.00003	0.00015	0.00009	0.00002	0.00003	0.00009	0.00008
453.87	16.666	+		0.00022	0.00044	0.00000	0.00000	0.00016	0.00016	0.00045	0.00034	0.00022	0.00015	0.00051	0.00014	0.00023	0.00029	0.00010	0.00030	0.00014	0.00012	0.00023	0.00024	0.00011
455.09	10.021	-		0.00007	0.00009	0.00001	0.00401	0.00387	0.00436	0.00089	0.00102	0.00111	0.00050	0.00007	0.00005	0.00167	0.00195	0.00092	0.00015	0.00007	0.00017	0.00053	0.00094	0.00173
455.21	11.446	-		0.00007	0.00000	0.00000	0.00102	0.00090	0.00045	0.00001	0.00000	0.00000	0.00003	0.00000	0.00002	0.00037	0.00055	0.00037	0.00002	0.00000	0.00000	0.00000	0.00000	0.00000
456.07	8.752	-		0.00000	0.01236	0.01230	0.00128	0.01040	0.00191	0.00108	0.00400	0.00072	0.00813	0.00138	0.00461	0.00814	0.00848	0.00211	0.00000	0.01340	0.00343	0.00006	0.01340	0.00267
456.09	9.672	-		0.00000	0.00000	0.00000	0.00093	0.00097	0.00103	0.00136	0.00018	0.00057	0.00000	0.00000	0.00000	0.00089	0.00066	0.00023	0.00000	0.00000	0.00113	0.00000	0.00023	0.00046
456.77	16.827	+		0.00000	0.00071	0.00046	0.00000	0.00013	0.00014	0.00098	0.00026	0.00060	0.00000	0.00050	0.00012	0.00000	0.00000	0.00017	0.00021	0.00056	0.00032	0.00054	0.00038	0.00062
456.81	8.912	-		0.00051	0.00168	0.00386	0.00077	0.00085	0.00097	0.00104	0.00000	0.00082	0.00101	0.00230	0.00169	0.00295	0.00239	0.00139	0.00220	0.00391	0.00447	0.00063	0.00213	0.00326
457.07	8.917	-		0.10863	0.13601	0.12600	0.12700	0.13200	0.10300	0.10100	0.11800	0.13200	0.09022	0.12879	0.14100	0.10600	0.10078	0.10800	0.11900	0.13500	0.15100	0.11200	0.12100	0.13900
457.08	12.008	-		0.00047	0.00062	0.00031	0.00082	0.00085	0.08060	0.00076	0.00009	0.00019	0.00011	0.00014	0.00020	0.08160	0.00032	0.10800	0.00060	0.00014	0.00031	0.00005	0.04410	0.00047
457.26	8.914	-		0.00389	0.00504	0.00474	0.00455	0.00485	0.00377	0.00379	0.00444	0.00487	0.00332	0.00470	0.00520	0.00384	0.00363	0.00395	0.00444	0.00502	0.00555	0.00403	0.00437	0.00519
457.38	8.914	-		0.00162	0.00120	0.00228	0.00200	0.00329	0.00160	0.00000	0.00000	0.00099	0.00077	0.00048	0.00223	0.00095	0.00176	0.00000	0.00000	0.00109	0.00130	0.00000	0.00208	0.00232
457.48	8.912	-		0.00276	0.00347	0.00326	0.00320	0.00340	0.00256	0.00258	0.00297	0.00330	0.00250	0.00323	0.00362	0.00285	0.00250	0.00273	0.00304	0.00337	0.00381	0.00272	0.00304	0.00344
457.73	8.911	-		0.00273	0.00349	0.00333	0.00321	0.00335	0.00258	0.00266	0.00301	0.00340	0.00228	0.00335	0.00362	0.00271	0.00250	0.00273	0.00307	0.00343	0.00384	0.00184	0.00303	0.00350

(continued)

			(Name)	AT1	AT2	AT3	BF1	BF2	BF3	KM1	KM2	KM3	SM1	SM2	SM3	SR1	SR2	SR3	YB1	YB2	YB3	YM1	YM2	YM3
			(ORAC)	35,647	38,661	51,482	42,552	65,085	55,612	44,400	43,107	48,544	25,445	40,191	46,160	37,930	42,436	38,953	34,246	36,545	41,729	37,746	43,496	47,566
m/z	R.T.	Polar	olyphenol)	1.93	2.11	2.54	2.04	2.56	2.54	2.00	2.11	2.41	1.96	2.13	2.33	2.44	2.33	2.17	2.12	2.13	2.34	2.21	2.28	2.65
458.61	10.963	-		0.00000	0.00001	0.00015	0.00000	0.00000	0.00000	0.00012	0.00007	0.00066	0.00000	0.00000	0.00003	0.00000	0.00066	0.00025	0.00000	0.00000	0.00000	0.00000	0.00000	0.00000
459.09	8.914	+		0.05034	0.06195	0.05710	0.05680	0.05690	0.04770	0.04570	0.05550	0.05880	0.04328	0.05691	0.06350	0.05310	0.04898	0.05730	0.05430	0.06330	0.06750	0.04850	0.05560	0.06040
461.16	9.975	-		0.00024	0.00032	0.00021	0.00127	0.00125	0.00119	0.00000	0.00017	0.00024	0.00002	0.00024	0.00027	0.00063	0.00013	0.00000	0.00022	0.00069	0.00055	0.00009	0.00019	0.00019
461.23	11.606	-		0.00008	0.00020	0.00012	0.00004	0.00019	0.00019	0.00003	0.00012	0.00002	0.00000	0.00001	0.00003	0.00001	0.00013	0.00003	0.00002	0.00009	0.00001	0.00000	0.00003	0.00008
462.67	16.691	+		0.00041	0.00050	0.00000	0.00025	0.00022	0.00071	0.00031	0.00023	0.00059	0.00000	0.00018	0.00021	0.00000	0.00018	0.00031	0.00024	0.00012	0.00017	0.00000	0.00023	0.00076
463.08	10.291	-		0.00279	0.00273	0.00393	0.00175	0.00181	0.00180	0.00350	0.00155	0.00294	0.00485	0.00237	0.00402	0.01570	0.01513	0.01370	0.00288	0.00197	0.00316	0.00156	0.00120	0.00141
463.22	10.874	-		0.00021	0.00026	0.00008	0.00024	0.00038	0.00022	0.00026	0.00024	0.00014	0.00015	0.00031	0.00012	0.00029	0.00053	0.00043	0.00023	0.00027	0.00009	0.00024	0.00006	0.00007
463.24	11.602	-		0.00031	0.00029	0.00020	0.00034	0.00063	0.00041	0.00014	0.00008	0.00014	0.00016	0.00035	0.00010	0.00000	0.00004	0.00000	0.00014	0.00008	0.00006	0.00058	0.00044	0.00032
463.26	10.305	-		0.00012	0.00017	0.00020	0.00002	0.00002	0.00009	0.00017	0.00008	0.00008	0.00019	0.00010	0.00019	0.00066	0.00062	0.00058	0.00007	0.00003	0.00007	0.00002	0.00001	0.00000
465.09	8.308	+		0.00000	0.00000	0.00000	0.00000	0.00000	0.00000	0.00000	0.00000	0.00000	0.00000	0.00000	0.00019	0.01010	0.00589	0.00574	0.00000	0.00000	0.00000	0.00000	0.00000	0.00000
465.10	10.166	+		0.00367	0.00366	0.00572	0.00157	0.00199	0.00225	0.00330	0.00212	0.00441	0.00508	0.00287	0.00430	0.00561	0.00608	0.00558	0.00298	0.00222	0.00364	0.00152	0.00120	0.00121
467.12	7.846	-		0.00018	0.00015	0.00030	0.00001	0.00000	0.00007	0.00010	0.00015	0.00024	0.00011	0.00009	0.00011	0.00007	0.00004	0.00006	0.00015	0.00008	0.00020	0.00021	0.00015	0.00018
467.13	11.75	-		0.00081	0.00036	0.00027	0.00017	0.00030	0.00012	0.00059	0.00059	0.00054	0.00076	0.00089	0.00136	0.00052	0.00012	0.00001	0.00103	0.00096	0.00073	0.00000	0.00000	0.00000
467.78	16.812	+		0.00026	0.00038	0.00016	0.00030	0.00047	0.00023	0.00054	0.00043	0.00044	0.00000	0.00060	0.00008	0.00039	0.00017	0.00058	0.00035	0.00062	0.00000	0.00031	0.00050	0.00034
468.03	16.761	+		0.00067	0.00060	0.00037	0.00078	0.00036	0.00043	0.00032	0.00055	0.00017	0.00000	0.00014	0.00060	0.00077	0.00037	0.00035	0.00061	0.00024	0.00000	0.00038	0.00075	0.00090
468.13	12.011	-		0.00040	0.00021	0.00022	0.00018	0.00024	0.00010	0.00024	0.00025	0.00036	0.00042	0.00033	0.00045	0.00027	0.00017	0.00028	0.00052	0.00057	0.00042	0.00032	0.00017	0.00045
469.13	9.527	+		0.00396	0.00254	0.00155	0.00258	0.00307	0.00244	0.00140	0.00245	0.00096	0.00197	0.00202	0.00143	0.00094	0.00144	0.00106	0.00187	0.00265	0.00151	0.00227	0.00275	0.00140
470.21	17.358	-		0.00004	0.00006	0.00004	0.00003	0.00005	0.00003	0.00002	0.00001	0.00005	0.00005	0.00003	0.00003	0.00002	0.00000	0.00001	0.00006	0.00005	0.00003	0.00008	0.00005	0.00005
471.08	9.368	-		0.00016	0.00027	0.00000	0.01010	0.00853	0.00859	0.00242	0.00282	0.00451	0.00002	0.00064	0.00021	0.00534	0.00613	0.00277	0.00081	0.00024	0.00064	0.00177	0.00238	0.00394
471.09	10.606	+		0.00063	0.00022	0.00038	0.00057	0.00050	0.00024	0.00123	0.00042	0.00034	0.00088	0.00043	0.00044	0.00452	0.00227	0.00173	0.00137	0.00075	0.00066	0.00038	0.00016	0.00000
471.22	12.239	+		0.00071	0.00061	0.00035	0.00143	0.00109	0.00030	0.00107	0.00048	0.00053	0.00055	0.00109	0.00055	0.00057	0.00019	0.00000	0.00169	0.00059	0.00034	0.00281	0.00152	0.00118
471.28	16.595	+		0.00000	0.00000	0.00009	0.00000	0.00008	0.00023	0.00000	0.00000	0.00038	0.00000	0.00000	0.00021	0.00052	0.00027	0.00000	0.00017	0.00017	0.00000	0.00028	0.00007	0.00052
471.39	17.866	+		0.00039	0.00060	0.00043	0.00038	0.00048	0.00012	0.00000	0.00010	0.00000	0.00058	0.00017	0.00032	0.00026	0.00018	0.00000	0.00044	0.00000	0.00000	0.00052	0.00000	0.00051
471.48	16.703	+		0.00022	0.00089	0.00057	0.00000	0.00044	0.00054	0.00042	0.00027	0.00109	0.00000	0.00000	0.00063	0.00077	0.00174	0.00062	0.00069	0.00039	0.00054	0.00119	0.00052	0.00000
473.15	2.044	-		0.00035	0.00028	0.00029	0.00000	0.00006	0.00016	0.00053	0.00046	0.00036	0.00034	0.00037	0.00036	0.00034	0.00049	0.00053	0.00063	0.00050	0.00044	0.00036	0.00026	0.00025
473.22	16.922	-		0.00005	0.00000	0.00005	0.00003	0.00002	0.00001	0.00005	0.00001	0.00006	0.00001	0.00003	0.00007	0.00003	0.00001	0.00001	0.00003	0.00002	0.00001	0.00002	0.00003	0.00003
473.38	17.745	-		0.00010	0.00009	0.00068	0.00005	0.00001	0.00011	0.00010	0.00001	0.00065	0.00032	0.00008	0.00009	0.00025	0.00020	0.00016	0.00073	0.00053	0.00016	0.00001	0.00071	0.00032
473.38	16.829	-		0.00001	0.00001	0.00000	0.00003	0.00000	0.00003	0.00000	0.00004	0.00013	0.00001	0.00007	0.00000	0.00005	0.00000	0.00002	0.00008	0.00001	0.00015	0.00000	0.00000	0.00000
474.38	17.566	-		0.00023	0.00008	0.00002	0.00015	0.00011	0.00014	0.00001	0.00006	0.00002	0.00005	0.00002	0.00004	0.00002	0.00010	0.00004	0.00005	0.00005	0.00000	0.00003	0.00003	0.00002
477.07	10.102	-		0.00026	0.00031	0.00018	0.00092	0.00052	0.00004	0.00032	0.00003	0.00006	0.00030	0.00034	0.00040	0.00079	0.00072	0.00074	0.00026	0.00021	0.00000	0.00012	0.00015	0.00012
478.08	9.92	-		0.00018	0.00028	0.00050	0.00033	0.00029	0.00017	0.00019	0.00006	0.00041	0.00026	0.00010	0.00042	0.00115	0.00120	0.00123	0.00007	0.00006	0.00051	0.00006	0.00000	0.00026
478.78	16.784	+		0.00030	0.00057	0.00046	0.00107	0.00085	0.00109	0.00021	0.00068	0.00129	0.00031	0.00069	0.00055	0.00076	0.00050	0.00102	0.00106	0.00032	0.00029	0.00064	0.00055	0.00068
478.97	15.803	-		0.00012	0.00008	0.00014	0.00008	0.00006	0.00011	0.00012	0.00009	0.00018	0.00011	0.00006	0.00014	0.00018	0.00007	0.00003	0.00011	0.00013	0.00004	0.00002	0.00011	0.00007
479.04	16.78	+		0.00049	0.00100	0.00098	0.00090	0.00060	0.00073	0.00026	0.00093	0.00066	0.00016	0.00031	0.00100	0.00074	0.00089	0.00096	0.00094	0.00000	0.00067	0.00095	0.00067	0.00066
479.08	9.902	-		0.00452	0.00550	0.01040	0.00231	0.00351	0.00448	0.00419	0.00297	0.00837	0.00722	0.00489	0.01100	0.02080	0.02112	0.02170	0.00430	0.00447	0.01000	0.00337	0.00285	0.00494
479.26	10.054	-		0.00016	0.00012	0.00033	0.00000	0.00004	0.00016	0.00010	0.00014	0.00035	0.00028	0.00013	0.00043	0.00084	0.00087	0.00089	0.00009	0.00022	0.00043	0.00003	0.00000	0.00019
480.08	16.632	+		0.00054	0.00026	0.00015	0.00073	0.00053	0.00057	0.00014	0.00040	0.00013	0.00000	0.00011	0.00036	0.00007	0.00062	0.00025	0.00021	0.00013	0.00033	0.00038	0.00018	0.00011
480.28	16.7	+		0.00000	0.00061	0.00011	0.00039	0.00017	0.00022	0.00018	0.00000	0.00036	0.00107	0.00000	0.00026	0.00015	0.00046	0.00014	0.00072	0.00029	0.00046	0.00015	0.00059	0.00055
481.10	9.905	+		0.00129	0.00124	0.00243	0.00072	0.00106	0.00130	0.00120	0.00095	0.00228	0.00208	0.00129	0.00272	0.00464	0.00536	0.00540	0.00130	0.00117	0.00259	0.00086	0.00078	0.00116
483.13	11.389	-		0.00170	0.00303	0.00146	0.00031	0.00039	0.00011	0.00101	0.00149	0.00087	0.00121	0.00204	0.00162	0.00034	0.00013	0.00000	0.00195	0.00260	0.00117	0.00000	0.00000	0.00000
487.09	10.315	+		0.00029	0.00073	0.00083	0.00000	0.00000	0.00034	0.00087	0.00000	0.00032	0.00164	0.00024	0.00034	0.00625	0.00419	0.00473	0.00090	0.00000	0.00065	0.00000	0.00000	0.00012
487.21	10.809	+		0.00263	0.00264	0.00181	0.00310	0.00393	0.00218	0.00238	0.00246	0.00182	0.00142	0.00168	0.00144	0.00402	0.00662	0.00632	0.00305	0.00256	0.00159	0.00100	0.00103	0.00146
488.89	16.622	+		0.00088	0.00011	0.00022	0.00019	0.00037	0.00062	0.00026	0.00063	0.00013	0.00000	0.00019	0.00000	0.00025	0.00082	0.00020	0.00031	0.00023	0.00000	0.00066	0.00053	0.00018
489.08	16.647	+		0.00068	0.00118	0.00000	0.00000	0.00093	0.00035	0.00104	0.00043	0.00009	0.00000	0.00068	0.00009	0.00067	0.00014	0.00113	0.00048	0.00074	0.00046	0.00033	0.00035	0.00016
489.10	9.866	-		0.00000	0.00000	0.00000	0.00000	0.00011	0.00000	0.00017	0.00035	0.00018	0.00000	0.00024	0.00000	0.00000	0.00000	0.00000	0.00018	0.00029	0.00012	0.00012	0.00021	0.00024
489.10	10.954	-		0.00008	0.00008	0.00011	0.00000	0.00000	0.00000	0.00006	0.00003	0.00002	0.00013	0.00016	0.00014	0.00731	0.00471	0.00474	0.00004	0.00003	0.00000	0.00000	0.00003	0.00000
489.29	16.681	+		0.00045	0.00023	0.00028	0.00000	0.00014	0.00000	0.00000	0.00037	0.00093	0.00000	0.00015	0.00016	0.00014	0.00034	0.00022	0.00092	0.00026	0.00000	0.00000	0.00093	0.00027
489.79	16.757	+		0.00063	0.00050	0.00029	0.00095	0.00085	0.00040	0.00115	0.00060	0.00093	0.00073	0.00111	0.00049	0.00097	0.00059	0.00084	0.00076	0.00113	0.00079	0.00118	0.00049	0.00050
490.04	16.787	+		0.00158	0.00090	0.00075	0.00049	0.00157	0.00128	0.00119	0.00127	0.00140	0.00061	0.00198	0.00073	0.00098	0.00043	0.00080	0.00136	0.00026	0.00203	0.00173	0.00072	0.00055
490.29	16.763	+		0.00050	0.00038	0.00033	0.00029	0.00056	0.00036	0.00026	0.00054	0.00023	0.00029	0.00034	0.00013	0.00031	0.00039	0.00007	0.00049	0.00013	0.00048	0.00043	0.00018	0.00032
491.29	16.855	+		0.00026	0.00000	0.00059	0.00000	0.00012	0.00034	0.00016	0.00009	0.00065	0.00020	0.00000	0.00000	0.00000	0.00000	0.00000	0.00044	0.00036	0.00039	0.00021	0.00031	0.00049
493.22	12.231	-		0.00357	0.00294	0.00242	0.00629	0.00466	0.00210	0.00500	0.00265	0.00243	0.00437	0.00485	0.00350	0.00315	0.00216	0.00186	0.00701	0.00425	0.00291	0.01100	0.00630	0.00483
497.69	16.631	+		0.00067	0.00022	0.00022	0.00000	0.00015	0.00048	0.00034	0.00041	0.00070	0.00000	0.00040	0.00013	0.00057	0.00000	0.00016	0.00070	0.00022	0.00028	0.00086	0.00084	0.00052
497.89	16.632	+		0.00054	0.00055	0.00000	0.00000	0.00028	0.00030	0.00017	0.00056	0.00067	0.00050	0.00015	0.00015	0.00023	0.00076	0.00046	0.00000	0.00061	0.00025	0.00027	0.00021	0.00043
499.12	10.336	-		0.00037	0.00042	0.00057	0.00023	0.00021	0.00003	0.00029	0.00045	0.00026	0.00026	0.00056	0.00041	0.00000	0.00000	0.00000	0.00064	0.00079	0.00049	0.00000	0.00000	0.00000
500.80	16.751	+		0.00176	0.00144	0.00179	0.00160	0.00157	0.00083	0.00098	0.00080	0.00009	0.00115	0.00112	0.00042	0.00175	0.00034	0.00089	0.00187	0.00167	0.00056	0.00205	0.00155	0.00163
501.05	16.748	+		0.00101	0.00086	0.00060	0.00094	0.00146	0.00063	0.00204	0.00234	0.00139	0.00068	0.00000	0.00230	0.00090	0.00094	0.00240	0.00115	0.00031	0.00116	0.00047	0.00094	0.00053
501.30	16.752	+		0.00105	0.00024	0.00020	0.00017	0.00022	0.00035	0.00038	0.00068	0.00024	0.00000	0.00011	0.00017	0.00061	0.00038	0.00022	0.00095	0.00028	0.00052	0.00023	0.00028	0.00049
503.08	9.901	+		0.00042	0.00038	0.00068	0.00019	0.00033	0.00033	0.00046	0.00011	0.00077	0.00065	0.00020	0.00067	0.00157	0.00127	0.00155	0.00045	0.00035	0.00085	0.00009	0.00000	0.00035
504.07	9.432	-		0.00096	0.00121	0.00072	0.00100	0.00086	0.00095	0.00050	0.00058	0.00137	0.00038	0.00062	0.00028	0.00053	0.00090	0.00061	0.00000	0.00030	0.00033	0.00087	0.00124	0.00143
505.09	10.545	-		0.00000	0.00000	0.00001	0.00000	0.00000	0.00000	0.00000	0.00000	0.00000	0.00000	0.00000	0.00000	0.00418	0.00576	0.00742	0.00000	0.00000	0.00000	0.00000	0.00000	0.00000
505.97	16.84	+		0.00000	0.00000	0.00000	0.00000	0.00006	0.00000	0.00000	0.00000	0.00000	0.00000	0.00000	0.00000	0.00016	0.00009	0.00000	0.00000	0.00008	0.00000	0.00000	0.00035	0.00029
506.23	16.227	-		0.00002	0.00004	0.00003	0.00003	0.00006	0.00004	0.00002	0.00004	0.00001	0.00005	0.00003	0.00003	0.00002	0.00007	0.00001	0.00001	0.00001	0.00000	0.00003	0.00002	0.00003
506.30	16.806	+		0.00038	0.00008	0.00022	0.00000	0.00012	0.00035	0.00030	0.00020	0.00062	0.00000	0.00015	0.00024	0.00064	0.00055	0.00015	0.00074	0.00094	0.00014	0.00000	0.00051	0.00053
506.50	16.638	+		0.00000	0.00015	0.00000	0.00000	0.00038	0.00020	0.00000	0.00000	0.00000	0.00000	0.00017	0.00007	0.00012	0.00016	0.00063	0.00014	0.00000	0.00024	0.00000	0.00008	0.00000
506.70	16.613	+		0.00018	0.00021	0.00000	0.00022	0.00030	0.00033	0.00041	0.00054	0.00065	0.00000	0.00045	0.00011	0.00049	0.00035	0.00021	0.00035	0.00040	0.00032	0.00015	0.00045	0.00036
507.20	11.792	-		0.00009	0.00004	0.00000	0.00018	0.00010	0.00000	0.00042	0.00026	0.00000	0.00010	0.00008	0.00001	0.00003	0.00001	0.00000	0.00028	0.00009	0.00000	0.00005	0.00000	0.00001
509.22	10.796	-		0.00133	0.00132	0.00079	0.00166	0.00186	0.00088	0.00151	0.00123	0.00079	0.00056	0.00075	0.00053	0.00155	0.00276	0.00224	0.00181	0.00161	0.00069	0.00060	0.00042	0.00068
511.05	12.405	-		0.00000	0.00002	0.00000	0.00029	0.00072	0.00093	0.00022	0.00063	0.00061	0.00024	0.00062	0.00164	0.00046	0.00086	0.00092	0.00013	0.00048	0.00098	0.00000	0.00000	0.00000
511.05	10.253	-		0.00000	0.00032	0.00012	0.00000	0.00010	0.00000	0.00025	0.00000	0.00000	0.00000	0.00026	0.00057	0.00000	0.00036	0.00000	0.00036	0.00000	0.00005	0.00001	0.00017	0.00000
511.80	16.729	+		0.00266	0.00272	0.00266	0.00154	0.00248	0.00114	0.00163	0.00057	0.00264	0.00079	0.00257	0.00240	0.00035	0.00302	0.00179	0.00206	0.00230	0.00200	0.00317	0.00159	0.00245
512.05	16.729	+		0.00049	0.00046	0.00040	0.00092	0.00100	0.00120	0.00130	0.00085	0.00121	0.00014	0.00112	0.00124	0.00142	0.00104	0.00085	0.00088	0.00105	0.00062	0.00126	0.00094	0.00080
512.30	16.73	+		0.00108	0.00081	0.00082	0.00062	0.00068	0.00012	0.00120	0.00181	0.00038	0.00099	0.00128	0.00083	0.00096	0.00103	0.00011	0.00082	0.00076	0.00071	0.00033	0.00047	0.00053
515.20	2.082	-		0.00030	0.00000	0.00000	0.00005	0.00000	0.00000	0.00015	0.00000	0.00000	0.00036	0.00000	0.00000	0.00000	0.00000	0.00000	0.00023	0.00000	0.00000	0.00024	0.00010	0.00000
516.13	11.309	-		0.00003	0.00048	0.00037	0.00286	0.00443	0.00524	0.00000	0.00000	0.00220	0.00036	0.00415	0.00055	0.00000	0.00000	0.00000	0.00019	0.00001	0.00308	0.01690	0.01690	0.01750
516.63	11.303	-		0.00000	0.00027	0.00020	0.00149	0.00237	0.00276	0.00000	0.00000	0.00114	0.00020	0.00209	0.00026	0.00000	0.00000	0.00000	0.00005	0.00000	0.00164	0.00907	0.00895	0.00924
519.39	17.556	-		0.00109	0.00096	0.00080	0.00010	0.00159	0.00134	0.00022	0.00121	0.00057	0.00075	0.00047	0.00037	0.00137	0.00121	0.00052	0.00108	0.00094	0.00064	0.00060	0.00045	0.00118
522.81	16.76	+		0.00077	0.00115	0.00081	0.00083	0.00076	0.00080	0.00142	0.00225	0.00096	0.00104	0.00223	0.00064	0.00031	0.00053	0.00115	0.00059	0.00039	0.00178	0.00040	0.00123	0.00063
523.06	16.699	+		0.00103	0.00156	0.00082	0.00099	0.00112	0.00081	0.00104	0.00211	0.00096	0.00079	0.00029	0.00048	0.00114	0.00051	0.00118	0.00098	0.00057	0.00138	0.00096	0.00078	0.00059
523.14	11.193	-		0.00000	0.00000	0.00000	0.00988	0.01010	0.00667	0.00000	0.00001	0.00001	0.00000	0.00000	0.00000	0.00000	0.00000	0.00000	0.00000	0.00000	0.00000	0.00000	0.00000	0.00000
523.32	16.72	+		0.00000	0.00086	0.00056	0.00000	0.00033	0.00050	0.00066	0.00134	0.00099	0.00010	0.00042	0.00035	0.00000	0.00011	0.00000	0.00041	0.00075	0.00022	0.00037	0.00070	0.00028
523.40	17.318	+		0.00000	0.00068	0.00112	0.00000	0.00054	0.00028	0.00093	0.00095	0.00099	0.00167	0.00062	0.00038	0.00073	0.00034	0.00075	0.00064	0.00183	0.00104	0.00024	0.00066	0.00182
523.64	11.16	-		0.00000	0.00000	0.00000	0.00532	0.00563	0.00373	0.00000	0.00000	0.00000	0.00000	0.00000	0.00000	0.00000	0.00000	0.00000	0.00000	0.00000	0.00000	0.00000	0.00000	0.00000
524.13	11.003	-		0.00005	0.00051	0.00086	0.00304	0.00391	0.00473	0.00000	0.00000	0.00205	0.00048	0.00171	0.00128	0.00000	0.00000	0.00000	0.00006	0.00000	0.00254	0.00719	0.00744	0.00926
524.41	17.337	+		0.00057	0.00038	0.00050	0.00022	0.00000	0.00051	0.00026	0.00018	0.00027	0.00012	0.00036	0.00037	0.00075	0.00063	0.00012	0.00061	0.00034	0.00068	0.00027	0.00072	0.00053
524.63	10.999	-		0.00000	0.00020	0.00036	0.00079	0.00112	0.00172	0.00000	0.00000	0.00091	0.00017	0.00078	0.00058	0.00000	0.00000	0.00000	0.00001	0.00000	0.00104	0.00327	0.00345	0.00409
529.06	10.561	-		0.00048	0.00007	0.00003	0.00144	0.00174	0.00208	0.00115	0.00175	0.00260	0.00098	0.00221	0.00464	0.00210	0.00207	0.00261	0.00085	0.00164	0.00293	0.00000	0.00000	0.00006
530.91	17.38	+		0.00081	0.00087	0.00097	0.00000	0.00071	0.00055	0.00133	0.00135	0.00045	0.00103	0.00030	0.00115	0.00095	0.00054	0.00047	0.00081	0.00086	0.00089	0.00033	0.00092	0.00108
531.08	7.971	-		0.00006	0.00012	0.00023	0.00009	0.00011	0.00029	0.00000	0.00000	0.00030	0.00001	0.00008	0.00022	0.00019	0.00083	0.00061	0.00004	0.00014	0.00033	0.00001	0.00005	0.00014
531.14	11.069	-		0.00000	0.00000	0.00000	0.00748	0.01030	0.01320	0.00000	0.00000	0.00021	0.00000	0.00000	0.00083	0.00945	0.01068	0.00875	0.00000	0.00000	0.00000	0.00000	0.00000	0.00000
531.41	17.449	+		0.00049	0.00021	0.00000	0.00071	0.00045	0.00055	0.00261	0.00314	0.00041	0.00130	0.00183	0.00152	0.00089	0.00107	0.00146	0.00042	0.00062	0.00147	0.00041	0.00074	0.00099
531.64	11.073	-		0.00000	0.00000	0.00000	0.00375	0.00529	0.00679	0.00000	0.00000	0.00005	0.00000	0.00000	0.00035	0.00645	0.00519	0.00415	0.00000	0.00000	0.00000	0.00000	0.00000	0.00000
531.91	17.349	+		0.00016	0.00046	0.00024	0.00000	0.00000	0.00046	0.00060	0.00094	0.00044	0.00000	0.00006	0.00031	0.00022	0.00000	0.00037	0.00051	0.00042	0.00024	0.00000	0.00061	0.00103
532.08	8.059	-		0.00000	0.00005	0.00012	0.00000	0.00003	0.00016	0.00000	0.00000	0.00011	0.00001	0.00000	0.00002	0.00152	0.00089	0.00093	0.00000	0.00000	0.00003	0.00000	0.00000	0.00002
533.13	10.045	-		0.00138	0.00174	0.00151	0.00017	0.00029	0.00030	0.00164	0.00260	0.00164	0.00119	0.00183	0.00186	0.00023	0.00061	0.00046	0.00165	0.00243	0.00223	0.00075	0.00076	0.00083
533.82	16.685	+		0.00092	0.00149	0.00156	0.00154	0.00188	0.00147	0.00174	0.00161	0.00134	0.00083	0.00170	0.00079	0.00068	0.00188	0.00064	0.00140	0.00225	0.00026	0.00000	0.00197	0.00066
534.07	16.729	+		0.00050	0.00118	0.00124	0.00103	0.00000	0.00035	0.00169	0.00105	0.00062	0.00202	0.00109	0.00114	0.00039	0.00102	0.00074	0.00080	0.00069	0.00063	0.00085	0.00125	0.00126
534.32	16.679	+		0.00073	0.00045	0.00026	0.00000	0.00038	0.00055	0.00094	0.00028	0.00000	0.00000	0.00052	0.00030	0.00035	0.00044	0.00055	0.00032	0.00043	0.00058	0.00022	0.00043	0.00037
535.14	10.063	+		0.00391	0.00464	0.00556	0.00020	0.00075	0.00070	0.00291	0.00589	0.00491	0.00243	0.00449	0.00563	0.00108	0.00215	0.00166	0.00426	0.00538	0.00655	0.00089	0.00155	0.00177
535.32	16.826	+		0.00000	0.00000	0.00117	0.00000	0.00039	0.00000	0.00000	0.00015	0.00055	0.00000	0.00000	0.00016	0.00036	0.00063	0.00006	0.00067	0.00058	0.00000	0.00000	0.00048	0.00056
537.42	17.483	+		0.00027	0.00019	0.00012	0.00000	0.00000	0.00000	0.00068	0.00039	0.00008	0.00000	0.00039	0.00052	0.00031	0.00020	0.00026	0.00066	0.00057	0.00079	0.00014	0.00088	0.00045
539.13	10.789	-		0.00000	0.00000	0.00000	0.00040	0.00055	0.00076	0.00000	0.00000	0.00000	0.00000	0.00000	0.00027	0.00563	0.01079	0.01270	0.00000	0.00000	0.00000	0.00000	0.00000	0.00000
539.64	10.785	-		0.00000	0.00000	0.00000	0.00016	0.00031	0.00041	0.00000	0.00000	0.00000	0.00000	0.00000	0.00011	0.00302	0.00575	0.00679	0.00000	0.00000	0.00000	0.00000	0.00000	0.00000
543.02	9.483	+		0.00072	0.00142	0.00131	0.00099	0.00096	0.00137	0.00095	0.00000	0.00127	0.00055	0.00061	0.00103	0.00126	0.00122	0.00146	0.00069	0.00074	0.00141	0.00094	0.00078	0.00126
544.82	16.721	+		0.00062	0.00120	0.00033	0.00081	0.00136	0.00082	0.00075	0.00075	0.00098	0.00106	0.00028	0.00050	0.00024	0.00078	0.00022	0.00034	0.00030	0.00038	0.00118	0.00083	0.00079
544.96	15.952	-		0.00001	0.00001	0.00004	0.00002	0.00003	0.00001	0.00001	0.00002	0.00000	0.00004	0.00003	0.00003	0.00006	0.00002	0.00009	0.00002	0.00002	0.00000	0.00000	0.00003	0.00001
545.07	16.681	+		0.00144	0.00088	0.00172	0.00043	0.00126	0.00048	0.00101	0.00135	0.00317	0.00175	0.00155	0.00139	0.00161	0.00118	0.00081	0.00151	0.00196	0.00090	0.00098	0.00105	0.00143
545.11	11.341	+		0.00000	0.00000	0.00000	0.00051	0.00069	0.00119	0.00000	0.00000	0.00041	0.00000	0.00095	0.00000	0.00000	0.00000	0.00000	0.00000	0.00000	0.00061	0.00409	0.00458	0.00445
545.32	16.684	+		0.00056	0.00131	0.00083	0.00000	0.00054	0.00033	0.00016	0.00105	0.00060	0.00043	0.00014	0.00013	0.00000	0.00101	0.00045	0.00022	0.00090	0.00034	0.00044	0.00071	0.00103
551.10	10.55	+		0.00000	0.00000	0.00000	0.00000	0.00000	0.00000	0.00000	0.00000	0.00000	0.00000	0.00000	0.00000	0.00205	0.00308	0.00311	0.00000	0.00000	0.00000	0.00000	0.00000	0.00000
551.27	11.695	+		0.00005	0.00023	0.00008	0.00008	0.00036	0.00000	0.00008	0.00045	0.00019	0.00021	0.00009	0.00009	0.00029	0.00000	0.00041	0.00011	0.00000	0.00023	0.00014	0.00000	0.00005
553.10	11.027	+		0.00000	0.00000	0.00000	0.00014	0.00086	0.00138	0.00000	0.00000	0.00071	0.00000	0.00047	0.00010	0.00000	0.00000	0.00000	0.00000	0.00000	0.00064	0.00246	0.00278	0.00360
555.03	8.926	-		0.00305	0.00578	0.00619	0.00117	0.00000	0.00449	0.00186	0.00109	0.00409	0.00391	0.00408	0.00401	0.00360	0.00451	0.00403	0.00000	0.00251	0.00137	0.00430	0.00115	0.00566
555.83	16.67	+		0.00038	0.00044	0.00029	0.00038	0.00049	0.00063	0.00042	0.00061	0.00033	0.00024	0.00026	0.00026	0.00016	0.00048	0.00060	0.00068	0.00049	0.00059	0.00047	0.00040	0.00057
556.08	16.672	+		0.00033	0.00072	0.00024	0.00044	0.00036	0.00056	0.00086	0.00000	0.00097	0.00033	0.00045	0.00050	0.00054	0.00051	0.00036	0.00062	0.00116	0.00027	0.00026	0.00074	0.00050
556.33	16.664	+		0.00018	0.00000	0.00033	0.00000	0.00013	0.00012	0.00013	0.00061	0.00042	0.00000	0.00022	0.00017	0.00081	0.00014	0.00045	0.00077	0.00010	0.00041	0.00000	0.00027	0.00059
559.01	8.933	+		0.00196	0.00334	0.00286	0.00328	0.00283	0.00266	0.00307	0.00346	0.00323	0.00293	0.00345	0.00346	0.00310	0.00304	0.00339	0.00327	0.00228	0.00369	0.00291	0.00312	0.00294
560.33	16.734	+		0.00017	0.00020	0.00015	0.00011	0.00022	0.00035	0.00008	0.00013	0.00000	0.00000	0.00000	0.00009	0.00019	0.00028	0.00009	0.00040	0.00033	0.00008	0.00044	0.00010	0.00011
563.14	9.646	-		0.00858	0.01150	0.01110	0.00228	0.00261	0.00257	0.00898	0.01100	0.00948	0.01008	0.01241	0.01280	0.00142	0.00251	0.00146	0.00871	0.01100	0.01190	0.00639	0.00625	0.00705
563.20	9.65	+		0.00185	0.00173	0.00000	0.00313	0.00220	0.00157	0.00233	0.00202	0.00162	0.00200	0.00225	0.00000	0.00168	0.00213	0.00174	0.00287	0.00123	0.00000	0.00477	0.00385	0.00312
565.04	2.897	-		0.00059	0.00048	0.00038	0.00053	0.00033	0.00017	0.00067	0.00051	0.00044	0.00052	0.00058	0.00035	0.00047	0.00026	0.00027	0.00067	0.00050	0.00039	0.00066	0.00053	0.00027
565.15	9.616	+		0.01701	0.02221	0.02280	0.00699	0.00889	0.00795	0.01800	0.02070	0.02100	0.01978	0.02290	0.02600	0.00367	0.00661	0.00412	0.01920	0.02250	0.02700	0.01210	0.01420	0.01620
567.09	16.647	+		0.00000	0.00114	0.00014	0.00040	0.00075	0.00041	0.00052	0.00031	0.00048	0.00037	0.00043	0.00041	0.00066	0.00033	0.00047	0.00016	0.00045	0.00015	0.00106	0.00063	0.00066
567.20	10.163	-		0.00002	0.00002	0.00000	0.00006	0.00004	0.00003	0.00000	0.00003	0.00000	0.00001	0.00005	0.00000	0.00000	0.00000	0.00000	0.00010	0.00003	0.00000	0.00019	0.00014	0.00012
567.33	16.687	+		0.00033	0.00027	0.00023	0.00000	0.00000	0.00041	0.00019	0.00047	0.00021	0.00000	0.00058	0.00070	0.00059	0.00028	0.00052	0.00022	0.00015	0.00055	0.00034	0.00039	0.00046
568.29	11.698	+		0.00164	0.00306	0.00186	0.00204	0.00327	0.00116	0.00164	0.00320	0.00196	0.00203	0.00202	0.00207	0.00309	0.00101	0.00246	0.00238	0.00128	0.00239	0.00223	0.00143	0.00186
573.25	11.695	+		0.00136	0.00254	0.00157	0.00158	0.00256	0.00091	0.00133	0.00263	0.00164	0.00170	0.00173	0.00184	0.00264	0.00076	0.00214	0.00198	0.00103	0.00210	0.00180	0.00114	0.00155
576.12	8.626	-		0.00036	0.00037	0.00053	0.00079	0.00092	0.00061	0.00086	0.00085	0.00058	0.00076	0.00050	0.00052	0.00111	0.00184	0.00090	0.00140	0.00086	0.00052	0.00093	0.00046	0.00081
576.62	8.471	-		0.00022	0.00013	0.00023	0.00063	0.00066	0.00041	0.00047	0.00044	0.00025	0.00024	0.00030	0.00031	0.00056	0.00090	0.00053	0.00048	0.00042	0.00033	0.00038	0.00023	0.00029
577.13	8.393	-		0.00398	0.00344	0.00335	0.00786	0.00794	0.00462	0.00672	0.00636	0.00399	0.00556	0.00476	0.00443	0.00503	0.00614	0.00464	0.00809	0.00574	0.00442	0.00750	0.00445	0.00502
577.15	10.015	-		0.00079	0.00214	0.00243	0.00117	0.00196	0.00235	0.00019	0.00041	0.00158	0.00046	0.00111	0.00109	0.00094	0.00175	0.00154	0.00027	0.00039	0.00124	0.00218	0.00273	0.00424
577.84	16.639	+		0.00000	0.00000	0.00000	0.00000	0.00042	0.00023	0.00016	0.00023	0.00065	0.00000	0.00010	0.00014	0.00012	0.00060	0.00008	0.00000	0.00082	0.00025	0.00039	0.00040	0.00013
579.14	8.41	+		0.00954	0.00875	0.00708	0.01710	0.01810	0.01250	0.01530	0.01610	0.01010	0.01407	0.01150	0.01100	0.01520	0.01824	0.01450	0.01920	0.01630	0.01130	0.01740	0.01110	0.01250
579.17	10.011	+		0.00200	0.00454	0.00568	0.00253	0.00445	0.00546	0.00000	0.00086	0.00381	0.00083	0.00513	0.00269	0.00269	0.00467	0.00400	0.00000	0.00070	0.00316	0.00390	0.00517	0.00754
580.96	16.197	-		0.00076	0.00042	0.00064	0.00080	0.00056	0.00011	0.00043	0.00056	0.00032	0.00036	0.00070	0.00008	0.00061	0.00096	0.00017	0.00014	0.00038	0.00027	0.00067	0.00034	0.00031
589.09	16.625	+		0.00029	0.00013	0.00000	0.00000	0.00018	0.00016	0.00019	0.00021	0.00038	0.00021	0.00000	0.00000	0.00000	0.00042	0.00020	0.00000	0.00000	0.00000	0.00000	0.00033	0.00000
589.16	11.082	-		0.00000	0.00000	0.00000	0.00447	0.00579	0.00498	0.00000	0.00000	0.00000	0.00000	0.00000	0.00000	0.00000	0.00000	0.00000	0.00000	0.00000	0.00000	0.00000	0.00000	0.00000
589.66	11.067	-		0.00000	0.00000	0.00000	0.00323	0.00427	0.00365	0.00000	0.00000	0.00000	0.00000	0.00000	0.00000	0.00000	0.00000	0.00000	0.00000	0.00000	0.00000	0.00000	0.00000	0.00000
591.11	8.042	-		0.00000	0.00000	0.00000	0.00000	0.00000	0.00006	0.00000	0.00000	0.00001	0.00000	0.00000	0.00000	0.00004	0.00008	0.00002	0.00000	0.00000	0.00000	0.00000	0.00000	0.00001
592.11	8.135	-		0.00000	0.00000	0.00008	0.00002	0.00008	0.00014	0.00000	0.00000	0.00017	0.00000	0.00000	0.00003	0.00042	0.00012	0.00036	0.00002	0.00004	0.00000	0.00000	0.00000	0.00003
593.13	8.014	-		0.00041	0.00045	0.00099	0.00084	0.00128	0.00114	0.00077	0.00082	0.00117	0.00047	0.00053	0.00078	0.00093	0.00148	0.00089	0.00074	0.00060	0.00075	0.00064	0.00053	0.00080
593.15	10.145	-		0.00145	0.00160	0.00186	0.00132	0.00129	0.00167	0.00230	0.00130	0.00112	0.00201	0.00114	0.00123	0.00125	0.00139	0.00129	0.00177	0.00114	0.00108	0.00164	0.00198	0.00300
595.14	8.038	+		0.00000	0.00000	0.00099	0.00020	0.00107	0.00133	0.00061	0.00073	0.00121	0.00000	0.00017	0.00046	0.00163	0.00203	0.00184	0.00000	0.00000	0.00036	0.00000	0.00031	0.00086
595.16	9.696	+		0.00130	0.00295	0.00388	0.00144	0.00245	0.00357	0.00187	0.00226	0.00341	0.00230	0.00323	0.00403	0.00525	0.00572	0.00629	0.00193	0.00255	0.00406	0.00207	0.00316	0.00485
595.16	9.396	-		0.00000	0.00061	0.00129	0.00000	0.00057	0.00087	0.00006	0.00002	0.00142	0.00008	0.00002	0.00000	0.00109	0.00128	0.00118	0.00000	0.00000	0.00000	0.00105	0.00103	0.00103
597.18	10.067	-		0.00090	0.00116	0.00122	0.00073	0.00083	0.00108	0.00083	0.00077	0.00119	0.00084	0.00083	0.00093	0.00047	0.00048	0.00039	0.00084	0.00082	0.00100	0.00064	0.00066	0.00089
599.10	10.307	-		0.00000	0.00000	0.00000	0.00000	0.00000	0.00000	0.00036	0.00030	0.00024	0.00023	0.00036	0.00049	0.00000	0.00000	0.00000	0.00023	0.00031	0.00043	0.00009	0.00018	0.00025
603.24	10.188	-		0.00005	0.00003	0.00000	0.00000	0.00000	0.00001	0.00000	0.00000	0.00001	0.00002	0.00010	0.00004	0.00000	0.00000	0.00000	0.00006	0.00012	0.00008	0.00000	0.00002	0.00009
609.12	7.363	-		0.00000	0.00008	0.00122	0.00054	0.00108	0.00103	0.00023	0.00011	0.00089	0.00016	0.00003	0.00006	0.00066	0.00100	0.00043	0.00008	0.00007	0.00004	0.00006	0.00005	0.00057
609.14	10.252	-		0.00242	0.00186	0.00336	0.00104	0.00110	0.00116	0.00196	0.00114	0.00233	0.00326	0.00140	0.00223	0.00173	0.00303	0.00154	0.00149	0.00099	0.00177	0.00121	0.00101	0.00117
610.12	7.441	-		0.00000	0.00000	0.00000	0.00000	0.00033	0.00022	0.00000	0.00000	0.00008	0.00000	0.00000	0.00000	0.00005	0.00020	0.00006	0.00000	0.00004	0.00008	0.00000	0.00000	0.00003
611.14	9.651	+		0.00000	0.00000	0.00000	0.00000	0.00000	0.00000	0.00000	0.00000	0.00000	0.00000	0.00000	0.00000	0.01240	0.00267	0.01030	0.00000	0.00000	0.00000	0.00000	0.00000	0.00000
611.14	8.513	-		0.00286	0.00350	0.00528	0.00267	0.00357	0.00283	0.00406	0.00349	0.00608	0.00408	0.00407	0.00417	0.00041	0.00122	0.00076	0.00364	0.00245	0.00407	0.00547	0.00439	0.00581
611.14	2.346	-		0.00015	0.00008	0.00007	0.00016	0.00007	0.00005	0.00018	0.00018	0.00007	0.00016	0.00021	0.00006	0.00007	0.00002	0.00004	0.00023	0.00012	0.00007	0.00021	0.00010	0.00005
615.09	10.005	-		0.00000	0.00000	0.00000	0.00000	0.00000	0.00000	0.00039	0.00039	0.00041	0.00024	0.00042	0.00093	0.00000	0.00000	0.00000	0.00004	0.00018	0.00075	0.00000	0.00009	0.00022
618.48	17.548	-		0.00002	0.00005	0.00004	0.00000	0.00003	0.00009	0.00004	0.00005	0.00007	0.00008	0.00008	0.00000	0.00006	0.00008	0.00002	0.00002	0.00001	0.00008	0.00004	0.00004	0.00014
625.14	9.919	-		0.00056	0.00040	0.00082	0.00012	0.00024	0.00021	0.00048	0.00030	0.00067	0.00098	0.00058	0.00102	0.00127	0.00160	0.00099	0.00059	0.00043	0.00080	0.00043	0.00036	0.00059
630.96	16.27	-		0.00011	0.00012	0.00015	0.00013	0.00028	0.00043	0.00008	0.00012	0.00013	0.00018	0.00011	0.00008	0.00030	0.00033	0.00019	0.00017	0.00024	0.00017	0.00011	0.00018	0.00015
631.09	9.634	-		0.00000	0.00000	0.00000	0.00000	0.00000	0.00000	0.00047	0.00080	0.00118	0.00006	0.00016	0.00099	0.00000	0.00000	0.00000	0.00006	0.00024	0.00127	0.00000	0.00000	0.00000
632.07	8.246	-		0.00007	0.00009	0.00005	0.00012	0.00006	0.00000	0.00012	0.00000	0.00006	0.00003	0.00020	0.00004	0.00046	0.00026	0.00040	0.00018	0.00015	0.00000	0.00000	0.00000	0.00000
633.07	8.252	-		0.00122	0.00157	0.00122	0.00175	0.00141	0.00042	0.00156	0.00088	0.00075	0.00116	0.00201	0.00176	0.00639	0.00296	0.00344	0.00251	0.00216	0.00110	0.00088	0.00086	0.00095
668.30	14.428	-		0.00001	0.00034	0.00018	0.00000	0.00000	0.00000	0.00000	0.00000	0.00010	0.00001	0.00010	0.00010	0.00000	0.00000	0.00002	0.00000	0.00001	0.00005	0.00016	0.00021	0.00045
671.61	18.04	+		0.03164	0.00541	0.00360	0.00729	0.02910	0.03200	0.00536	0.02970	0.00938	0.00429	0.03829	0.01760	0.00938	0.00479	0.03380	0.00129	0.00139	0.03410	0.01030	0.00682	0.00392
672.62	18.009	+		0.00049	0.03333	0.00000	0.00060	0.00211	0.01660	0.00080	0.00170	0.00141	0.00084	0.00232	0.00262	0.00221	0.00097	0.00287	0.02380	0.00216	0.00231	0.00173	0.00035	0.00108
687.57	17.394	-		0.00012	0.00024	0.00050	0.00021	0.00003	0.00012	0.00039	0.00018	0.00048	0.00032	0.00071	0.00007	0.00042	0.00017	0.00029	0.00032	0.00045	0.00053	0.00013	0.00084	0.00043
692.30	14.677	-		0.00005	0.00085	0.00053	0.00000	0.00000	0.00000	0.00000	0.00000	0.00022	0.00000	0.00033	0.00042	0.00000	0.00000	0.00000	0.00000	0.00000	0.00006	0.00030	0.00036	0.00053
692.80	14.666	-		0.00002	0.00058	0.00045	0.00000	0.00000	0.00000	0.00000	0.00000	0.00018	0.00000	0.00035	0.00045	0.00000	0.00000	0.00000	0.00000	0.00000	0.00003	0.00017	0.00032	0.00047

Supplementary Table S6. Selected peaks with the highest VIP values (>1) contributing to the construction of OPLS regression models in LC–MS datasets.

					(Name)	AT1	AT2	AT3	BF1	BF2	BF3	KM1	KM2	KM3	SM1	SM2	SM3	SR1	SR2	SR3	YB1	YB2	YB3	YM1	YM2	YM3
Variables	VIP	*m/z*	R.T.	Polar	(ORAC)	35,647	38,661	51,482	42,552	65,085	55,612	44,400	43,107	48,544	25,445	40,191	46,160	37,930	42,436	38,953	34,246	36,545	41,729	37,746	43,496	47,566
V01	6.37	441.08	9.463	-		0.03435	0.04153	0.04070	0.04440	0.04720	0.03910	0.02870	0.03040	0.03740	0.02393	0.03289	0.03600	0.03520	0.03370	0.03510	0.03320	0.03700	0.04040	0.03070	0.03380	0.04190
V02	6.12	457.07	8.917	-		0.10863	0.13601	0.12600	0.12700	0.13200	0.10300	0.10100	0.11800	0.13200	0.09022	0.12879	0.14100	0.10600	0.10078	0.10800	0.11900	0.13500	0.15100	0.11200	0.12100	0.13900
V03	4.78	175.11	2.169	+		0.02333	0.01038	0.00565	0.02800	0.01320	0.00865	0.02470	0.01690	0.00634	0.02769	0.01567	0.00914	0.01060	0.01285	0.01050	0.02670	0.01630	0.00533	0.02540	0.01580	0.00768
V04	4.52	303.06	10.533	+		0.10609	0.04191	0.05430	0.03130	0.00286	0.02780	0.00854	0.02980	0.00827	0.05213	0.00146	0.00307	0.00006	0.04873	0.00424	0.00000	0.03010	0.00784	0.03150	0.01330	0.01390
V05	4.10	671.61	18.04	+		0.03164	0.00541	0.00360	0.00729	0.02910	0.03200	0.00536	0.02970	0.00938	0.00429	0.03829	0.01760	0.00938	0.00479	0.03380	0.00129	0.00139	0.03410	0.01030	0.00682	0.00392
V06	3.71	523.14	11.193	-		0.00000	0.00000	0.00000	0.00988	0.01010	0.00667	0.00000	0.00001	0.00001	0.00000	0.00000	0.00000	0.00000	0.00000	0.00000	0.00000	0.00000	0.00000	0.00000	0.00000	0.00000
V07	3.57	443.09	9.484	+		0.00907	0.01118	0.01090	0.01330	0.01400	0.01230	0.00756	0.00800	0.00991	0.00620	0.00821	0.00950	0.01050	0.00993	0.01170	0.00875	0.00988	0.01140	0.00784	0.00943	0.01150
V08	3.46	471.08	9.368	-		0.00016	0.00027	0.00000	0.01010	0.00853	0.00859	0.00242	0.00282	0.00451	0.00002	0.00064	0.00021	0.00534	0.00613	0.00277	0.00081	0.00024	0.00064	0.00177	0.00238	0.00394
V09	3.26	531.14	11.069	-		0.00000	0.00000	0.00000	0.00748	0.01030	0.01320	0.00000	0.00000	0.00021	0.00000	0.00000	0.00083	0.00945	0.01068	0.00875	0.00000	0.00000	0.00000	0.00000	0.00000	0.00000
V10	3.12	287.05	10.431	+		0.01643	0.01673	0.01540	0.01060	0.01220	0.00793	0.02240	0.01870	0.01440	0.01904	0.01860	0.01630	0.01650	0.01155	0.00850	0.02630	0.02110	0.01680	0.01000	0.00858	0.00722
V11	3.00	337.16	2.346	+		0.00194	0.00201	0.00184	0.00287	0.00049	0.00158	0.00183	0.00522	0.00061	0.00769	0.00254	0.00123	0.00106	0.00194	0.00092	0.00183	0.00116	0.00056	0.00576	0.00218	0.00103
V12	2.95	589.16	11.082	-		0.00000	0.00000	0.00000	0.00447	0.00579	0.00498	0.00000	0.00000	0.00000	0.00000	0.00000	0.00000	0.00000	0.00000	0.00000	0.00000	0.00000	0.00000	0.00000	0.00000	0.00000
V13	2.79	523.64	11.16	-		0.00000	0.00000	0.00000	0.00532	0.00563	0.00373	0.00000	0.00000	0.00000	0.00000	0.00000	0.00000	0.00000	0.00000	0.00000	0.00000	0.00000	0.00000	0.00000	0.00000	0.00000
V14	2.77	579.17	10.011	+		0.00200	0.00454	0.00568	0.00253	0.00445	0.00546	0.00000	0.00086	0.00381	0.00083	0.00513	0.00269	0.00269	0.00467	0.00400	0.00000	0.00070	0.00316	0.00390	0.00517	0.00754
V15	2.75	387.11	1.833	-		0.00877	0.00555	0.00733	0.00561	0.00269	0.00516	0.00697	0.00503	0.00475	0.00950	0.00609	0.00465	0.00369	0.00447	0.00485	0.00544	0.00545	0.00337	0.00882	0.00681	0.00434
V16	2.75	672.62	18.009	+		0.00049	0.03333	0.00000	0.00060	0.00211	0.01660	0.00080	0.00170	0.00141	0.00084	0.00232	0.00262	0.00221	0.00097	0.00287	0.02380	0.00216	0.00231	0.00173	0.00035	0.00108
V17	2.67	293.17	13.644	-		0.01638	0.00206	0.01460	0.01560	0.00400	0.01450	0.02700	0.01680	0.00584	0.02567	0.00785	0.01820	0.00174	0.01563	0.00241	0.00513	0.01520	0.00090	0.00502	0.01260	0.00341
V18	2.65	341.11	1.837	-		0.00713	0.00454	0.00603	0.00442	0.00193	0.00410	0.00598	0.00420	0.00377	0.00817	0.00492	0.00356	0.00264	0.00338	0.00384	0.00438	0.00437	0.00264	0.00727	0.00549	0.00332
V19	2.64	371.10	8.233	+		0.00044	0.00089	0.00287	0.00000	0.00019	0.00000	0.00277	0.00125	0.00046	0.00765	0.00202	0.00071	0.00043	0.00039	0.00118	0.00251	0.00389	0.00161	0.00060	0.00511	0.00138
V20	2.61	457.38	8.914	-		0.00162	0.00120	0.00228	0.00200	0.00329	0.00160	0.00000	0.00000	0.00099	0.00077	0.00048	0.00233	0.00095	0.00176	0.00000	0.00000	0.00109	0.00130	0.00000	0.00208	0.00232
V21	2.58	277.18	13.655	+		0.01285	0.00164	0.01150	0.01200	0.00304	0.01150	0.02170	0.01390	0.00475	0.02166	0.00606	0.01350	0.00146	0.01216	0.00196	0.00404	0.01170	0.00043	0.00394	0.01070	0.00277
V22	2.55	307.08	2.367	+		0.00719	0.00519	0.00450	0.00594	0.00290	0.00276	0.00865	0.00875	0.00489	0.00635	0.00957	0.00585	0.00411	0.00247	0.00314	0.01040	0.00545	0.00432	0.00681	0.00521	0.00341
V23	2.55	459.09	8.914	+		0.05034	0.06195	0.05710	0.05680	0.05690	0.04770	0.04570	0.05550	0.05880	0.04328	0.05691	0.06350	0.05310	0.04898	0.05730	0.05430	0.06330	0.06750	0.04850	0.05560	0.06040
V24	2.54	589.66	11.067	-		0.00000	0.00000	0.00000	0.00323	0.00427	0.00365	0.00000	0.00000	0.00000	0.00000	0.00000	0.00000	0.00000	0.00000	0.00000	0.00000	0.00000	0.00000	0.00000	0.00000	0.00000
V25	2.39	531.64	11.073	-		0.00000	0.00000	0.00000	0.00375	0.00529	0.00679	0.00000	0.00000	0.00005	0.00000	0.00000	0.00035	0.00645	0.00519	0.00415	0.00000	0.00000	0.00000	0.00000	0.00000	0.00000
V26	2.35	337.09	9.563	-		0.00773	0.00222	0.00112	0.01220	0.01410	0.00512	0.01260	0.00871	0.00573	0.01594	0.01074	0.01050	0.00918	0.00654	0.00287	0.02220	0.01600	0.01070	0.01180	0.00672	0.00668
V27	2.32	563.14	9.646	-		0.00858	0.01150	0.01110	0.00228	0.00261	0.00257	0.00898	0.01100	0.00948	0.01008	0.01241	0.01280	0.00142	0.00251	0.00146	0.00871	0.01100	0.01190	0.00639	0.00625	0.00705
V28	2.29	455.09	10.021	-		0.00007	0.00009	0.00001	0.00401	0.00387	0.00436	0.00089	0.00102	0.00111	0.00050	0.00007	0.00005	0.00167	0.00195	0.00092	0.00015	0.00007	0.00017	0.00053	0.00094	0.00173
V29	2.28	565.15	9.616	+		0.01701	0.02221	0.02280	0.00699	0.00889	0.00795	0.01800	0.02070	0.02100	0.01978	0.02290	0.02600	0.00367	0.00661	0.00412	0.01920	0.02250	0.02700	0.01210	0.01420	0.01620
V30	2.21	425.04	8.708	-		0.00000	0.00000	0.00000	0.00193	0.00273	0.00218	0.00000	0.00008	0.00000	0.00000	0.00000	0.00000	0.00019	0.00013	0.00000	0.00000	0.00000	0.00000	0.00061	0.00065	0.00133
V31	2.04	273.07	9.482	+		0.00282	0.00344	0.00341	0.00458	0.00461	0.00412	0.00218	0.00221	0.00338	0.00212	0.00257	0.00322	0.00368	0.00326	0.00356	0.00289	0.00289	0.00313	0.00240	0.00282	0.00405
V32	2.03	158.08	2.173	+		0.00400	0.00178	0.00093	0.00480	0.00221	0.00147	0.00433	0.00294	0.00092	0.00489	0.00247	0.00148	0.00172	0.00192	0.00172	0.00468	0.00266	0.00058	0.00446	0.00255	0.00122
V33	2.01	303.05	10.143	+		0.01680	0.01634	0.02530	0.00684	0.00858	0.00981	0.01650	0.01140	0.02090	0.02086	0.01255	0.01780	0.01960	0.02113	0.02060	0.01440	0.01110	0.01750	0.00584	0.00525	0.00543
V34	1.96	524.13	11.003	-		0.00005	0.00051	0.00086	0.00304	0.00391	0.00473	0.00000	0.00000	0.00205	0.00048	0.00171	0.00128	0.00000	0.00000	0.00000	0.00006	0.00000	0.00254	0.00719	0.00744	0.00926
V35	1.90	305.06	8.485	-		0.04370	0.05356	0.06660	0.04580	0.05230	0.04240	0.05740	0.05240	0.07700	0.05677	0.05876	0.05900	0.02510	0.02700	0.01980	0.05470	0.04300	0.05830	0.07100	0.05880	0.07140
V36	1.76	291.08	9.161	+		0.01825	0.02057	0.02290	0.02310	0.02480	0.02180	0.02210	0.02110	0.02490	0.02177	0.01931	0.02100	0.01470	0.01717	0.01330	0.02360	0.01950	0.02200	0.02810	0.02310	0.02620
V37	1.71	577.15	10.015	-		0.00079	0.00214	0.00243	0.00117	0.00196	0.00235	0.00019	0.00041	0.00158	0.00046	0.00111	0.00109	0.00094	0.00175	0.00154	0.00027	0.00039	0.00124	0.00218	0.00273	0.00424
V38	1.70	451.11	9.424	+		0.00062	0.00057	0.00097	0.00118	0.00152	0.00153	0.00000	0.00102	0.00145	0.00000	0.00053	0.00064	0.00065	0.00053	0.00000	0.00081	0.00052	0.00082	0.00039	0.00067	0.00158
V39	1.67	447.09	10.626	-		0.00222	0.00148	0.00148	0.00205	0.00175	0.00100	0.00399	0.00165	0.00143	0.00321	0.00171	0.00192	0.01400	0.00731	0.00467	0.00456	0.00270	0.00256	0.00140	0.00090	0.00075
V40	1.66	609.12	7.363	-		0.00000	0.00008	0.00122	0.00054	0.00108	0.00103	0.00023	0.00011	0.00089	0.00016	0.00003	0.00006	0.00066	0.00100	0.00043	0.00008	0.00007	0.00004	0.00006	0.00005	0.00057
V41	1.65	456.07	8.752	-		0.00000	0.01236	0.01230	0.00128	0.01040	0.00191	0.00108	0.00400	0.00072	0.00813	0.00138	0.00461	0.00814	0.00848	0.00211	0.00000	0.01340	0.00343	0.00006	0.01340	0.00267
V42	1.61	309.17	13.655	-		0.00081	0.00013	0.00078	0.00081	0.00029	0.00102	0.00076	0.00111	0.00055	0.00330	0.00041	0.00089	0.00019	0.00096	0.00014	0.00047	0.00085	0.00003	0.00048	0.00076	0.00024
V43	1.59	371.10	2.303	+		0.00101	0.00113	0.00099	0.00254	0.00071	0.00109	0.00092	0.00169	0.00174	0.00227	0.00092	0.00047	0.00097	0.00189	0.00185	0.00240	0.00137	0.00243	0.00169	0.00145	0.00201
V44	1.59	449.11	10.434	+		0.00643	0.00665	0.00636	0.00435	0.00562	0.00279	0.00829	0.00647	0.00592	0.00766	0.00761	0.00712	0.00740	0.00520	0.00379	0.00963	0.00801	0.00697	0.00426	0.00363	0.00324
V45	1.59	431.10	10.767	-		0.00025	0.00225	0.00167	0.00002	0.00000	0.00000	0.00069	0.00361	0.00000	0.00167	0.00001	0.00591	0.00000	0.00000	0.00000	0.00164	0.00138	0.00049	0.00394	0.00000	0.00000
V46	1.59	516.13	11.309	-		0.00003	0.00048	0.00037	0.00286	0.00443	0.00524	0.00000	0.00000	0.00220	0.00036	0.00415	0.00055	0.00000	0.00000	0.00000	0.00019	0.00001	0.00308	0.01690	0.01690	0.01750
V47	1.56	309.09	11.188	+		0.00000	0.00000	0.00000	0.00195	0.00258	0.00291	0.00000	0.00000	0.00069	0.00000	0.00039	0.00038	0.00129	0.00216	0.00258	0.00000	0.00000	0.00034	0.00263	0.00247	0.00265
V48	1.47	457.08	12.008	-		0.00047	0.00062	0.00031	0.00082	0.00085	0.08060	0.00076	0.00009	0.00019	0.00011	0.00014	0.00020	0.08160	0.00032	0.10800	0.00060	0.00014	0.00031	0.00005	0.04410	0.00047
V49	1.44	441.26	9.463	-		0.00158	0.00193	0.00184	0.00203	0.00220	0.00181	0.00132	0.00135	0.00169	0.00103	0.00145	0.00163	0.00158	0.00152	0.00159	0.00148	0.00167	0.00188	0.00138	0.00150	0.00194
V50	1.44	304.95	7.828	-		0.00023	0.00178	0.00080	0.00047	0.00011	0.00009	0.00127	0.00015	0.00057	0.00430	0.00014	0.00112	0.00239	0.00007	0.00003	0.00023	0.00020	0.00116	0.00007	0.00455	0.00084
V51	1.39	595.14	8.038	+		0.00000	0.00000	0.00099	0.00020	0.00107	0.00133	0.00061	0.00073	0.00121	0.00000	0.00017	0.00046	0.00163	0.00203	0.00184	0.00000	0.00000	0.00036	0.00000	0.00031	0.00086
V52	1.38	289.07	8.913	+		0.01909	0.02251	0.02180	0.02290	0.02120	0.01910	0.01740	0.02120	0.02300	0.01670	0.02120	0.02300	0.02280	0.02082	0.02320	0.02080	0.02340	0.02490	0.01880	0.02070	0.02300
V53	1.36	175.03	2.07	-		0.00069	0.00000	0.00000	0.00043	0.00000	0.00000	0.00054	0.00000	0.00000	0.00122	0.00000	0.00000	0.00000	0.00000	0.00000	0.00085	0.00000	0.00000	0.00123	0.00000	0.00000
V54	1.36	387.15	13.099	+		0.00190	0.00253	0.00054	0.00091	0.00269	0.00000	0.00047	0.00110	0.00096	0.00000	0.00169	0.00142	0.00000	0.00252	0.00080	0.00164	0.00000	0.00218	0.00237	0.00069	0.00194
V55	1.35	593.13	8.014	-		0.00041	0.00045	0.00099	0.00084	0.00128	0.00114	0.00077	0.00082	0.00117	0.00047	0.00053	0.00078	0.00093	0.00148	0.00089	0.00074	0.00060	0.00075	0.00064	0.00053	0.00080
V56	1.35	463.08	10.291	-		0.00279	0.00273	0.00393	0.00175	0.00181	0.00180	0.00350	0.00155	0.00294	0.00485	0.00237	0.00402	0.01570	0.01513	0.01370	0.00288	0.00197	0.00316	0.00156	0.00120	0.00141
V57	1.29	534.07	16.729	+		0.00050	0.00118	0.00124	0.00103	0.00000	0.00035	0.00169	0.00105	0.00062	0.00202	0.00109	0.00114	0.00039	0.00102	0.00074	0.00080	0.00069	0.00063	0.00085	0.00125	0.00126
V58	1.23	353.08	8.816	-		0.00000	0.00000	0.00000	0.00308	0.00357	0.00171	0.00000	0.00000	0.00000	0.00073	0.00050	0.00000	0.00682	0.00531	0.00000	0.00086	0.00000	0.00000	0.00434	0.00372	0.00048
V59	1.23	173.10	2.166	-		0.00134	0.00048	0.00018	0.00210	0.00085	0.00046	0.00148	0.00084	0.00022	0.00200	0.00080	0.00045	0.00056	0.00066	0.00046	0.00164	0.00085	0.00026	0.00164	0.00079	0.00036
V60	1.21	439.15	9.8	+		0.00030	0.00055	0.00000	0.00208	0.00200	0.00198	0.00113	0.00087	0.00036	0.00056	0.00034	0.00040	0.00078	0.00015	0.00040	0.00034	0.00064	0.00057	0.00000	0.00000	0.00046
V61	1.20	457.26	8.914	-		0.00389	0.00504	0.00474	0.00455	0.00485	0.00377	0.00379	0.00444	0.00487	0.00332	0.00470	0.00520	0.00384	0.00363	0.00395	0.00444	0.00502	0.00555	0.00403	0.00437	0.00519
V62	1.17	441.48	9.463	-		0.00098	0.00126	0.00122	0.00135	0.00146	0.00118	0.00084	0.00088	0.00112	0.00065	0.00094	0.00105	0.00107	0.00101	0.00102	0.00104	0.00110	0.00121	0.00090	0.00100	0.00128
V63	1.17	456.09	9.672	-		0.00000	0.00000	0.00000	0.00093	0.00097	0.00103	0.00136	0.00018	0.00057	0.00000	0.00000	0.00000	0.00089	0.00066	0.00023	0.00000	0.00000	0.00113	0.00000	0.00023	0.00046
V64	1.16	516.63	11.303	-		0.00000	0.00027	0.00020	0.00149	0.00237	0.00276	0.00000	0.00000	0.00114	0.00020	0.00209	0.00026	0.00000	0.00000	0.00000	0.00005	0.00000	0.00164	0.00907	0.00895	0.00924
V65	1.15	319.04	9.903	+		0.00465	0.00460	0.00813	0.00255	0.00385	0.00465	0.00443	0.00358	0.00800	0.00712	0.00480	0.00916	0.01780	0.01814	0.01900	0.00504	0.00465	0.00919	0.00335	0.00293	0.00415
V66	1.14	461.16	9.975	-		0.00024	0.00032	0.00021	0.00127	0.00125	0.00119	0.00000	0.00017	0.00024	0.00002	0.00024	0.00027	0.00063	0.00013	0.00000	0.00022	0.00069	0.00055	0.00009	0.00019	0.00019
V67	1.13	487.09	10.315	+		0.00029	0.00073	0.00083	0.00000	0.00000	0.00034	0.00087	0.00000	0.00032	0.00164	0.00024	0.00034	0.00625	0.00419	0.00473	0.00090	0.00000	0.00065	0.00000	0.00000	0.00012
V68	1.12	203.10	13.789	+		0.00227	0.00025	0.00217	0.00208	0.00046	0.00155	0.00406	0.00257	0.00084	0.00416	0.00113	0.00266	0.00034	0.00215	0.00000	0.00072	0.00220	0.00020	0.00046	0.00200	0.00058
V69	1.12	511.80	16.729	+		0.00266	0.00272	0.00266	0.00154	0.00248	0.00114	0.00163	0.00057	0.00264	0.00079	0.00257	0.00240	0.00035	0.00302	0.00179	0.00206	0.00230	0.00200	0.00317	0.00159	0.00245
V70	1.11	307.59	2.371	+		0.00169	0.00090	0.00093	0.00161	0.00052	0.00066	0.00096	0.00136	0.00090	0.00114	0.00182	0.00063	0.00082	0.00068	0.00036	0.00205	0.00133	0.00055	0.00181	0.00125	0.00077
V71	1.10	480.28	16.7	+		0.00000	0.00061	0.00011	0.00039	0.00017	0.00022	0.00018	0.00000	0.00036	0.00107	0.00000	0.00026	0.00015	0.00046	0.00014	0.00072	0.00029	0.00046	0.00015	0.00059	0.00055
V72	1.09	181.07	8.027	+		0.00153	0.00057	0.00086	0.00274	0.00189	0.00265	0.00102	0.00055	0.00069	0.00057	0.00049	0.00062	0.00143	0.00116	0.00171	0.00100	0.00045	0.00043	0.00068	0.00077	0.00079
V73	1.09	483.13	11.389	-		0.00170	0.00303	0.00146	0.00031	0.00039	0.00011	0.00101	0.00149	0.00087	0.00121	0.00204	0.00162	0.00034	0.00013	0.00000	0.00195	0.00260	0.00117	0.00000	0.00000	0.00000
V74	1.09	407.01	8.022	+		0.00270	0.00158	0.00352	0.00037	0.00198	0.00169	0.00313	0.00434	0.00362	0.00109	0.00483	0.00426	0.00080	0.00273	0.00008	0.00185	0.00248	0.00336	0.00247	0.00189	0.00249
V75	1.08	343.07	7.139	-		0.00070	0.00075	0.00135	0.00141	0.00187	0.00055	0.00153	0.00177	0.00013	0.00047	0.00078	0.00230	0.00040	0.00096	0.00116	0.00064	0.00070	0.00365	0.00062	0.00029	0.00025
V76	1.08	406.07	10.402	+		0.00594	0.00596	0.00559	0.00483	0.00514	0.00384	0.00806	0.00768	0.00608	0.00622	0.00590	0.00525	0.00167	0.00137	0.00090	0.00910	0.00789	0.00709	0.00353	0.00275	0.00243
V77	1.08	441.17	9.17	+		0.00237	0.00123	0.00171	0.00293	0.00266	0.00233	0.00318	0.00303	0.00232	0.00320	0.00269	0.00232	0.00219	0.00227	0.00208	0.00418	0.00367	0.00306	0.00264	0.00220	0.00194
V78	1.07	457.73	8.911	-		0.00273	0.00349	0.00333	0.00321	0.00335	0.00258	0.00266	0.00301	0.00340	0.00228	0.00335	0.00362	0.00271	0.00250	0.00273	0.00307	0.00343	0.00384	0.00184	0.00303	0.00350
V79	1.07	387.18	13.039	+		0.00326	0.00289	0.00234	0.00391	0.00483	0.00234	0.00349	0.00387	0.00296	0.00325	0.00324	0.00352	0.00474	0.00313	0.00244	0.00297	0.00345	0.00406	0.00405	0.00267	0.00339
V80	1.07	304.91	8.354	-		0.00153	0.00109	0.00182	0.00083	0.00287	0.00077	0.00051	0.00041	0.00138	0.00103	0.00047	0.00048	0.00057	0.00051	0.00063	0.00118	0.00189	0.00058	0.00256	0.00155	0.00000
V81	1.06	457.48	8.912	-		0.00276	0.00347	0.00326	0.00320	0.00340	0.00256	0.00258	0.00297	0.00330	0.00230	0.00323	0.00362	0.00265	0.00250	0.00273	0.00304	0.00337	0.00381	0.00272	0.00304	0.00344
V82	1.06	465.10	10.166	+		0.00367	0.00366	0.00572	0.00157	0.00199	0.00225	0.00330	0.00212	0.00441	0.00508	0.00287	0.00430	0.00561	0.00608	0.00558	0.00298	0.00222	0.00364	0.00152	0.00120	0.00121
V83	1.02	367.29	17.959	+		0.00137	0.00059	0.00044	0.00000	0.00040	0.00145	0.00089	0.00066	0.00129	0.00254	0.00104	0.00022	0.00023	0.00156	0.00006	0.00122	0.00029	0.00022	0.00000	0.00227	0.00131
V84	1.01	533.13	10.045	-		0.00138	0.00174	0.00151	0.00017	0.00029	0.00030	0.00164	0.00260	0.00164	0.00119	0.00183	0.00186	0.00023	0.00061	0.00046	0.00165	0.00243	0.00223	0.00075	0.00076	0.00083

					(Name)	AT1	AT2	AT3	BF1	BF2	BF3	KM1	KM2	KM3	SM1	SM2	SM3	SR1	SR2	SR3	YB1	YB2	YB3	YM1	YM2	YM3
Variables	VIP	*m/z*	R.T.	Polar	(Polyphenol)	1.93	2.11	2.54	2.04	2.56	2.54	2.00	2.11	2.41	1.96	2.13	2.33	2.44	2.33	2.17	2.12	2.13	2.34	2.21	2.28	2.65
V01	9.06	303.06	10.533	+		0.10609	0.04191	0.05430	0.03130	0.00286	0.02780	0.00854	0.02980	0.00827	0.05213	0.00146	0.00307	0.00006	0.04873	0.00424	0.00000	0.03010	0.00784	0.03150	0.01330	0.01390
V02	6.63	175.11	2.169	+		0.02333	0.01038	0.00565	0.02800	0.01320	0.00865	0.02470	0.01690	0.00634	0.02769	0.01567	0.00914	0.01060	0.01285	0.01050	0.02670	0.01630	0.00533	0.02540	0.01580	0.00768
V03	6.31	457.07	8.917	-		0.10863	0.13601	0.12600	0.12700	0.13200	0.10300	0.10100	0.11800	0.13200	0.09022	0.12879	0.14100	0.10600	0.10078	0.10800	0.11900	0.13500	0.15100	0.11200	0.12100	0.13900
V04	4.26	441.08	9.463	-		0.03435	0.04153	0.04070	0.04440	0.04720	0.03910	0.02870	0.03040	0.03740	0.02393	0.03289	0.03600	0.03520	0.03370	0.03510	0.03320	0.03700	0.04040	0.03070	0.03380	0.04190
V05	4.10	293.17	13.644	-		0.01638	0.00206	0.01460	0.01560	0.00400	0.01450	0.02700	0.01680	0.00584	0.02567	0.00785	0.01820	0.00174	0.01563	0.00241	0.00513	0.01520	0.00090	0.00502	0.01260	0.00341
V06	3.78	277.18	13.655	+		0.01285	0.00164	0.01150	0.01200	0.00304	0.01150	0.02170	0.01390	0.00475	0.02166	0.00606	0.01350	0.00146	0.01216	0.00196	0.00404	0.01170	0.00043	0.00394	0.01070	0.00277
V07	3.57	459.09	8.914	+		0.05034	0.06195	0.05710	0.05680	0.05690	0.04770	0.04570	0.05550	0.05880	0.04328	0.05691	0.06350	0.05310	0.04898	0.05730	0.05430	0.06330	0.06750	0.04850	0.05560	0.06040
V08	3.19	305.06	8.485	-		0.04370	0.05356	0.06660	0.04580	0.05230	0.04240	0.05740	0.05240	0.07700	0.05677	0.05876	0.05900	0.02510	0.02700	0.01980	0.05470	0.04300	0.05830	0.07100	0.05880	0.07140
V09	2.94	387.11	1.833	-		0.00877	0.00555	0.00733	0.00561	0.00269	0.00516	0.00697	0.00503	0.00475	0.00950	0.00609	0.00465	0.00369	0.00447	0.00485	0.00544	0.00545	0.00337	0.00882	0.00681	0.00434
V10	2.85	307.08	2.367	+		0.00719	0.00519	0.00450	0.00594	0.00290	0.00276	0.00865	0.00875	0.00489	0.00635	0.00957	0.00585	0.00411	0.00247	0.00314	0.01040	0.00545	0.00432	0.00681	0.00521	0.00341
V11	2.81	579.17	10.011	+		0.00200	0.00454	0.00568	0.00253	0.00445	0.00546	0.00000	0.00086	0.00381	0.00083	0.00513	0.00269	0.00269	0.00467	0.00400	0.00000	0.00070	0.00316	0.00390	0.00517	0.00754
V12	2.80	516.13	11.309	-		0.00003	0.00048	0.00037	0.00286	0.00443	0.00524	0.00000	0.00000	0.00220	0.00036	0.00415	0.00055	0.00000	0.00000	0.00000	0.00019	0.00001	0.00308	0.01690	0.01690	0.01750
V13	2.80	341.11	1.837	-		0.00713	0.00454	0.00603	0.00442	0.00193	0.00410	0.00598	0.00420	0.00377	0.00817	0.00492	0.00356	0.00264	0.00338	0.00384	0.00438	0.00437	0.00264	0.00727	0.00549	0.00332
V14	2.78	158.08	2.173	+		0.00400	0.00178	0.00093	0.00480	0.00221	0.00147	0.00433	0.00294	0.00092	0.00489	0.00247	0.00148	0.00172	0.00192	0.00172	0.00468	0.00266	0.00058	0.00446	0.00255	0.00122
V15	2.55	337.16	2.346	+		0.00194	0.00201	0.00184	0.00287	0.00049	0.00158	0.00183	0.00522	0.00061	0.00769	0.00254	0.00123	0.00106	0.00194	0.00092	0.00183	0.00116	0.00056	0.00576	0.00218	0.00103
V16	2.48	524.13	11.003	-		0.00005	0.00051	0.00086	0.00304	0.00391	0.00473	0.00000	0.00000	0.00205	0.00048	0.00171	0.00128	0.00000	0.00000	0.00000	0.00006	0.00000	0.00254	0.00719	0.00744	0.00926
V17	2.41	456.07	8.752	-		0.00000	0.01236	0.01230	0.00128	0.01040	0.00191	0.00108	0.00400	0.00072	0.00813	0.00138	0.00461	0.00814	0.00848	0.00211	0.00000	0.01340	0.00343	0.00006	0.01340	0.00267
V18	2.29	289.07	8.913	+		0.01909	0.02251	0.02180	0.02290	0.02120	0.01910	0.01740	0.02120	0.02300	0.01670	0.02120	0.02300	0.02280	0.02082	0.02320	0.02080	0.02340	0.02490	0.01880	0.02070	0.02300
V19	2.25	443.09	9.484	+		0.00907	0.01118	0.01090	0.01330	0.01400	0.01230	0.00756	0.00800	0.00991	0.00620	0.00821	0.00950	0.01050	0.00993	0.01170	0.00875	0.00988	0.01140	0.00784	0.00943	0.01150
V20	2.19	287.05	10.431	+		0.01643	0.01673	0.01540	0.01060	0.01220	0.00793	0.02240	0.01870	0.01440	0.01904	0.01860	0.01630	0.01650	0.01155	0.00850	0.02630	0.02110	0.01680	0.01000	0.00858	0.00722
V21	2.19	456.81	8.912	-		0.00051	0.00168	0.00386	0.00077	0.00085	0.00097	0.00104	0.00000	0.00082	0.00101	0.00230	0.00169	0.00295	0.00239	0.00139	0.00220	0.00391	0.00447	0.00063	0.00213	0.00326
V22	2.12	307.08	8.494	+		0.04757	0.05722	0.06270	0.04760	0.05010	0.04610	0.05530	0.05680	0.07340	0.05734	0.05941	0.06140	0.03040	0.03241	0.02730	0.05570	0.04930	0.06060	0.06690	0.06100	0.06850
V23	2.09	577.15	10.015	-		0.00079	0.00214	0.00243	0.00117	0.00196	0.00235	0.00019	0.00041	0.00158	0.00046	0.00111	0.00109	0.00094	0.00175	0.00154	0.00027	0.00039	0.00124	0.00218	0.00273	0.00424
V24	2.04	516.63	11.303	-		0.00000	0.00027	0.00020	0.00149	0.00237	0.00276	0.00000	0.00000	0.00114	0.00020	0.00209	0.00026	0.00000	0.00000	0.00000	0.00005	0.00000	0.00164	0.00907	0.00895	0.00924
V25	2.03	595.16	9.696	+		0.00130	0.00295	0.00388	0.00144	0.00245	0.00357	0.00187	0.00226	0.00341	0.00230	0.00323	0.00403	0.00525	0.00572	0.00629	0.00193	0.00255	0.00406	0.00207	0.00316	0.00485
V26	1.94	289.09	8.574	+		0.00389	0.00000	0.00000	0.00229	0.00000	0.00000	0.01960	0.00000	0.00263	0.00152	0.02381	0.00226	0.00016	0.00000	0.00000	0.00583	0.00213	0.00000	0.00152	0.00000	0.00275
V27	1.90	291.08	9.161	+		0.01825	0.02057	0.02290	0.02310	0.02480	0.02180	0.02210	0.02110	0.02490	0.02177	0.01931	0.02100	0.01470	0.01717	0.01330	0.02360	0.01950	0.02200	0.02810	0.02310	0.02620
V28	1.89	671.61	18.04	+		0.03164	0.00541	0.00360	0.00729	0.02910	0.03200	0.00536	0.02970	0.00938	0.00429	0.03829	0.01760	0.00938	0.00479	0.03380	0.00129	0.00139	0.03410	0.01030	0.00682	0.00392
V29	1.75	672.62	18.009	+		0.00049	0.03333	0.00000	0.00060	0.00211	0.01660	0.00080	0.00170	0.00141	0.00084	0.00232	0.00262	0.00221	0.00097	0.00287	0.02380	0.00216	0.00231	0.00173	0.00035	0.00108
V30	1.71	173.10	2.166	-		0.00134	0.00048	0.00018	0.00210	0.00085	0.00046	0.00148	0.00084	0.00022	0.00200	0.00080	0.00045	0.00056	0.00066	0.00046	0.00164	0.00085	0.00026	0.00164	0.00079	0.00036
V31	1.68	457.38	8.914	-		0.00162	0.00120	0.00228	0.00200	0.00329	0.00160	0.00000	0.00000	0.00099	0.00077	0.00048	0.00233	0.00095	0.00176	0.00000	0.00000	0.00109	0.00130	0.00000	0.00208	0.00232
V32	1.59	524.63	10.999	-		0.00000	0.00020	0.00036	0.00079	0.00112	0.00172	0.00000	0.00000	0.00091	0.00017	0.00078	0.00058	0.00000	0.00000	0.00000	0.00001	0.00000	0.00104	0.00327	0.00345	0.00409
V33	1.57	553.10	11.027	+		0.00000	0.00000	0.00000	0.00014	0.00086	0.00138	0.00000	0.00000	0.00071	0.00000	0.00047	0.00010	0.00000	0.00000	0.00000	0.00000	0.00000	0.00064	0.00246	0.00278	0.00360
V34	1.48	479.08	9.902	-		0.00452	0.00550	0.01040	0.00231	0.00351	0.00448	0.00419	0.00297	0.00837	0.00722	0.00489	0.01100	0.02080	0.02112	0.02170	0.00430	0.00447	0.01000	0.00337	0.00285	0.00494
V35	1.48	433.11	10.076	+		0.00428	0.00710	0.00681	0.00233	0.00407	0.00334	0.00558	0.00596	0.00759	0.00472	0.00715	0.00941	0.00223	0.00360	0.00223	0.00424	0.00499	0.00824	0.00594	0.00702	0.00825
V36	1.48	595.14	8.038	+		0.00000	0.00000	0.00099	0.00020	0.00107	0.00133	0.00061	0.00073	0.00121	0.00000	0.00017	0.00046	0.00163	0.00203	0.00184	0.00000	0.00000	0.00036	0.00000	0.00031	0.00086
V37	1.47	203.10	13.789	+		0.00227	0.00025	0.00217	0.00208	0.00046	0.00155	0.00406	0.00257	0.00084	0.00416	0.00113	0.00266	0.00034	0.00215	0.00000	0.00072	0.00220	0.00020	0.00046	0.00200	0.00058
V38	1.47	451.11	9.424	+		0.00062	0.00057	0.00097	0.00118	0.00152	0.00153	0.00000	0.00102	0.00145	0.00000	0.00053	0.00064	0.00065	0.00053	0.00000	0.00081	0.00052	0.00082	0.00039	0.00067	0.00158
V39	1.44	289.07	7.642	+		0.00045	0.00064	0.00152	0.00032	0.00063	0.00025	0.00056	0.00366	0.00040	0.00052	0.00081	0.00090	0.00011	0.00024	0.00058	0.00134	0.00078	0.00026	0.00057	0.00117	0.00252
V40	1.42	166.08	6.24	+		0.00013	0.00054	0.00000	0.00011	0.00070	0.00010	0.00000	0.00033	0.00176	0.00019	0.00000	0.00048	0.00000	0.00066	0.00000	0.00041	0.00119	0.00128	0.00000	0.00011	0.00233
V41	1.42	471.08	9.368	-		0.00016	0.00027	0.00000	0.01010	0.00853	0.00859	0.00242	0.00282	0.00451	0.00002	0.00064	0.00021	0.00534	0.00613	0.00277	0.00081	0.00024	0.00064	0.00177	0.00238	0.00394
V42	1.41	273.07	9.482	+		0.00282	0.00344	0.00341	0.00458	0.00461	0.00412	0.00218	0.00221	0.00338	0.00212	0.00257	0.00322	0.00368	0.00326	0.00356	0.00289	0.00289	0.00313	0.00240	0.00282	0.00405
V43	1.39	535.32	16.826	+		0.00000	0.00000	0.00117	0.00000	0.00039	0.00000	0.00000	0.00015	0.00055	0.00000	0.00000	0.00016	0.00036	0.00063	0.00006	0.00067	0.00058	0.00000	0.00000	0.00048	0.00056
V44	1.38	236.11	13.642	-		0.00179	0.00017	0.00149	0.00154	0.00036	0.00149	0.00305	0.00170	0.00049	0.00286	0.00067	0.00190	0.00013	0.00158	0.00018	0.00043	0.00148	0.00003	0.00041	0.00124	0.00029
V45	1.37	545.11	11.341	+		0.00000	0.00000	0.00000	0.00051	0.00069	0.00119	0.00000	0.00000	0.00041	0.00000	0.00095	0.00000	0.00000	0.00000	0.00000	0.00000	0.00000	0.00061	0.00409	0.00458	0.00445
V46	1.37	609.12	7.363	-		0.00000	0.00008	0.00122	0.00054	0.00108	0.00103	0.00023	0.00011	0.00089	0.00016	0.00003	0.00006	0.00066	0.00100	0.00043	0.00008	0.00007	0.00004	0.00006	0.00005	0.00057
V47	1.36	221.15	13.642	-		0.00169	0.00013	0.00153	0.00168	0.00038	0.00151	0.00302	0.00173	0.00060	0.00286	0.00075	0.00187	0.00016	0.00156	0.00022	0.00048	0.00151	0.00000	0.00047	0.00129	0.00032
V48	1.35	406.07	10.402	+		0.00594	0.00596	0.00559	0.00483	0.00514	0.00384	0.00806	0.00768	0.00608	0.00622	0.00590	0.00525	0.00167	0.00137	0.00090	0.00910	0.00789	0.00709	0.00353	0.00275	0.00243
V49	1.35	457.26	8.914	-		0.00389	0.00504	0.00474	0.00455	0.00485	0.00377	0.00379	0.00444	0.00487	0.00332	0.00470	0.00520	0.00384	0.00363	0.00395	0.00444	0.00502	0.00555	0.00403	0.00437	0.00519
V50	1.34	280.98	15.993	-		0.00250	0.00390	0.00145	0.00000	0.00000	0.00143	0.00203	0.00138	0.00212	0.00134	0.00299	0.00000	0.00021	0.00358	0.00075	0.00115	0.00123	0.00108	0.00034	0.00205	0.00126
V51	1.34	469.13	9.527	+		0.00396	0.00254	0.00155	0.00258	0.00307	0.00244	0.00140	0.00245	0.00096	0.00197	0.00202	0.00143	0.00094	0.00144	0.00106	0.00187	0.00265	0.00151	0.00227	0.00275	0.00140
V52	1.31	307.59	2.371	+		0.00169	0.00090	0.00093	0.00161	0.00052	0.00066	0.00096	0.00136	0.00090	0.00114	0.00182	0.00063	0.00082	0.00068	0.00036	0.00205	0.00133	0.00055	0.00181	0.00125	0.00077
V53	1.31	309.17	13.655	-		0.00081	0.00013	0.00078	0.00081	0.00029	0.00102	0.00076	0.00111	0.00055	0.00330	0.00041	0.00089	0.00019	0.00096	0.00014	0.00047	0.00085	0.00003	0.00048	0.00076	0.00024
V54	1.30	337.09	9.563	-		0.00773	0.00222	0.00112	0.01220	0.01410	0.00512	0.01260	0.00871	0.00573	0.01594	0.01074	0.01050	0.00918	0.00654	0.00287	0.02220	0.01600	0.01070	0.01180	0.00672	0.00668
V55	1.29	595.16	9.396	-		0.00000	0.00061	0.00129	0.00000	0.00057	0.00087	0.00006	0.00002	0.00142	0.00008	0.00002	0.00000	0.00109	0.00128	0.00118	0.00000	0.00000	0.00000	0.00105	0.00103	0.00103
V56	1.29	531.14	11.069	-		0.00000	0.00000	0.00000	0.00748	0.01030	0.01320	0.00000	0.00000	0.00021	0.00000	0.00000	0.00083	0.00945	0.01068	0.00875	0.00000	0.00000	0.00000	0.00000	0.00000	0.00000
V57	1.27	309.09	11.188	+		0.00000	0.00000	0.00000	0.00195	0.00258	0.00291	0.00000	0.00000	0.00069	0.00000	0.00039	0.00038	0.00129	0.00216	0.00258	0.00000	0.00000	0.00034	0.00263	0.00247	0.00265
V58	1.25	175.03	2.07	-		0.00069	0.00000	0.00000	0.00043	0.00000	0.00000	0.00054	0.00000	0.00000	0.00122	0.00000	0.00000	0.00000	0.00000	0.00000	0.00085	0.00000	0.00000	0.00123	0.00000	0.00000
V59	1.21	139.04	8.917	+		0.00302	0.00400	0.00406	0.00363	0.00329	0.00342	0.00318	0.00363	0.00421	0.00314	0.00392	0.00432	0.00364	0.00295	0.00359	0.00374	0.00425	0.00446	0.00325	0.00400	0.00452
V60	1.19	593.13	8.014	-		0.00041	0.00045	0.00099	0.00084	0.00128	0.00114	0.00077	0.00082	0.00117	0.00047	0.00053	0.00078	0.00093	0.00148	0.00089	0.00074	0.00060	0.00075	0.00064	0.00053	0.00080
V61	1.16	411.02	8.385	-		0.00400	0.00582	0.00398	0.00128	0.00212	0.00219	0.00173	0.00302	0.00513	0.00101	0.00343	0.00443	0.00154	0.00220	0.00120	0.00179	0.00332	0.00381	0.00345	0.00308	0.00592
V62	1.16	197.81	2.147	-		0.00004	0.00025	0.00043	0.00060	0.00073	0.00051	0.00002	0.00031	0.00107	0.00025	0.00039	0.00069	0.00063	0.00047	0.00070	0.00000	0.00057	0.00102	0.00024	0.00010	0.00095
V63	1.16	205.10	8.092	+		0.00051	0.00100	0.00084	0.00060	0.00168	0.00067	0.00088	0.00159	0.00086	0.00046	0.00082	0.00063	0.00104	0.00111	0.00093	0.00110	0.00177	0.00093	0.00027	0.00058	0.00096
V64	1.12	531.64	11.073	-		0.00000	0.00000	0.00000	0.00375	0.00529	0.00679	0.00000	0.00000	0.00005	0.00000	0.00000	0.00035	0.00645	0.00519	0.00415	0.00000	0.00000	0.00000	0.00000	0.00000	0.00000
V65	1.12	483.13	11.389	-		0.00170	0.00303	0.00146	0.00031	0.00039	0.00011	0.00101	0.00149	0.00087	0.00121	0.00204	0.00162	0.00034	0.00013	0.00000	0.00195	0.00260	0.00117	0.00000	0.00000	0.00000
V66	1.09	371.10	8.233	+		0.00044	0.00089	0.00287	0.00000	0.00019	0.00000	0.00277	0.00125	0.00046	0.00765	0.00202	0.00071	0.00043	0.00039	0.00118	0.00251	0.00389	0.00161	0.00060	0.00511	0.00138
V67	1.09	611.14	8.513	-		0.00286	0.00350	0.00528	0.00267	0.00357	0.00283	0.00406	0.00349	0.00608	0.00408	0.00407	0.00417	0.00041	0.00122	0.00076	0.00364	0.00245	0.00407	0.00547	0.00439	0.00581
V68	1.09	414.07	10.116	+		0.00466	0.00452	0.00571	0.00226	0.00259	0.00314	0.00482	0.00379	0.00581	0.00504	0.00345	0.00413	0.00159	0.00208	0.00227	0.00421	0.00349	0.00540	0.00163	0.00129	0.00116
V69	1.07	425.04	8.708	-		0.00000	0.00000	0.00000	0.00193	0.00273	0.00218	0.00000	0.00008	0.00000	0.00000	0.00000	0.00000	0.00019	0.00013	0.00000	0.00000	0.00000	0.00000	0.00061	0.00065	0.00133
V70	1.06	293.50	13.643	-		0.00090	0.00002	0.00081	0.00087	0.00012	0.00082	0.00162	0.00096	0.00029	0.00156	0.00040	0.00105	0.00000	0.00087	0.00010	0.00025	0.00085	0.00000	0.00023	0.00071	0.00010
V71	1.03	191.03	2.388	-		0.00027	0.00044	0.00038	0.00150	0.00040	0.00059	0.00102	0.00077	0.00019	0.00112	0.00083	0.00040	0.00025	0.00028	0.00038	0.00116	0.00047	0.00021	0.00117	0.00065	0.00035
V72	1.02	195.81	2.152	-		0.00001	0.00026	0.00034	0.00044	0.00054	0.00043	0.00000	0.00021	0.00086	0.00018	0.00031	0.00056	0.00050	0.00039	0.00055	0.00000	0.00044	0.00071	0.00021	0.00008	0.00071
V73	1.01	319.04	9.903	+		0.00465	0.00460	0.00813	0.00255	0.00385	0.00465	0.00443	0.00358	0.00800	0.00712	0.00480	0.00916	0.01780	0.01814	0.01900	0.00504	0.00465	0.00919	0.00335	0.00293	0.00415
V74	1.01	457.73	8.911	-		0.00273	0.00349	0.00333	0.00321	0.00335	0.00258	0.00266	0.00301	0.00340	0.00228	0.00335	0.00362	0.00271	0.00250	0.00273	0.00307	0.00343	0.00384	0.00184	0.00303	0.00350
V75	1.01	457.48	8.912	-		0.00276	0.00347	0.00326	0.00320	0.00340	0.00256	0.00258	0.00297	0.00330	0.00230	0.00323	0.00362	0.00265	0.00250	0.00273	0.00304	0.00337	0.00381	0.00272	0.00304	0.00344

Supplementary Table S7. Selected peaks with the highest VIP values (>1) contributing to the construction of the polyphenol-predictive OPLS regression model in MALDI–MS datasets.

			(Name)	AT1	AT2	AT3	BF1	BF2	BF3	KM1	KM2	KM3	SM1	SM2	SM3	SR1	SR2	SR3	YB1	YB2	YB3	YM1	YM2	YM3
Variables	*m/z*	VIP	(Polyphenol)	1.93	2.11	2.54	2.04	2.56	2.54	2.00	2.11	2.41	1.96	2.13	2.33	2.44	2.33	2.17	2.12	2.13	2.34	2.21	2.28	2.65
V01	170.08	3.52		0.04360	0.00010	0.00010	0.00010	0.00010	0.00010	0.03820	0.00010	0.00010	0.00010	0.00010	0.00010	0.00010	0.00010	0.00010	0.00010	0.00010	0.00010	0.00010	0.00010	0.00010
V02	305.19	3.20		0.04070	0.05580	0.05710	0.04220	0.05710	0.05640	0.04840	0.05490	0.06950	0.04650	0.05640	0.06680	0.03360	0.03900	0.03480	0.04070	0.04740	0.05710	0.05080	0.05760	0.07250
V03	132.97	2.69		0.02420	0.01270	0.00620	0.02820	0.01910	0.01290	0.02080	0.01800	0.00780	0.02170	0.01770	0.00530	0.01890	0.02490	0.01750	0.01740	0.02430	0.01120	0.01720	0.01440	0.00760
V04	289.19	2.33		0.01740	0.02260	0.02420	0.02320	0.02910	0.02810	0.02010	0.02240	0.02800	0.01770	0.02130	0.02640	0.01710	0.02070	0.01810	0.01880	0.02030	0.02390	0.02260	0.02430	0.03130
V05	441.14	2.32		0.02020	0.02440	0.02740	0.01770	0.03010	0.03130	0.01920	0.01950	0.02150	0.01100	0.02310	0.02360	0.01930	0.01690	0.02310	0.01690	0.02090	0.02160	0.01820	0.02850	0.02830
V06	167.02	2.16		0.03350	0.04280	0.03930	0.03170	0.04120	0.04050	0.03320	0.04130	0.04190	0.02930	0.04160	0.04250	0.03550	0.03490	0.03890	0.03260	0.04010	0.04140	0.03630	0.04240	0.04400
V07	287.18	2.08		0.01840	0.02460	0.02430	0.01870	0.02530	0.02710	0.02030	0.02370	0.02530	0.01500	0.02350	0.02690	0.02110	0.02020	0.02290	0.01930	0.02240	0.02510	0.02070	0.02610	0.02680
V08	457.20	2.02		0.04110	0.04880	0.04720	0.03150	0.04970	0.04680	0.04130	0.04830	0.04580	0.02840	0.04720	0.04750	0.04080	0.03790	0.04940	0.04040	0.04800	0.04600	0.04200	0.05490	0.05130
V09	166.02	2.01		0.02010	0.02660	0.02670	0.02160	0.02760	0.02640	0.02350	0.02720	0.03310	0.02200	0.02880	0.02880	0.01890	0.02170	0.01980	0.02080	0.02380	0.02720	0.02470	0.02700	0.03320
V10	168.06	2.00		0.03590	0.04550	0.04220	0.03360	0.04430	0.04410	0.03490	0.04430	0.04190	0.03110	0.04410	0.04750	0.04240	0.04460	0.05190	0.03490	0.04410	0.04580	0.03820	0.04430	0.04410
V11	136.99	2.00		0.01580	0.01990	0.01870	0.01580	0.02200	0.02050	0.01750	0.02100	0.02330	0.01910	0.01810	0.02760	0.01610	0.02000	0.01610	0.01560	0.02080	0.02480	0.01980	0.01940	0.02440
V12	116.99	1.99		0.00660	0.00380	0.00010	0.00800	0.00310	0.00270	0.00510	0.00510	0.00260	0.00730	0.00770	0.00010	0.00710	0.00460	0.00390	0.00510	0.00560	0.00440	0.00600	0.00390	0.00010
V13	176.01	1.88		0.00480	0.00450	0.00010	0.00470	0.00010	0.00320	0.00440	0.00460	0.00010	0.00010	0.00010	0.00010	0.00450	0.00010	0.00400	0.00440	0.00500	0.00010	0.00490	0.00410	0.00010
V14	209.19	1.82		0.00850	0.00010	0.00010	0.00960	0.00010	0.00010	0.00760	0.00010	0.00010	0.00870	0.00010	0.00010	0.00850	0.00010	0.00010	0.00750	0.00870	0.00010	0.00780	0.00010	0.00010
V15	177.14	1.79		0.00720	0.00010	0.00010	0.00700	0.00010	0.00460	0.00660	0.00010	0.00010	0.00910	0.00010	0.00010	0.00740	0.00730	0.00640	0.00630	0.00010	0.00010	0.00740	0.00010	0.00010
V16	125.02	1.74		0.01650	0.02010	0.01800	0.01510	0.02120	0.02050	0.01570	0.01890	0.01940	0.01560	0.01750	0.02650	0.01800	0.01860	0.01840	0.01550	0.02150	0.02410	0.01780	0.01940	0.02110
V17	288.20	1.74		0.00940	0.01320	0.01380	0.00970	0.01400	0.01560	0.01010	0.01220	0.01400	0.00730	0.01250	0.01640	0.01010	0.01000	0.01210	0.00960	0.01130	0.01330	0.01040	0.01390	0.01460
V18	204.14	1.72		0.01590	0.00010	0.00010	0.00010	0.00010	0.00010	0.00010	0.00010	0.00010	0.00010	0.00010	0.00010	0.00010	0.00010	0.00010	0.01210	0.00010	0.00010	0.00010	0.00010	0.00010
V19	144.99	1.50		0.00010	0.00010	0.00010	0.01820	0.00830	0.00010	0.01330	0.00820	0.00010	0.02170	0.00010	0.00010	0.00010	0.00010	0.00010	0.01970	0.00010	0.00010	0.02430	0.00010	0.00010
V20	162.64	1.49		0.00010	0.00580	0.00530	0.00010	0.00590	0.00580	0.00010	0.00580	0.00560	0.00010	0.00580	0.00570	0.00010	0.00010	0.00580	0.00550	0.00010	0.00590	0.00630	0.00610	0.00600
V21	138.00	1.48		0.00920	0.01210	0.01130	0.00930	0.01290	0.01240	0.01030	0.01230	0.01350	0.01050	0.01100	0.01690	0.00840	0.01020	0.00870	0.00900	0.01190	0.01440	0.01110	0.01150	0.01440
V22	755.31	1.46		0.01170	0.01220	0.01200	0.00370	0.00640	0.00490	0.01450	0.01310	0.00850	0.01030	0.01520	0.00970	0.00010	0.00010	0.00010	0.01500	0.01350	0.00850	0.00920	0.00850	0.00430
V23	128.00	1.45		0.00430	0.00180	0.00010	0.01670	0.00640	0.00240	0.00740	0.00610	0.00010	0.01520	0.00340	0.00010	0.00530	0.00610	0.00370	0.00930	0.00550	0.00210	0.01210	0.00300	0.00240
V24	175.44	1.34		0.00200	0.00190	0.00170	0.01280	0.00010	0.00190	0.00180	0.00190	0.00170	0.01810	0.00010	0.00220	0.00010	0.00010	0.00010	0.00200	0.00200	0.00210	0.00230	0.00210	0.00010
V25	307.24	1.34		0.00440	0.00530	0.00500	0.00400	0.00010	0.00010	0.00490	0.00510	0.00010	0.00430	0.00010	0.00010	0.00390	0.00400	0.00370	0.00440	0.00490	0.00520	0.00510	0.00510	0.00010
V26	145.99	1.25		0.01070	0.00620	0.00510	0.01140	0.00740	0.00520	0.01190	0.01000	0.00710	0.01500	0.00810	0.00360	0.00960	0.00810	0.00630	0.01150	0.01060	0.00860	0.01290	0.00910	0.00760
V27	293.20	1.24		0.00010	0.00010	0.00260	0.00010	0.00010	0.00290	0.00010	0.00010	0.00010	0.00010	0.00010	0.00010	0.00010	0.00010	0.00010	0.00010	0.00010	0.00010	0.00010	0.00320	0.00340
V28	126.97	1.23		0.00850	0.00430	0.00010	0.00680	0.00510	0.00010	0.00660	0.00450	0.00010	0.00720	0.00360	0.00400	0.00650	0.00700	0.00380	0.00610	0.00690	0.00470	0.00730	0.00380	0.00400
V29	756.29	1.23		0.00410	0.00440	0.00440	0.00010	0.00010	0.00010	0.00530	0.00470	0.00290	0.00010	0.00550	0.00350	0.00010	0.00010	0.00010	0.00560	0.00480	0.00260	0.00310	0.00010	0.00010
V30	456.09	1.20		0.01440	0.01790	0.01790	0.01090	0.01810	0.01750	0.01540	0.01820	0.01610	0.00970	0.01720	0.01640	0.01380	0.01220	0.01770	0.01460	0.01730	0.01630	0.01450	0.02140	0.01900
V31	202.14	1.17		0.00740	0.00010	0.00010	0.00010	0.00010	0.00010	0.00010	0.00010	0.00010	0.00010	0.00010	0.00010	0.00010	0.00010	0.00010	0.00640	0.00010	0.00010	0.00010	0.00010	0.00010
V32	160.13	1.15		0.00960	0.00700	0.00420	0.00720	0.00630	0.00440	0.00770	0.00780	0.00500	0.00970	0.00690	0.00010	0.01030	0.00010	0.00530	0.00970	0.00900	0.00570	0.00990	0.00710	0.00560
V33	124.01	1.15		0.00800	0.01020	0.00890	0.00730	0.00960	0.00970	0.00710	0.00880	0.00920	0.00710	0.00850	0.01310	0.00810	0.00790	0.00830	0.00730	0.01050	0.01210	0.00800	0.00970	0.01040
V34	479.57	1.14		0.00830	0.01010	0.02060	0.00010	0.00010	0.00910	0.00880	0.00010	0.01390	0.00010	0.01020	0.01940	0.03020	0.02440	0.03290	0.00770	0.00010	0.01480	0.00010	0.00760	0.00860
V35	220.15	1.10		0.00340	0.00010	0.00010	0.00010	0.00010	0.00010	0.00250	0.00010	0.00010	0.00010	0.00010	0.00010	0.00010	0.00010	0.00010	0.00260	0.00010	0.00010	0.00010	0.00010	0.00010
V36	304.16	1.06		0.00720	0.00970	0.00970	0.00680	0.00890	0.00980	0.00810	0.00910	0.01010	0.00690	0.00880	0.01010	0.00660	0.00640	0.00680	0.00730	0.00790	0.00860	0.00810	0.00970	0.01070
V37	286.15	1.02		0.00710	0.00920	0.01010	0.00600	0.00820	0.00930	0.00850	0.00910	0.00980	0.00640	0.00970	0.01060	0.00940	0.00840	0.00920	0.00800	0.00800	0.00910	0.00770	0.00980	0.00920
V38	354.53	1.01		0.00190	0.00010	0.00010	0.00140	0.00010	0.00010	0.00170	0.00010	0.00010	0.00010	0.00010	0.00010	0.00010	0.00010	0.00010	0.00180	0.00010	0.00010	0.00200	0.00010	0.00010
